# The Arbitrarily Varying Relay Channel †

**DOI:** 10.3390/e21050516

**Published:** 2019-05-22

**Authors:** Uzi Pereg, Yossef Steinberg

**Affiliations:** Department of Electrical Engineering, Technion—Israel Institute of Technology, Haifa 32000, Israel

**Keywords:** arbitrarily varying channel, relay channel, decode-forward, Markov block code, minimax theorem, deterministic code, random code, symmetrizability

## Abstract

We study the arbitrarily varying relay channel, which models communication with relaying in the presence of an active adversary. We establish the cutset bound and partial decode-forward bound on the random code capacity. We further determine the random code capacity for special cases. Then, we consider conditions under which the deterministic code capacity is determined as well. In addition, we consider the arbitrarily varying Gaussian relay channel with sender frequency division under input and state constraints. We determine the random code capacity, and establish lower and upper bounds on the deterministic code capacity. Furthermore, we show that as opposed to previous relay models, the primitive relay channel has a different behavior compared to the non-primitive relay channel in the arbitrarily varying scenario.

## 1. Introduction

The relay channel was first introduced by van der Meulen [1] to describe point-to-point communication with the help of a relay, which receives a noisy version of the transmitter signal and transmits a signal of its own to the destination receiver. The relay channel is generally perceived as a fundamental building block for multihop networks (see e.g., [2,3], Chapter 16), where some nodes receive and transmit in order to assist the information flow between other nodes. The capacity of the relay channel is not known in general, however, Cover and El Gamal established the cutset upper bound, the decode-forward lower bound, and the partial decode-forward lower bound [4]. It was also shown in [4] that for the reversely degraded relay channel, direct transmission is capacity achieving. For the degraded relay channel, the decode-forward lower bound and the cutset upper bound coincide, thus characterizing the capacity for this model [4].

In general, the partial decode-forward lower bound is tighter than both direct transmission and decode-forward lower bounds. El Gamal and Zahedi [5] determined the capacity of the relay channel with orthogonal sender components, by showing that the partial decode-forward lower bound and cutset upper bound coincide. A variation of the relay channel, referred to as the primitive relay channel, was introduced by Kim [2], and attracted a lot of attention (see e.g., [6,7,8,9,10,11,12] and references therein). Recently, there has also been a growing interest in the Gaussian relay channel, as e.g., in [5,7,9,13,14,15,16] and references therein. In particular, El Gamal and Zahedi [5] introduced the Gaussian relay channel with sender frequency division (SFD), as a special case of a relay channel with orthogonal sender components. There are many other relaying scenarios, including secrecy [17,18], networking [15,19,20,21,22], parallel relaying [23,24,25], diamond channels [26,27,28], side information [29,30,31,32,33], etc.

In practice, the channel statistics are not necessarily known in exact, and they may even change over time. The arbitrarily varying channel (AVC) is an appropriate model to describe such a situation [34]. In real systems, such variations are caused by fading in wireless communication [35,36,37,38,39,40,41,42], memory faults in storage [43,44,45,46,47], malicious attacks on identification and authorization systems [48,49], etc. It is especially relevant to communication in the presence of an adversary, or a *jammer*, attempting to disrupt communication. Jamming attacks are not limited to point-to-point communication, and cause a major security concern for cognitive radio networks [50] and wireless sensor networks [42,51,52,53,54], for instance.

Considering the AVC without a relay, Blackwell et al. determined the random code capacity [34], i.e., the capacity achieved by stochastic-encoder stochastic-decoder coding schemes with common randomness. It was also demonstrated in [34] that the random code capacity is not necessarily achievable using deterministic codes. A well-known result by Ahlswede [55] is the dichotomy property of the AVC. Specifically, the deterministic code capacity either equals the random code capacity or else, it is zero. Subsequently, Ericson [56] and Csiszár and Narayan [57] established a simple single-letter condition, namely non-symmetrizability, which is both necessary and sufficient for the capacity to be positive. Ahlswede’s Robustification Technique (RT) is a useful technique for the AVC analysis, developed and applied to classical AVC settings [58,59]. Essentially, the RT uses a reliable code for the compound channel to construct a random code for the AVC applying random permutations to the codeword symbols. A continuing line of works on arbitrarily varying networks includes among others the arbitrarily varying broadcast channel [60,61,62,63,64,65], multiple-access channel [60,66,67,68,69,70,71,72,73,74,75], and wiretap channel [76,77,78,79,80,81,82,83,84]. The reference lists here are far from being exhaustive.

In this work, we introduce a new model, namely, the arbitrarily varying relay channel (AVRC). The AVRC combines the previous models, i.e., the relay channel and the AVC, and we believe that it is a natural problem to consider, in light of the jamming attacks on current and future networks, as mentioned above. In the analysis, we incorporate the block Markov coding schemes of [4] in Ahlswede’s Robustification and Elimination Techniques [55,59]. A straightforward application of Ahlswede’s RT fails to comply with the strictly causal relay transmission. In a recent work [85,86], by the authors of this paper, a modified RT technique was presented and applied to the point-to-point AVC with causal side information under input and state constraints, without a relay. This was the first time where the application of the RT exploited the structure of the original compound channel code to construct a random code for the AVC, as opposed to earlier work where the original code is treated as a “black box”. Here, we present another modification of the RT, which also exploits the structure of the original compound channel code, but in a different manner. The analysis also requires to redefine the compound channel, and we refer to the newly defined channel as the *block-compound relay channel*.

We establish the cutset upper bound and the full/partial decode-forward lower bound on the random code capacity of the AVRC. The random code capacity is determined in special cases of the degraded AVRC, the reversely degraded AVRC, and the AVRC with orthogonal sender components. Then, we give extended non-symmetrizability conditions under which the deterministic code capacity coincides with the random code capacity. We show by example that the deterministic code capacity can be strictly lower than the random code capacity of the AVRC. Then, we consider the Gaussian AVRC with SFD, under input and state constraints. The random code capacity is determined using the previous results, whereas the deterministic code capacity is lower and upper bounded using an independent approach. Specifically, we extend the techniques from [87], where Csiszár and Narayan determine the capacity of the Gaussian AVC under input and state constraint. It is shown that for low values on the input constraint, the deterministic code capacity can be strictly lower than the random code capacity, but yet non-zero.

Furthermore, we give similar bounds for the primitive AVRC, where there is a noiseless link between the relay and the receiver of limited capacity [2]. We find the capacity of the primitive counterpart of the Gaussian AVRC with SFD, in which case the deterministic and random code capacities coincide, regardless of the value of the input constraint. We deduce that Kim’s assertion—that “the primitive relay channel captures most essential features and challenges of relaying, and thus serves as a good testbed for new relay coding techniques” [2]—is not true in the arbitrarily varying scenario.

This work is organized as follows. In Section 2, the basic definitions and notation are provided. In Section 3, we give the main results on the general AVRC. The Gaussian AVRC with SFD is introduced in Section 4, and the main results are given in Section 5. The definition and results on the primitive AVRC are in Section 6.

## 2. Definitions

### 2.1. Notation

We use the following notation conventions throughout. Calligraphic letters X,S,Y,… are used for finite sets. Lowercase letters x,s,y,… stand for constants and values of random variables, and uppercase letters X,S,Y,… stand for random variables. The distribution of a random variable *X* is specified by a probability mass function (pmf) $P_{X}\left(x\right)=p\left(x\right)$ over a finite set X. The set of all pmfs over X is denoted by P(X). We use $x^{j}=\left(x_{1},x_{2},\ldots ,x_{j}\right)$ to denote a sequence of letters from X. A random sequence $X^{n}$ and its distribution $P_{X^{n}}\left(x^{n}\right)=p\left(x^{n}\right)$ are defined accordingly. For a pair of integers *i* and *j*, 1≤i≤j, we define the discrete interval [i:j]={i,i+1,…,j}. The notation $x=(x_{1},x_{2},\ldots ,x_{n})$ is used when it is understood from the context that the length of the sequence is *n*, and the $\ell^{2}$-norm of x is denoted by $∥x∥$.

### 2.2. Channel Description

A state-dependent discrete memoryless relay channel $\left(X,X_{1},S,W_{Y,Y_{1}|X,X_{1},S},Y,Y_{1}\right)$ consists of five sets, X, $X_{1}$, S, Y and $Y_{1}$, and a collection of conditional pmfs $W_{Y,Y_{1}|X,X_{1},S}$. The sets stand for the input alphabet, the relay transmission alphabet, the state alphabet, the output alphabet, and the relay input alphabet, respectively. The alphabets are assumed to be finite, unless explicitly said otherwise. The channel is memoryless without feedback, and therefore

(1) $$\begin{array}{c} W_{Y^{n},Y_{1}^{n}|X^{n},X_{1}^{n},S^{n}}\left(y^{n},y_{1}^{n}|x^{n},x_{1}^{n},s^{n}\right)=\prod_{i=1}^{n}W_{Y,Y_{1}|X,X_{1},S}\left(y_{i},y_{1,i}|x_{i},x_{1,i},s_{i}\right)\;. \end{array}$$

**Figure 1:**
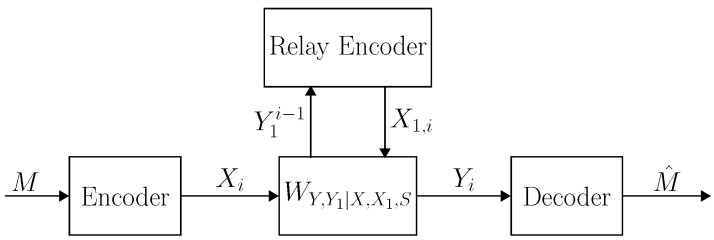
Communication over the arbitrarily varying relay channel $L=\{W_{Y,Y_{1}|X,X_{1},S}\}$. Given a message *M*, the encoder transmits $X^{n}=f\left(M\right)$. At time i∈[1:n], the relay transmits $X_{1,i}$ based on all the symbols of the past $Y_{1}^{i-1}$ and then receives a new symbol $Y_{1,i}$. The decoder receives the output sequence $Y^{n}$, and finds an estimate of the message $\;\hat{M}=g\left(Y^{n}\right)$.

Communication over a relay channel is depicted in Figure 1. Following [29], a relay channel $W_{Y,Y_{1}|X,X_{1},S}$ is called degraded if the channel can be expressed as

(2) $$\begin{array}{c} W_{Y,Y_{1}|X,X_{1},S}\left(y,y_{1}|x,x_{1},s\right)=W_{Y_{1}|X,X_{1},S}\left(y_{1}|x,x_{1},s\right)W_{Y|Y_{1},X_{1},S}\left(y|y_{1},x_{1},s\right)\;, \end{array}$$

and it is called reversely degraded if

(3) $$\begin{array}{c} W_{Y,Y_{1}|X,X_{1},S}\left(y,y_{1}|x,x_{1},s\right)=W_{Y|X,X_{1},S}\left(y|x,x_{1},s\right)W_{Y_{1}|Y,X_{1},S}\left(y_{1}|y,x_{1},s\right)\;. \end{array}$$

We say that the relay channel is *strongly* degraded or reversely degraded, if the respective definition holds such that the sender-relay marginal is independent of the state. That is, $W_{Y,Y_{1}|X,X_{1},S}$ is strongly degraded if $W_{Y,Y_{1}|X,X_{1},S}=W_{Y_{1}|X,X_{1}}W_{Y|Y_{1},X_{1},S}$, and similarly, $W_{Y,Y_{1}|X,X_{1},S}$ is strongly reversely degraded if $W_{Y,Y_{1}|X,X_{1},S}=W_{Y|X,X_{1},S}W_{Y_{1}|Y,X_{1}}$. For example, if $Y_{1}=X+Z$ and $Y=Y_{1}+X_{1}+S$, where *Z* is an independent additive noise, then $W_{Y,Y_{1}|X,X_{1},S}$ is strongly degraded. Whereas, if $Y=X+X_{1}+S$ and $Y_{1}=Y+Z$, then $W_{Y,Y_{1}|X,X_{1},S}$ is strongly reversely degraded.

The *arbitrarily varying relay channel* (AVRC) is a discrete memoryless relay channel $\left(X,X_{1},S,W_{Y,Y_{1}|X,X_{1},S},Y,Y_{1}\right)$ with a state sequence of unknown distribution, not necessarily independent nor stationary. That is, $S^{n}∼q\left(s^{n}\right)$ with an unknown joint pmf $q(s^{n})$ over $S^{n}$. In particular, $q(s^{n})$ can give mass 1 to some state sequence $s^{n}$. We use the shorthand notation $L=\{W_{Y,Y_{1}|X,X_{1},S}\}$ for the AVRC, where the alphabets are understood from the context.

To analyze the AVRC, we consider the *compound relay channel*. Different models of compound relay channels have been considered in the literature [30,88]. Here, we define the compound relay channel as a discrete memoryless relay channel $(X,X_{1},S,$ $W_{Y,Y_{1}|X,X_{1},S},Y,Y_{1})$ with a discrete memoryless state, where the state distribution q(s) is not known in exact, but rather belongs to a family of distributions Q, with Q⊆P(S). That is, $S^{n}∼\prod_{i=1}^{n}q\left(s_{i}\right)$, with an unknown pmf q∈Q over S. We use the shorthand notation $L^{Q}$ for the compound relay channel, where the transition probability $W_{Y,Y_{1}|X,X_{1},S}$ and the alphabets are understood from the context.

In the analysis, we also use the following model. Suppose that the user transmits B>0 blocks of length *n*, and the jammer is entitled to use a different state distribution $q_{b}\left(s\right)\in Q$ for every block b∈[1:B], while the encoder, relay and receiver are aware of this jamming scheme. In other words, every block is governed by a different memoryless state. We refer to this channel as the block-compound relay channel, denoted by $L^{Q\times B}$. Although this is a toy model, it is a useful tool for the analysis of the AVRC.

### 2.3. Coding

We introduce some preliminary definitions, starting with the definitions of a deterministic code and a random code for the AVRC L. Note that in general, the term ‘code’, unless mentioned otherwise, refers to a deterministic code.

**Definition** **1**  (A code, an achievable rate and capacity)**.** *A $\left(2^{nR},n\right)$ code for the AVRC L consists of the following; a message set $\left[1:2^{nR}\right]$, where it is assumed throughout that $2^{nR}$ is an integer, an encoder $f:\left[1:2^{nR}\right]\to X^{n}$, a sequence of n relaying functions $f_{1,i}:Y_{1}^{i-1}\to X_{1,i}$, i∈[1:n], and a decoding function $g:Y^{n}\to \left[1:2^{nR}\right]$.*

*Given a message $m\in [1:2^{nR}]$, the encoder transmits $x^{n}=f\left(m\right)$. At time i∈[1:n], the relay transmits $x_{1,i}=f_{1,i}\left(y_{1}^{i-1}\right)$ and then receives $y_{1,i}$. The relay codeword is given by $x_{1}^{n}=f_{1}^{n}\left(y_{1}^{n}\right)≜{\left(f_{1,i}\left(y_{1}^{i-1}\right)\right)}_{i=1}^{n}$. The decoder receives the output sequence $y^{n}$, and finds an estimate of the message $\hat{m}=g\left(y^{n}\right)$ (see Figure 1). We denote the code by $C=\left(f\left(\cdot \right),f_{1}^{n}\left(\cdot \right),g\left(\cdot \right)\right)$. Define the conditional probability of error of the code C given a state sequence $s^{n}\in S^{n}$ by*

(4) $$\begin{array}{c} P_{e|s^{n}}^{\left(n\right)}\left(C\right)=\frac{1}{2^{nR}}\sum_{m=1}^{2^{nR}}\sum_{\begin{array}{c} \left(y^{n},y_{1}^{n}\right)\;:\;g\left(y^{n}\right)\neq m \end{array}}\left[\prod_{i=1}^{n}W_{Y,Y_{1}|X,X_{1},S}\left(y_{i},y_{1,i}|f_{i}\left(m\right),f_{1,i}\left(y_{1}^{i-1}\right),s_{i}\right)\right]. \end{array}$$

*Now, define the average probability of error of C for some distribution $q\left(s^{n}\right)\in P\left(S^{n}\right)$,*

(5) $$\begin{array}{c} P_{e}^{\left(n\right)}\left(q,C\right)=\sum_{s^{n}\in S^{n}}q\left(s^{n}\right)\cdot P_{e|s^{n}}^{\left(n\right)}\left(C\right). \end{array}$$

*Observe that $P_{e}^{\left(n\right)}\left(q,C\right)$ is linear in q, and thus continuous. We say that C is a $\left(2^{nR},n,\varepsilon \right)$ code for the AVRC L if it further satisfies*

(6) $$\begin{array}{c} P_{e}^{\left(n\right)}\left(q,C\right)\leq \varepsilon \;,\;\mathrm{for}\;\mathrm{all}\;q\left(s^{n}\right)\in P\left(S^{n}\right)\;. \end{array}$$

*A rate R is called achievable if for every ε>0 and sufficiently large n, there exists a $\left(2^{nR},n,\varepsilon \right)$ code. The operational capacity is defined as the supremum of the achievable rates and it is denoted by C(L). We use the term ‘capacity’ referring to this operational meaning, and in some places we call it the deterministic code capacity in order to emphasize that achievability is measured with respect to deterministic codes.*

We proceed now to define the parallel quantities when using stochastic-encoders stochastic-decoder triplets with common randomness. The codes formed by these triplets are referred to as random codes.

**Definition** **2**  (Random code)**.** *A $\left(2^{nR},n\right)$ random code for the AVRC L consists of a collection of $\left(2^{nR},n\right)$ codes ${\left\{C_{\gamma }=\left(f_{\gamma },f_{1,\gamma }^{n},g_{\gamma }\right)\right\}}_{\gamma \in \Gamma }$, along with a probability distribution μ(γ) over the code collection *Γ*. We denote such a code by $C^{\;\Gamma }=\left(\mu ,\Gamma ,{\left\{C_{\gamma }\right\}}_{\gamma \in \Gamma }\right)$. Analogously to the deterministic case, a $\left(2^{nR},n,\varepsilon \right)$ random code has the additional requirement*

(7) $$\begin{array}{cc} P_{e}^{\left(n\right)}\left(q,C^{\;\Gamma }\right)=\sum_{\gamma \in \Gamma }\mu \left(\gamma \right)P_{e}^{\left(n\right)}\left(q,C_{\gamma }\right)\leq \varepsilon \;,\;\mathrm{for}\;\mathrm{all}\;q\left(s^{n}\right)\in P\left(S^{n}\right) & .\; \end{array}$$

*The capacity achieved by random codes is denoted by $C^{⋆}\left(L\right)$, and it is referred to as the random code capacity.*

## 3. Main Results—General AVRC

We present our results on the compound relay channel and the AVRC.

### 3.1. The Compound Relay Channel

We establish the cutset upper bound and the partial decode-forward lower bound for the compound relay channel. Consider a given compound relay channel $L^{Q}$. Let

(8) $$\begin{array}{cc} R_{CS}\left(L^{Q}\right)≜ & \underset{q\in Q}{\inf}\max_{p(x,x_{1})}\min\left\{I_{q}\left(X,X_{1};Y\right)\;,\;I_{q}\left(X;Y,Y_{1}|X_{1}\right)\right\}\;, \end{array}$$

and

(9) $$\begin{array}{c} R_{PDF}\left(L^{Q}\right)≜\max_{p(u,x,x_{1})}\min\{\underset{q\in Q}{\inf}I_{q}\left(U,X_{1};Y\right)+\underset{q\in Q}{\inf}I_{q}\left(X;Y|X_{1},U\right),\;\\ \;\;\;\;\;\;\;\underset{q\in Q}{\inf}I_{q}\left(U;Y_{1}|X_{1}\right)+\underset{q\in Q}{\inf}I_{q}\left(X;Y|X_{1},U\right)\}\;, \end{array}$$

where the subscripts ‘CS’ and ‘DF’ stand for ‘cutset’ and ‘partial decode-forward’, respectively.

**Lemma** **1.** *The capacity of the compound relay channel $L^{Q}$ is bounded by*

(10) $$\begin{array}{c} C\left(L^{Q}\right)\geq R_{PDF}\left(L^{Q}\right)\;, \end{array}$$

(11) $$\begin{array}{c} C^{⋆}\left(L^{Q}\right)\leq R_{CS}\left(L^{Q}\right). \end{array}$$

*Specifically, if $R<R_{PDF}\left(L^{Q}\right)$, then there exists a $\left(2^{nR},n,e^{-an}\right)$ block Markov code over $L^{Q}$ for sufficiently large n and some a>0.*

The proof of Lemma 1 is given in Appendix A. The achievability proof is based on block Markov coding interlaced with the partial decode-forward scheme. That is, the encoder sends a sequence of messages over multiple blocks. The message in each block consists of two components, a decode-forward component, and a direct transmission component, where only the former is decoded by the relay. The name ‘decode-forward component’ stands for the fact that the relay decodes this message component and sends its estimation forwards, to the destination receiver. Once the decoder has received all blocks, the decode-forward components are decoded backwards, i.e., starting with the message in the last block going backwards. Using the estimation of the decode-forward components, the direct transmission components are decoded forwards, i.e., starting with the message in the first block going forwards. The ambiguity of the state distribution needs to be dealt with throughout all of those estimations. In both decoding stages, the receiver performs joint typicality decoding using a set of types that “quantizes” the set Q of state distributions.

**Remark** **1.** *If the set of state distributions Q is convex, then the upper bound expression in the RHS of Equation (8) has a minmax form. On the other hand, in the lower bound expression in the RHS of Equation (9), the maximum comes first, and then we have multiple min terms, which makes this expression a lot more complicated than the classical partial decode-forward bound [4] (see also [3], Theorem 16.3), where Markov properties lead to a simpler expression. We note that this phenomenon (or one might say, disturbance) where the lower bound has multiple min terms is not exclusive to the AVRC. A noteworthy example is the arbitrarily varying wiretap channel [76,89], where the lower bound has the form of $\max[\min I_{q}\left(U;Y\right)-\max I_{q}\left(U;Z\right)]$. While the capacity of the classical wiretap channel is known, the arbitrarily varying counterpart has remained an open problem for several years.*

Observe that taking U=∅ in (9) gives the direct transmission lower bound,

(12) $$\begin{array}{cc} C(L^{Q})\geq & R_{PDF}\left(L^{Q}\right)\geq \max_{p(x,x_{1})}\underset{q\in Q}{\inf}I_{q}\left(X;Y|X_{1}\right)\;. \end{array}$$

Taking U=X in (9) results in a full decode-forward lower bound,

(13) $$\begin{array}{cc} C(L^{Q})\geq & R_{PDF}\left(L^{Q}\right)\geq \max_{p(x,x_{1})}\underset{q\in Q}{\inf}\min\left\{I_{q}\left(X,X_{1};Y\right)\;,\;I_{q}\left(X;Y_{1}|X_{1}\right)\right\}\;. \end{array}$$

This yields the following corollary. The corollary uses the terms of a strongly degraded relay channel, for which $W_{Y,Y_{1}|X,X_{1},S}=W_{Y_{1}|X,X_{1}}W_{Y|Y_{1},X_{1},S}$, and a strongly reversely degraded relay channel, for which $W_{Y,Y_{1}|X,X_{1},S}=W_{Y|X,X_{1},S}W_{Y_{1}|Y,X_{1}}$, as defined in Section 2.2.

**Corollary** **1.** *Let $L^{Q}$ be a compound relay channel, where Q is a compact convex set.*

*1.*  *If $W_{Y,Y_{1}|X,X_{1},S}$ is strongly reversely degraded, then*
  (14) $$\begin{array}{c} C\left(L^{Q}\right)=R_{PDF}\left(L^{Q}\right)=R_{CS}\left(L^{Q}\right)=\min_{q\in Q}\max_{p(x,x_{1})}I_{q}\left(X;Y|X_{1}\right)\;. \end{array}$$
*2.*  *If $W_{Y,Y_{1}|X,X_{1},S}$ is strongly degraded, then*
  (15) $$\begin{array}{c} C\left(L^{Q}\right)=R_{PDF}\left(L^{Q}\right)=R_{CS}\left(L^{Q}\right)=\max_{p(x,x_{1})}\min\left\{\min_{q\in Q}I_{q}\left(X,X_{1};Y\right)\;,\;I\left(X;Y_{1}|X_{1}\right)\right\}\;. \end{array}$$

The proof of Corollary 1 is given in Appendix B. Part 1 follows from the direct transmission and cutset bounds, (12) and (8), respectively, while part 2 is based on the full decode-forward and cutset bounds, (13) and (8), respectively, along with the convexity considerations in the remark below.

**Remark** **2.** *On a technical level, there are two purposes for considering the strongly degraded relay channel, for which the marginal channel to the relay is independent of the state, i.e., $W_{Y_{1}|X,X_{1},S}=W_{Y_{1}|X,X_{1}}$ (see Section 2.2). First, this ensures that $X-(X_{1},Y_{1})-Y$ form a Markov chain, without conditioning on S. Secondly, as pointed out in Remark 1, there is a difference between the order of the min and max in the lower and upper bounds (cf. (8) and (9)). Thereby, proving the capacity results of Corollary 1 above, we apply the minimax theorem. In general, a pointwise minimum of two convex functions may not necessarily yield a convex function. Nevertheless, having assumed that the relay channel is strongly degraded, the functional $G\left(p,q\right)=\min\{I_{q}\left(X,X_{1};Y\right)\;,\;I\left(X;Y_{1}|X_{1}\right)\}$ is* quasi*-convex in the state distribution, i.e.,*

(16) $$\begin{array}{c} G\left(p,\left(1-\alpha \right)q_{1}+\alpha q_{2}\right))\leq \max\left(G\left(p,q_{1}\right),G\left(p,q_{2}\right)\right)\;, \end{array}$$

**Figure 2:**
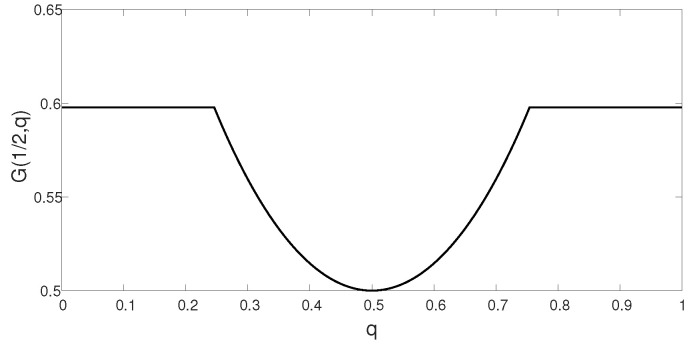
The functional $G\left(p,q\right)=\min\{I_{q}\left(X,X_{1};Y\right)\;,\;I\left(X;Y_{1}|X_{1}\right)\}$, for S∼Bernoulli(q), 0≤q≤1, as a function of *q*. The figure corresponds to Example 1, where $G\left(p,q\right)=\min\{1-\frac{1}{2}h\left(q\right)\;,\;1-h\left(\theta \right)\}$, for $p\left(x,x_{1}\right)=p\left(x\right)p\left(x_{1}\right)$, with $X∼\mathrm{Bernoulli}(\frac{1}{2})$ and $X_{1}∼\mathrm{Bernoulli}\left(\frac{1}{2}\right)$, and θ=0.08. Clearly, G(p,q) is not convex in *q*, but rather quasi-convex in *q*.

*for every $p\in P(X\times X_{1})$, $q_{1},q_{2}\in Q$, and 0≤α≤1. The quasi-convex shape is illustrated in Figure 2, which depicts G(p,q) for an example given in the sequel. By [90] (Theorem 3.4), the minimax theorem applies to quasi-convex functions as well, which alleviates the proof of Corollary 1.*

The following corollary is a direct consequence of Lemma 1 and it is significant for the random code analysis of the AVRC.

**Corollary** **2.** *The capacity of the block-compound relay channel $L^{Q\times B}$ is bounded by*

(17) $$\begin{array}{c} C\left(L^{Q\times B}\right)\geq R_{PDF}\left(L^{Q}\right)\;, \end{array}$$

(18) $$\begin{array}{c} C^{⋆}\left(L^{Q\times B}\right)\leq R_{CS}\left(L^{Q}\right). \end{array}$$

*Specifically, if $R<R_{PDF}\left(L^{Q}\right)$, then there exists a $\left(2^{nR},n,e^{-an}\right)$ block Markov code over $L^{Q\times B}$ for sufficiently large n and some a>0.*

The proof of Corollary 2 is given in Appendix C.

### 3.2. The AVRC

We give lower and upper bounds, on the random code capacity and the deterministic code capacity, for the AVRC L.

#### 3.2.1. Random Code Lower and Upper Bounds

The random code bounds below are obtained through a modified version of Ahlswede’s RT, using our results on the block-compound relay channel in Corollary 2. Define

(19) $$\begin{array}{c} R_{PDF}^{⋆}\left(L\right)≜R_{PDF}\left(L^{Q}\right){|}_{Q=P(S)}\;,\;R_{CS}^{⋆}\left(L\right)≜R_{CS}\left(L^{Q}\right){|}_{Q=P(S)}. \end{array}$$

**Theorem** **1.** *The random code capacity of an AVRC L is bounded by*

(20) $$\begin{array}{c} R_{PDF}^{⋆}\left(L\right)\leq C^{⋆}\left(L\right)\leq R_{CS}^{⋆}\left(L\right)\;. \end{array}$$

The proof of Theorem 1 is given in Appendix D. To prove Theorem 1 we modify Ahlswede’s RT. A straightforward application of Ahlswede’s RT fails to comply with the strictly causal relay transmission. Essentially, the RT uses a reliable code for the compound channel code to construct a random code for the AVC, applying random permutations to the transmitted codeword. However, the relay cannot apply permutations to its transmission, since at time i∈[1:n], the relay cannot compute $f_{1,j}\left(y_{1}^{j-1}\right)$, for j>i, as the relay encoder only knows the past received symbols $y_{1,1},\ldots ,y_{1,i-1}$, and does not have access to the symbols $y_{1,i},\ldots ,y_{1,j-1}$ which will be received in the future. To resolve this difficulty, we use a block Markov code for the block compound channel. In a block Markov coding scheme, the relay sends $x_{1,b}^{n}$ in block *b*, using the sequence of symbols $y_{1,b-1}^{n}$ received in the previous block. Since the entire sequence $y_{1,b-1}^{n}$ is known to the relay encoder, permutations can be applied to the transmission in each block separately. Hence, our proof exploits the structure of the original block-compound channel code to construct a random code for the AVRC, as opposed to classical works where the RT is used such that the original code is treated as a “black box” [59].

**Remark** **3.** *Block Markov coding with partial decode-forward is not a simple scheme by itself, and thus, using the RT requires careful attention. In particular, by close inspection of the proof of Theorem 1, one may recognize that the necessity of using the block-compound relay channel, rather than the standard compound channel, stems from the fact that for the AVRC, the state sequences may have completely different types in each block. For each block, we use the RT twice. First, the RT is applied to the probability of the backward decoding error, for the message component which is decoded by the relay. Then, it is applied to the probability of forward decoding error, for the message component which is transmitted directly.*

Together with Corollary 1, the theorem above yields another corollary.

**Corollary** **3.** *Let L be an AVRC.*

*1.*  *If $W_{Y,Y_{1}|X,X_{1},S}$ is strongly reversely degraded,*
  (21) $$\begin{array}{c} C^{⋆}\left(L\right)=R_{PDF}^{⋆}\left(L\right)=R_{CS}^{⋆}\left(L\right)=\min_{q(s)}\max_{p(x,x_{1})}I_{q}\left(X;Y|X_{1}\right). \end{array}$$
*2.*  *If $W_{Y,Y_{1}|X,X_{1},S}$ is strongly degraded,*
  (22) $$\begin{array}{c} C^{⋆}\left(L\right)=R_{PDF}^{⋆}\left(L\right)=R_{CS}^{⋆}\left(L\right)=\max_{p(x,x_{1})}\min\left\{\min_{q(s)}I_{q}\left(X,X_{1};Y\right)\;,\;I\left(X;Y_{1}|X_{1}\right)\right\}. \end{array}$$

Before we proceed to the deterministic code capacity, we note that Ahlswede’s Elimination Technique [55] can be applied to the AVRC as well. Hence, the size of the code collection of any reliable random code can be reduced to polynomial size.

#### 3.2.2. Deterministic Code Lower and Upper Bounds

In the next statements, we characterize the deterministic code capacity of the AVRC L. We consider conditions under which the deterministic code capacity is positive, and it coincides with the random code capacity, and conditions under which it is lower. For every $x_{1}\in X_{1}$, let $W_{1}\left(x_{1}\right)$ and $W(x_{1})$ denote the marginal AVCs from the sender to the relay and from the sender to the destination receiver, respectively,

(23) $$\begin{array}{c} W_{1}\left(x_{1}\right)=\left\{W_{Y_{1}|X,X_{1},S}\left(\cdot |\cdot ,x_{1},\cdot \right)\right\}\;,\;W\left(x_{1}\right)=\left\{W_{Y|X,X_{1},S}\left(\cdot |\cdot ,x_{1},\cdot \right)\right\}. \end{array}$$

See Figure 3.

Lemma 2 gives a condition under which the deterministic code capacity is the same as the random code capacity. The condition is given in terms of the marginal AVCs $W_{1}\left(x_{1}\right)$ and $W(x_{1})$.

**Lemma** **2.** *If the marginal sender-relay and sender-reciever AVCs have positive capacities, i.e., $C(W_{1}\left(x_{1,1}\right))>0$ and $C(W\left(x_{1,2}\right))$>0, for some $x_{1,1},x_{1,2}\in X_{1}$, then the capacity of the AVRC L is positive, and it coincides with the random code capacity, i.e.,* *$C\left(L\right)=C^{⋆}\left(L\right)>0$.*

The proof of Lemma 2 is given in Appendix E, extending Ahlswede’s Elimination Technique [55].

Next, we give a computable sufficient condition, under which the deterministic code capacity coincides with the random code capacity. For the point to point AVC, this occurs if and only if the channel is non-symmetrizable [56,57] (Definition 2). Our condition here is given in terms of an extended definition of symmetrizability, akin to [67] (Definition 3.2).

**Definition** **3.** *A state-dependent relay channel $W_{Y,Y_{1}|X,X_{1},S}$ is said to be* symmetrizable*-$X|X_{1}$ if for some conditional distribution J(s|x),*

(24) $$\begin{array}{c} \sum_{s\in S}W_{Y,Y_{1}|X,X_{1},S}\left(y,y_{1}|x,x_{1},s\right)J\left(s|\tilde{x}\right)=\sum_{s\in S}W_{Y,Y_{1}|X,X_{1},S}\left(y,y_{1}|\tilde{x},x_{1},s\right)J\left(s|x\right),\;\\ \forall \;x,\tilde{x}\in X\;,\;x_{1}\in X_{1}\;,\;y\in Y\;,\;y_{1}\in Y_{1}\;. \end{array}$$

*Equivalently, for every given $x_{1}\in X_{1}$, the channel $W_{\bar{Y}|X,X_{1},S}\left(\cdot |\cdot ,x_{1},\cdot \right)$ is symmetrizable, where $\bar{Y}=\left(Y,Y_{1}\right)$.*

A similar definition applies to the marginals $W_{Y|X,X_{1},S}$ and $W_{Y_{1}|X,X_{1},S}$. Note that symmetrizability of each of these marginals can be checked, without reference to whether the channel is degraded or strongly degraded.

**Corollary** **4.** *Let L be an AVRC.*

*1.*  *If $W_{Y|X,X_{1},S}$ and $W_{Y_{1}|X,X_{1},S}$ are non-symmetrizable-$X|X_{1}$, then $C\left(L\right)=C^{⋆}\left(L\right)>0$. In this case,*
  (25) $$\begin{array}{c} R_{PDF}^{⋆}\left(L\right)\leq C\left(L\right)\leq R_{CS}^{⋆}\left(L\right). \end{array}$$
*2.*  *If $W_{Y,Y_{1}|X,X_{1},S}$ is strongly reversely degraded, where $W_{Y_{1}|X,X_{1},S}$ is non-symmetrizable-$X|X_{1}$, then*
  (26) $$\begin{array}{c} C\left(L\right)=C^{⋆}\left(L\right)=R_{PDF}^{⋆}\left(L\right)=R_{CS}^{⋆}\left(L\right)=\min_{q(s)}\max_{p(x,x_{1})}I_{q}\left(X;Y|X_{1}\right). \end{array}$$
*3.*  *If $W_{Y,Y_{1}|X,X_{1},S}$ is strongly degraded, where $W_{Y|X,X_{1},S}$ is non-symmetrizable-$X|X_{1}$ and $W_{Y_{1}|X,X_{1}}\left(y_{1}|x,x_{1}\right)\neq W_{Y_{1}|X,X_{1}}\left(y_{1}|\tilde{x},x_{1}\right)$ for some $x,\tilde{x}\in X$, $x_{1}\in X_{1}$ and $y_{1}\in Y_{1}$, then*
  (27) $$\begin{array}{c} C\left(L\right)=C^{⋆}\left(L\right)=R_{PDF}^{⋆}\left(L\right)=R_{CS}^{⋆}\left(L\right)=\max_{p(x,x_{1})}\min\left\{\min_{q(s)}I_{q}\left(X,X_{1};Y\right)\;,\;I\left(X;Y_{1}|X_{1}\right)\right\}. \end{array}$$

The proof of Corollary 4 is given in Appendix F.

**Figure 3:**
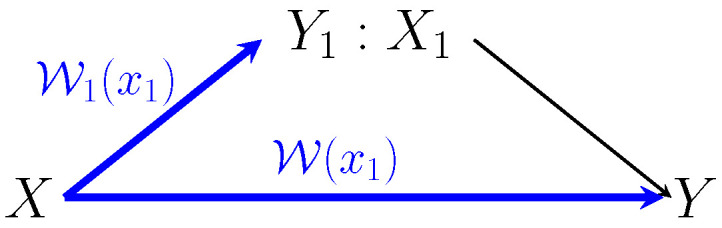
The marginals of the arbitrarily varying relay channel. For every relay transmission $x_{1}\in X_{1}$, the marginal sender-relay AVC is denoted by $W_{1}\left(x_{1}\right)=\left\{W_{Y_{1}|X,X_{1},S}\left(\cdot |\cdot ,x_{1},\cdot \right)\right\}$, and the marginal sender-receiver AVC is denoted by $W\left(x_{1}\right)=\{W_{Y|X,X_{1},S}\left(\cdot |\cdot ,x_{1},\cdot \right)$. A sufficient condition, under which the deterministic code capacity is the same as the random code capacity of the AVRC, is given in Lemma 2. This condition is also a sufficient condition for positive capacity, but as explained in Remark 4, it is not a necessary condition.

**Remark** **4.** *By Corollary 4, we have that non-symmetrizability of the marginal AVCs, $W_{1}\left(x_{1,1}\right)$ and $W(x_{1,2})$, for some $x_{1,1},x_{1,2}\in X_{1}$, is a sufficient condition for positive capacity (see Figure 3). This raises the question whether it is a necessary condition as well. In other words: If $W_{1}\left(x_{1}\right)$ and $W(x_{1})$ are symmetrizable for all $x_{1}\in X_{1}$, does that necessarily imply that the capacity is zero? The answer is no. We show this using a very simple example. Suppose that $Y_{1}=S$ and $Y=(X_{1},X+S)$, where all variables are binary. It is readily seen that for both $Y_{1}$ and Y, the input and the state are symmetric, for every given $X_{1}=x_{1}$. Hence, $W_{1}\left(x_{1}\right)$ and $W(x_{1})$ are symmetrizable for all $x_{1}\in X_{1}$. Nevertheless, we note that since the relay can send $X_{1}=Y_{1}=S$, this is equivalent to an AVC with state information at the decoder. As the decoder can use $X_{1}$ to eliminate the state, the capacity of this AVRC is C(L)=1. In Lemma 3 below, we give a stronger condition which is a necessary condition for positive capacity.*

**Remark** **5.** *Note that there are *4* symmetrizability cases in terms of the sender-relay channel $W_{Y_{1}|X,X_{1},S}$ and the sender-receiver channel $W_{Y|X,X_{1},S}$. For the case where $W_{Y_{1}|X,X_{1},S}$ and $W_{Y|X,X_{1},S}$ are both non-symmetrizable-$X|X_{1}$, the lemma above asserts that the capacity coincides with the random code capacity. In other cases, one may expect the capacity to be lower than the random code capacity. For instance, if $W_{Y|X,X_{1},S}$ is non-symmetrizable-$X|X_{1}$, while $W_{Y_{1}|X,X_{1},S}$* *is symmetrizable-$X|X_{1}$, then the capacity is positive by direct transmission. Furthermore, in this case, if the channel is reversely degraded, then the capacity coincides with the random code capacity. However, it remains in question whether this is true in general, when the channel is not reversely degraded.*

Next, we consider conditions under which the capacity is zero. Observe that if $W_{Y,Y_{1}|X,X_{1},S}$ is symmetrizable-$X|X_{1}$ then so are $W_{Y|X,X_{1},S}$ and $W_{Y_{1}|X,X_{1},S}$. Intuitively, if the AVRC is symmetrizable-$X|X_{1}$, then it is a poor channel. For example, say $Y_{1}=X+X_{1}+S$ and $Y=X\cdot X_{1}\cdot S$, with S=X. Then, the jammer can confuse the decoder by taking the state sequence $S^{n}$ to be some codeword. The following lemma validates this intuition.

**Lemma** **3.** *If the AVRC L is symmetrizable-$X|X_{1}$, then it has zero capacity, i.e., C(L)=0. Equivalently, non-symmetrizability-$X|X_{1}$ of the AVRC L is a necessary condition for positive capacity.*

Lemma 3 is proved in Appendix G, using an extended version of Ericson’s technique [56]. For a strongly degraded AVRC, we have a simpler symmetrizability condition under which the capacity is zero.

**Definition** **4.** *Let $W_{Y,Y_{1}|X,X_{1},S}=W_{Y_{1}|X,X_{1}}W_{Y|Y_{1},X_{1},S}$ be a strongly degraded relay channel. We say that $W_{Y,Y_{1}|X,X_{1},S}$ is* symmetrizable*-$X_{1}\times Y_{1}$ if for some conditional distribution $J(s|x_{1},y_{1})$,*

(28) $$\begin{array}{c} \sum_{s\in S}W_{Y|Y_{1},X_{1},S}\left(y|y_{1},x_{1},s\right)J\left(s|{\tilde{x}}_{1},{\tilde{y}}_{1}\right)=\sum_{s\in S}W_{Y|Y_{1},X_{1},S}\left(y|{\tilde{y}}_{1},{\tilde{x}}_{1},s\right)J\left(s|x_{1},y_{1}\right)\;,\\ \;\forall \;{\tilde{x}}_{1},x_{1}\in X_{1}\;,\;y\in Y\;,\;y_{1},{\tilde{y}}_{1}\in Y_{1}\;. \end{array}$$

*Equivalently, the channel $W_{Y|{\bar{Y}}_{1},S}$ is symmetrizable, where ${\bar{Y}}_{1}=\left(Y_{1},X_{1}\right)$.*

**Lemma** **4.** *If the AVRC L is strongly degraded and symmetrizable-$X_{1}\times Y_{1}$, then it has zero capacity, i.e., C(L)=0.*

Lemma 4 is proved in Appendix H. An example is given below.

**Example** **1.** *Consider a state-dependent relay channel $W_{Y,Y_{1}|X,X_{1},S}$, specified by*

$$\begin{array}{cc} Y_{1}= & X+Z\;\mathrm{mod}\;2\;,\\ Y= & X_{1}+S\;, \end{array}$$

*where $X=X_{1}=Z=S=Y_{1}=\left\{0,1\right\}$ and Y={0,1,2}, and the additive noise is distributed according to Z∼Bernoulli(θ), 0≤θ≤1. It is readily seen that $W_{Y,Y_{1}|X,X_{1},S}$ is strongly degraded and symmetrizable-$X_{1}\times Y_{1}$, by (2) and (28). In particular, (28) is satisfied with $J(s|x_{1},y_{1})=1$ for $s=x_{1}$, and $J(s|x_{1},y_{1})=0$ otherwise. Hence, by Lemma 4, the capacity is C(L)=0. On the other hand, we show that the random code capacity is given by $C^{⋆}\left(L\right)=\min\left\{\frac{1}{2},1-h\left(\theta \right)\right\}$, using Corollary 3. The derivation of the random code capacity is given in Appendix I.*

### 3.3. AVRC with Orthogonal Sender Components

Consider the special case of a relay channel $W_{Y,Y_{1}|X,X_{1},S}$ with orthogonal sender components [5]; [3] (Section 16.6.2), where $X=(X^{\prime },X^{\prime \prime })$ and

(29) $$\begin{array}{c} W_{Y,Y_{1}|X^{\prime },X^{\prime \prime },X_{1},S}\left(y,y_{1}|x^{\prime },x^{\prime \prime },x_{1},s\right)=W_{Y|X^{\prime },X_{1},S}\left(y|x^{\prime },x_{1},s\right)\cdot W_{Y_{1}|X^{\prime \prime },X_{1},S}\left(y_{1}|x^{\prime \prime },x_{1},s\right)\;. \end{array}$$

Here, we address the case where the channel output depends on the state only through the relay, i.e., $W_{Y|X^{\prime },X_{1},S}\left(y|x^{\prime },x_{1},s\right)=W_{Y|X^{\prime },X_{1}}\left(y|x^{\prime },x_{1}\right)$.

**Lemma** **5.** *Let L = $\{W_{Y|X^{\prime },X_{1}}$$W_{Y_{1}|X^{\prime \prime },X_{1},S}\}$ be an AVRC with orthogonal sender components. The random code capacity of L is given by*

(30) $$\begin{array}{c} C^{⋆}\left(L\right)=R_{PDF}^{⋆}\left(L\right)=R_{CS}^{⋆}\left(L\right)=\max_{p\left(x_{1}\right)p\left(x^{\prime }|x_{1}\right)p\left(x^{\prime \prime }|x_{1}\right)}\min\left\{I\left(X^{\prime },X_{1};Y\right)\;,\;\min_{q(s)}I_{q}\left(X^{\prime \prime };Y_{1}|X_{1}\right)+I\left(X^{\prime };Y|X_{1}\right)\right\}. \end{array}$$

*If $W_{Y_{1}|X^{\prime \prime },X_{1},S}$ is non-symmetrizable-$X^{\prime \prime }|X_{1}$, and $W_{Y|X^{\prime },X_{1}}\left(y|x^{\prime },x_{1}\right)\neq W_{Y|X^{\prime },X_{1}}\left(y|{\tilde{x}}^{\prime },x_{1}\right)$ for some $x_{1}\in X_{1}$, $x^{\prime },{\tilde{x}}^{\prime }\in X^{\prime }$, y∈Y, then the deterministic code capacity is given by $C\left(L\right)=R_{PDF}^{⋆}\left(L\right)=R_{CS}^{⋆}\left(L\right)$.*

The proof of Lemma 5 is given in Appendix J. To prove Lemma 5, we apply the methods of [5] to our results. Specifically, we use the partial decode-forward lower bound in Theorem 1, taking $U=X^{\prime \prime }$ (see (9) and (19)).

## 4. Gaussian AVRC with Sender Frequency Division

We give extended results for the Gaussian AVRC with sender frequency division (SFD), which is a special case of the AVRC with orthogonal sender components [5]. We determine the random code capacity of the Gaussian AVRC with SFD, and give lower and upper bounds on the deterministic code capacity. The derivation of the deterministic code bounds is mostly independent of our previous results, and it is based on the technique by [87]. The Gaussian relay channel $W_{Y,Y_{1}|X,X_{1},S}$ with SFD is a special case of a relay channel with orthogonal sender components [5], specified by

(31) $$\begin{array}{cc} Y_{1}= & X^{\prime \prime }+Z\;,\\ Y= & X^{\prime }+X_{1}+S\;, \end{array}$$

where the Gaussian additive noise $Z∼N(0,\sigma^{2})$ is independent of the channel state. As opposed to Lemma 5, the main channel here depends on the state, while the channel to the relay does not. In the case of a Gaussian channel, power limitations need to be accounted for, and thus, we consider the Gaussian relay channel under input and state constraints. Specifically, the user and the relay’s transmission are subject to input constraints Ω>0 and $\Omega_{1}>0$, respectively, and the jammer is under a state constraint Λ, i.e.,

(32) $$\begin{array}{c} \frac{1}{n}\sum_{i=1}^{n}\left(X_{i}^{\prime 2}+X_{i}^{\prime \prime 2}\right)\leq \Omega \;,\;\frac{1}{n}\sum_{i=1}^{n}X_{1,i}^{2}\leq \Omega_{1}\;w.p.\;1\;,\\ \frac{1}{n}\sum_{i=1}^{n}S_{i}^{2}\leq \Lambda \;w.p.\;1\;. \end{array}$$

We note that Ahlswede’s Elimination Technique cannot be used under a state constraint (see [57]). Indeed, if the jammer concentrates a lot of power on the shared randomness transmission, then this transmission needs to be robust against a state constraint that is higher than Λ. Thereby, the results given in Section 3.2.2 do not apply to the Gaussian AVRC under input and state constraints.

For the compound relay channel, the state constraint is in the average sense. That is, we say that the Gaussian compound relay channel $L^{Q}$ with SFD is under input constraints Ω and $\Omega_{1}$ and state constraint Λ if

(33) $$\begin{array}{c} \frac{1}{n}\sum_{i=1}^{n}\left(X_{i}^{\prime 2}+X_{i}^{\prime \prime 2}\right)\leq \Omega \;,\;\frac{1}{n}\sum_{i=1}^{n}X_{1,i}^{2}\leq \Omega_{1}\;,\;w.p.\;1\;,\\ Q=\{q\left(s\right)\;:\;ES^{2}\leq \Lambda \}\;. \end{array}$$

Coding definitions and notation are as follows. The definition of a code is similar to that of Section 2.3. The encoding function is denoted by $f=(f^{\prime },f^{\prime \prime })$, with $f^{\prime }:\left[1:2^{nR}\right]\to R^{n}$ and $f^{\prime \prime }:\left[1:2^{nR}\right]\to R^{n}$, and the relay encoding function is denoted by $f_{1}:R^{n}\to R^{n}$, where $f_{1,i}:R^{i-1}\to R$, for i∈[1:n]. The boldface notation indicates that the encoding functions produce sequences. Here, the encoder and the relay satisfy the input constraints ${∥f^{\prime }\left(m\right)∥}^{2}+{∥f^{\prime \prime }\left(m\right)∥}^{2}\leq n\Omega$ and ${∥f_{1}\left(y_{1}\right)∥}^{2}\leq n\Omega_{1}$ for all $m\in [1:2^{nR}]$ and $y_{1}\in R^{n}$. At time i∈[1:n], given a message $m\in [1:2^{nR}]$, the encoder transmits $\left(x_{i}^{\prime },x_{i}^{\prime \prime }\right)=\left(f_{i}^{\prime }\left(m\right),f_{i}^{\prime \prime }\left(m\right)\right)$, and the relay transmits $x_{1,i}=f_{1,i}\left(y_{1,1},\ldots ,y_{1,i-1}\right)$. The decoder receives the output sequence y, and finds an estimate $\hat{m}=g\left(y\right)$. A $\left(2^{nR},n,\varepsilon \right)$ code C for the Gaussian AVRC satisfies $P_{e|s}^{\left(n\right)}\left(C\right)\leq \varepsilon$, for all $s\in R^{n}$ with ${∥s∥}^{2}\leq n\Lambda$, where

(34) $$\begin{array}{c} P_{e|s}^{\left(n\right)}\left(C\right)=\frac{1}{2^{nR}}\sum_{m=1}^{2^{nR}}\int_{D{\left(m,s\right)}^{c}}\frac{1}{{\left(2\pi \sigma^{2}\right)}^{n\;/\;2}}e^{-{∥z∥}^{2}/2\sigma^{2}}\;dz\;, \end{array}$$

with

(35) $$\begin{array}{c} D\left(m,s\right)=\left\{z\in R^{n}:g\left(\;f^{\prime }\left(m\right)+f_{1}\left(f^{\prime \prime }\left(m\right)+z\right)+s\;\right)=m\right\}\;. \end{array}$$

Achivable rates, deterministic code capacity and random code capacity are defined as before. Next, we give our results on the Gaussian compound relay channel and the Gaussian AVRC with SFD.

## 5. Main Results—Gaussian AVRC with SFD

We give our results on the Gaussian compound and AVRC with SFD. The results on this compound relay channel and on the random code capacity of this AVRC are obtained through a straightforward extension of our previous results and derivations. However, the derivation of the deterministic code bounds is mostly independent of our previous results, and it is based on modifying the technique by Csiszär and Narayan in their paper on the Gaussian AVC [87].

### 5.1. Gaussian Compound Relay Channel

We determine the capacity of the Gaussian compound relay channel with SFD under input and state constraints. Let

(36) $$\begin{array}{c} F_{G}\left(\alpha ,\rho \right)≜\min\{\frac{1}{2}\log\left(1+\frac{\Omega_{1}+\alpha \Omega +2\rho \sqrt{\alpha \Omega }\sqrt{\Omega_{1}}}{\Lambda }\right)\;,\;\\ \;\frac{1}{2}\log\left(1+\frac{(1-\alpha )\Omega }{\sigma^{2}}\right)+\frac{1}{2}\log\left(1+\frac{(1-\rho^{2})\alpha \Omega }{\Lambda }\right)\}\;. \end{array}$$

**Lemma** **6.** *The capacity of the Gaussian compound relay channel with SFD, under input constraints *Ω* and $\Omega_{1}$ and state constraint *Λ*, is given by*

(37) $$\begin{array}{c} C\left(L^{Q}\right)=\max_{0\leq \alpha ,\rho \leq 1}F_{G}\left(\alpha ,\rho \right)\;, \end{array}$$

*and it is identical to the random code capacity, i.e., $C\left(L^{Q}\right)=C^{⋆}\left(L^{Q}\right)$.*

The proof of Lemma 6 is given in Appendix K, based on our results in the previous sections. The parameter 0≤α≤1 represents the fraction of input power invested in the transmission of the message component which is decoded by the relay, in the partial decode-forward coding scheme. Specifically, in the achievability proof in [5], αΩ and (1−α)Ω are the variances of $X^{\prime }$ and $X^{\prime \prime }$, respectively. The parameter ρ stands for the correlation coefficient between the decode-forward transmission $X^{\prime }$ and the relay transmission $X_{1}$.

### 5.2. Gaussian AVRC

We determine the random code capacity of the Gaussian AVRC with SFD under constraints.

**Theorem** **2.** *The random code capacity of the Gaussian AVRC with SFD, under input constraints *Ω* and $\Omega_{1}$ and state constraint *Λ*, is given by*

(38) $$\begin{array}{c} C^{⋆}\left(L\right)=C\left(L^{Q}\right)=\max_{0\leq \alpha ,\rho \leq 1}F_{G}\left(\alpha ,\rho \right). \end{array}$$

The proof of Theorem 2 is given in Appendix L. The proof follows the same considerations as in our previous results.

Next, we give lower and upper bounds on the deterministic code capacity of the Gaussian AVRC with SFD under constraints, obtained by generalizing the non-standard techniques by Csiszár and Narayan in their 1991 paper on the Gaussian AVC [87]. Define

(39) $$\begin{array}{c} \begin{array}{ccc} R_{G,low}\left(L\right)≜ & \max & F_{G}\left(\alpha ,\rho \right)\\ & \mathrm{subject}\;\mathrm{to} & 0\leq \alpha ,\rho \leq 1\;,\\ & & (1-\rho^{2})\alpha \Omega >\Lambda \;,\\ & & \frac{\Omega_{1}}{\Omega }{\left(\sqrt{\Omega_{1}}+\rho \sqrt{\alpha \Omega }\right)}^{2}>\Lambda +\left(1-\rho^{2}\right)\alpha \Omega \;. \end{array} \end{array}$$

and

(40) $$\begin{array}{cc} & \begin{array}{ccc} R_{G,up}\left(L\right)≜ & \max & F_{G}\left(\alpha ,\rho \right)\\ & \mathrm{subject}\;\mathrm{to} & 0\leq \alpha ,\rho \leq 1\;,\\ & & \Omega_{1}+\alpha \Omega +2\rho \sqrt{\alpha \Omega \cdot \Omega_{1}}\geq \Lambda \;. \end{array} \end{array}$$

It can be seen that $R_{G,low}\leq R_{G,up}$, since

(41) $$\begin{array}{c} \Omega_{1}+\alpha \Omega +2\rho \sqrt{\alpha \Omega \cdot \Omega_{1}}=\left(1-\rho^{2}\right)\alpha \Omega +{\left(\sqrt{\Omega_{1}}+\rho \sqrt{\alpha \Omega }\right)}^{2}\geq \left(1-\rho^{2}\right)\alpha \Omega \;. \end{array}$$

The analysis is based on the following lemma by [87].

**Lemma** **7** (see [87] (Lemma 1))**.** *For every ε>0, $8\sqrt{\varepsilon }<\eta <1$, K>2ε, and $M=2^{nR}$, with 2ε≤R≤K, and $n\geq n_{0}\left(\varepsilon ,\eta ,K\right)$, there exist M unit vectors $a\left(m\right)\in R^{n}$, m∈[1:M], such that for every unit vector $c\in R^{n}$ and 0≤θ,ζ≤1,*

(42) $$\begin{array}{c} |\left\{\tilde{m}\in \left[1:M\right]\;:\;\;\langle a\left(\tilde{m}\right),c\rangle \geq \theta \right\}|\leq 2^{n\left({\left[R+\frac{1}{2}\log\left(1-\theta^{2}\right)\right]}_{+}+\varepsilon \right)}\;, \end{array}$$

*and if θ≥η and $\theta^{2}+\zeta^{2}>1+\eta -2^{-2R}$, then*

(43) $$\begin{array}{cc} & \frac{1}{M}|\left\{m\in \left[1:M\right]\;:\;\;|\langle a\left(\tilde{m}\right),a\left(m\right)\rangle \left|\geq \theta \;,\;\right|\langle a\left(\tilde{m}\right),c\rangle |\geq \zeta \;,\;\mathrm{for}\;\mathrm{some}\;\tilde{m}\neq m\right\}|\leq 2^{-n\varepsilon }\;, \end{array}$$

*where ${\left[t\right]}_{+}=\max\left\{0,t\right\}$ and 〈·,·〉 denotes inner product.*

Intuitively, the lemma states that under certain conditions, a codebook can be constructed with an exponentially small fraction of “bad” messages, for which the codewords are non-orthogonal to each other and the state sequence.

**Theorem** **3.** *The deterministic code capacity of the Gaussian AVRC with SFD, under input constraints *Ω* and $\Omega_{1}$ and state constraint *Λ*, is bounded by*

(44) $$\begin{array}{c} R_{G,low}\left(L\right)\leq C\left(L\right)\leq R_{G,up}\left(L\right). \end{array}$$

The proof of Theorem 3 is given in Appendix M.

**Remark** **6.** *Csiszár and Narayan [87] have shown that for the classical Gaussian AVC, reliable decoding is guaranteed when the input constraint *Ω* is larger than the state constraint *Λ*. Here, we use a partial decode-forward coding scheme, where the message has two components, one which is decoded by the relay, and the other is transmitted directly. The respective optimization constraints $\frac{\Omega_{1}}{\Omega }{\left(\sqrt{\Omega_{1}}+\rho \sqrt{\alpha \Omega }\right)}^{2}>\Lambda +\left(1-\rho^{2}\right)\alpha \Omega$ and $(1-\rho^{2})\alpha \Omega >\Lambda$ in the RHS of (39), guarantee reliability for each decoding step.*

**Remark** **7.** *Csiszár and Narayan [87] have further shown that for the classical Gaussian AVC, if Ω≤Λ, the capacity is zero. The converse proof in [87] follows by considering a jammer who chooses the state sequence to be a codeword. Due to the symmetry between X and S, the decoder cannot distinguish between the transmitted codeword and the impostor sent by the jammer. Here, we consider a jammer who simulates $X^{\prime }+X_{1}$. Specifically, The jammer draws a codeword $X^{\prime }=f^{\prime }\left(\tilde{m}\right)$ uniformly at random, and then, generates a sequence ${\tilde{Y}}_{1}$ distributed according to the conditional distribution $P_{Y_{1}|M=\tilde{m}}$. If the sequence $\tilde{S}=f^{\prime }\left(\tilde{m}\right)+f_{1}\left({\tilde{Y}}_{1}\right)$ satisfies the state constraint *Λ*, then the jammer chooses $\tilde{S}$ as the state sequence. Defining αΩ, $\Omega_{1}$, and ρ as the empirical decode-forward transmission power, relay transmission power, and their correlation coefficient, respectively, we have that the state constraint ${∥\tilde{S}∥}^{2}\leq n\Lambda$ holds with high probability, if $\Omega_{1}+\alpha \Omega +2\rho \sqrt{\alpha \Omega \cdot \Omega_{1}}<\Lambda$. The details are in Appendix M.*

**Figure 4:**
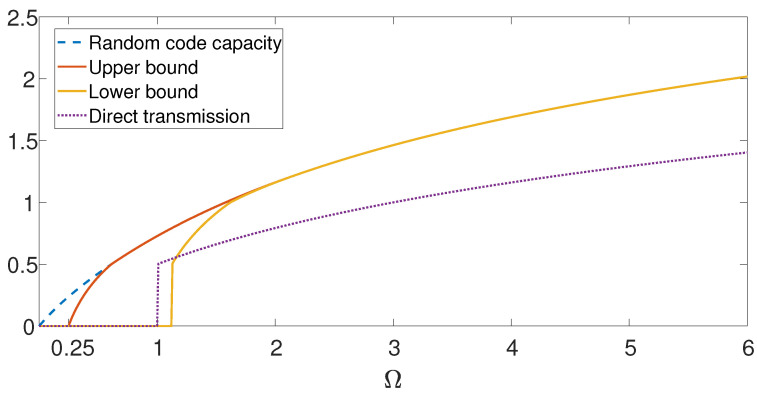
Bounds on the capacity of the Gaussian AVRC with sender frequency division. The dashed upper line depicts the random code capacity of the Gaussian AVRC as a function of the input constraint $\Omega =\Omega_{1}$, under state constraint Λ=1 and $\sigma^{2}=0.5$. The solid lines depict the deterministic code lower and upper bounds $R_{G,low}\left(L\right)$ and $R_{G,up}\left(L\right)$. The dotted lower line depicts the direct transmission lower bound.

Figure 4 depicts the bounds on the capacity of the Gaussian AVRC with SFD under input and state constraints, as a function of the input constraint $\Omega =\Omega_{1}$, under state constraint Λ=1 and $\sigma^{2}=0.5$. The top dashed line depicts the random code capacity of the Gaussian AVRC. The solid lines depict the deterministic code lower and upper bounds $R_{G,low}\left(L\right)$ and $R_{G,up}\left(L\right)$. For low values, $\Omega <\frac{\Lambda }{4}=0.25$, we have that $R_{G,up}\left(L\right)=0$, hence the deterministic code capacity is zero, and it is strictly lower than the random code capacity. The dotted lower line depicts the direct transmission lower bound, which is $F_{G}\left(1,0\right)$ for Ω>Λ, and zero otherwise [57]. For intermediate values of Ω, direct transmission is better than the lower bound in Theorem 3. Whereas, for high values of Ω, the optimization constraints in (39) and (40) are inactive, hence, our bounds are tight, and the capacity coincides with the random code capacity, i.e., $C\left(L\right)=C^{⋆}\left(L\right)=R_{G,low}\left(L\right)=R_{G,up}\left(L\right)$.

## 6. The Primitive AVRC

In this section, we give our results on the primitive AVRC [2], and then consider the Gaussian case. Part of the motivation given in [2] to consider the primitive relay channel was that the overall behavior and properties are the same as the non primitive (“regular”) relay channel. We show that this is not true in the arbitrarily varying scenario. In particular, the behavior of the primitive Gaussian AVRC with SFD is different compared to the non-primitive counterpart considered above.

### 6.1. Definitions and Notation

**Figure 5:**
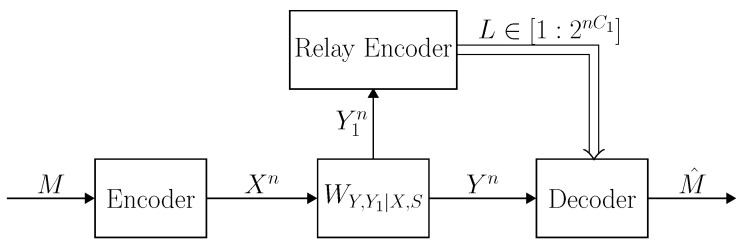
Communication over the primitive AVRC L. Given a message *M*, the encoder transmits $X^{n}=f\left(M\right)$. The relay receives $Y_{1}^{n}$ and sends $L=f_{1}\left(Y_{1}^{n}\right)$, where $f_{1}:Y_{1}^{n}\to \left[1:2^{nC_{1}}\right]$. The decoder receives both the channel output sequence $Y^{n}$ and the relay output *L*, and finds an estimate of the message $\;\hat{M}=g\left(Y^{n},L\right)$.

Consider a setup where the sender transmits information over state-dependent memoryless relay channel $W_{Y,Y_{1}|X,S}$, while there is a noiseless link of capacity $C_{1}>0$ between the relay and the receiver. Communication over a primitive relay channel is depicted in Figure 5. Given a message $M\in [1:2^{nR}]$, the encoder transmits $X^{n}=f\left(M\right)$ over the channel $W_{Y,Y_{1}|X,S}$, which is referred to as the primitive relay channel. The relay receives $Y_{1}^{n}$ and sends an index $L=f_{1}\left(Y_{1}^{n}\right)$ to the receiver, where $f_{1}:Y_{1}^{n}\to \left[1:2^{nC_{1}}\right]$. The decoder receives both the channel output sequence $Y^{n}$ and the relay output *L*, and finds an estimate of the message $\;\hat{M}=g\left(Y^{n},L\right)$. In accordance with the previous definitions, the primitive AVRC $L_{\mathrm{prim}}=\left\{W_{Y,Y_{1}|X,S}\right\}$ has a state sequence of unknown distribution, not necessarily independent nor stationary. The deterministic code capacity and the random code capacity are defined as before, and denoted by $C(L_{\mathrm{prim}})$ and $C^{⋆}\left(L_{\mathrm{prim}}\right)$, respectively.

### 6.2. Main Results—Primitive AVRC

We give our results on the primitive AVRC below. However, since the proofs are based on the same arguments as given for the non primitive AVRC, we omit the proofs of the results in this section. The details are given in [91].

Using similar arguments to those given for the non primitive relay channel, we obtain the following bounds on the random code capacity,

(45) $$\begin{array}{cc} R_{CS}^{⋆}≜ & \min_{q(s)}\max_{p(x)}\min\left\{I_{q}\left(X;Y\right)+C_{1}\;,\;I_{q}\left(X;Y,Y_{1}\right)\right\}\;, \end{array}$$

and

(46) $$\begin{array}{cc} R_{PDF}^{⋆}≜ & \max_{p(u,x)}\min\left\{\min_{q(s)}I_{q}\left(U;Y\right)+\min_{q(s)}I_{q}\left(X;Y|U\right)+C_{1}\;,\;\min_{q(s)}I_{q}\left(U;Y_{1}\right)+\min_{q(s)}I_{q}\left(X;Y|U\right)\right\}\;. \end{array}$$

**Theorem** **4.** *The random code capacity of a primitive AVRC $L_{\mathrm{prim}}$ is bounded by*

(47) $$\begin{array}{c} R_{PDF}^{⋆}\leq C^{⋆}\left(L_{\mathrm{prim}}\right)\leq R_{CS}^{⋆}. \end{array}$$

Those bounds have the same form as the cutset upper bound and the partical decode-forward lower bound in Section 3 (cf. (8), (9) and (45), (46)). As in Section 3, we can use the bounds above to determine the capacity in the strongly degraded and reversely degraded cases, based on the direct transmission lower bound (for U=∅), and the full decode-forward lower bound (for U=X).

**Corollary** **5.** *Let $L_{\mathrm{prim}}$ be a primitive AVRC.*

*1.*  *If $W_{Y,Y_{1}|X,S}$ is strongly reversely degraded, i.e., $W_{Y,Y_{1}|X,S}=W_{Y|X,S}W_{Y_{1}|Y}$, then*
  (48) $$\begin{array}{c} C^{⋆}\left(L_{\mathrm{prim}}\right)=\min_{q(s)}\max_{p(x)}I_{q}\left(X;Y\right)\;. \end{array}$$
*2.*  *If $W_{Y,Y_{1}|X,X_{1},S}$ is strongly degraded, i.e., $W_{Y,Y_{1}|X,X_{1},S}=W_{Y_{1}|X}W_{Y|Y_{1},S}$, then*
  (49) $$\begin{array}{c} C^{⋆}\left(L_{\mathrm{prim}}\right)=\max_{p(x)}\min\left\{\min_{q(s)}I_{q}\left(X;Y\right)+C_{1}\;,\;I\left(X;Y_{1}\right)\right\}\;. \end{array}$$

As for the deterministic code capacity, we give the following theorem.

**Theorem** **5.** *Let $L_{\mathrm{prim}}$ be a primitive AVRC.*

*1.*  *If $W_{Y_{1}|X,S}$ is non-symmetrizable, then $C\left(L_{\mathrm{prim}}\right)=C^{⋆}\left(L_{\mathrm{prim}}\right)$. In this case,*
  (50) $$\begin{array}{c} R_{PDF}^{⋆}\leq C\left(L_{\mathrm{prim}}\right)\leq R_{CS}^{⋆}\;. \end{array}$$
*2.*  *If $W_{Y,Y_{1}|X,S}$ is strongly reversely degraded, where $W_{Y_{1}|X,S}$ is non-symmetrizable, then*
  (51) $$\begin{array}{c} C\left(L_{\mathrm{prim}}\right)=\min_{q(s)}\max_{p(x)}I_{q}\left(X;Y\right)\;. \end{array}$$
*3.*  *If $W_{Y,Y_{1}|X,S}$ is strongly degraded, such that $W_{Y_{1}|X}\left(y_{1}|x\right)\neq W_{Y_{1}|X}\left(y_{1}|\tilde{x}\right)$ for some $x,\tilde{x}\in X$, $y_{1}\in Y_{1}$, then*
  (52) $$\begin{array}{c} C\left(L_{\mathrm{prim}}\right)=\max_{p(x)}\min\left\{\min_{q(s)}I_{q}\left(X;Y\right)+C_{1}\;,\;I\left(X;Y_{1}\right)\right\}\;. \end{array}$$
*4.*  *If $W_{\tilde{Y}|X,S}$ is symmetrizable, where $\tilde{Y}=\left(Y,Y_{1}\right)$, then $C(L_{\mathrm{prim}})=0$.*

The proof of Theorem 5 is available in [91]. To illustrate our results, we give the following example of a primitive AVRC.

**Example** **2.** *Consider a state-dependent primitive relay channel $W_{Y,Y_{1}|X,S}$, specified by*

$$\begin{array}{cc} Y_{1}= & X(1-S)\;,\\ Y= & X+S\;, \end{array}$$

*where $X=S=Y_{1}=\left\{0,1\right\}$, Y={0,1,2}, and $C_{1}=1$, i.e., the link between the relay and the receiver is a noiseless bit pipe. It can be seen that both the sender-relay and the sender-receiver marginals are symmetrizable. Indeed, $W_{Y|X,S}$ satisfies*

(53) $$\begin{array}{c} \sum_{s\in S}W_{Y|X,S}\left(y_{1}|x,s\right)J\left(s|\tilde{x}\right)=\sum_{s\in S}W_{Y|X,S}\left(y_{1}|\tilde{x},s\right)J\left(s|x\right)\;,\;x,\tilde{x}\in X\;,\;y\in Y\;, \end{array}$$

*with J(s|x)=1 for s=x, and J(s|x)=0 otherwise, while $W_{Y_{1}|X,S}$ satisfies (53) with J(s|x)=1 for s=1−x, and J(s|x)=0 otherwise. Nevertheless, the capacity of the primitive AVRC $L_{\mathrm{prim}}=\left\{W_{Y,Y_{1}|X,S}\right\}$ is $C(L_{\mathrm{prim}})=1$, which can be achieved using a code of length n=1, with f(m)=m, $f_{1}\left(y_{1}\right)=y_{1}$,*

(54) $$\begin{array}{c} g\left(y,\ell \right)=g\left(y,y_{1}\right)=\left\{\begin{array}{cc} 0 & y=0\\ 1 & y=2\\ y_{1} & y=1 \end{array}\right. \end{array}$$

*for $m,y_{1}\in \left\{0,1\right\}$ and y∈{0,1,2}. This example shows that even if the sender-relay and sender-receiver marginals are symmetrizable, the capacity may still be positive. We further note that the condition in part 4 of Theorem 5 implies that $W_{Y|X,S}$ and $W_{Y_{1}|X,S}$ are both symmetrizable, but not vice versa, as shown by this example. That is, as the capacity is positive, we have that $W_{\tilde{Y}|X,S}$ is non-symmetrizable, where $\tilde{Y}=\left(Y,Y_{1}\right)$, despite the fact that the marginals $W_{Y|X,S}$ and $W_{Y_{1}|X,S}$ are both symmetrizable.*

### 6.3. Primitive Gaussian AVRC

Consider the primitive Gaussian relay channel with SFD,

(55) $$\begin{array}{cc} Y_{1}= & X^{\prime \prime }+Z\;,\\ Y= & X^{\prime }+S\;, \end{array}$$

Suppose that input and state constraints are imposed as before, i.e., $\frac{1}{n}\sum_{i=1}^{n}\left(X_{i}^{\prime 2}+X_{i}^{\prime \prime 2}\right)\leq \Omega$ and $\frac{1}{n}\sum_{i=1}^{n}S_{i}^{2}\leq \Lambda$ with probability 1. The capacity of the primitive Gaussian AVRC with SFD, under input constraint Ω and state constraint Λ is given by

(56) $$\begin{array}{c} C\left(L_{\mathrm{prim}}\right)=C^{⋆}\left(L_{\mathrm{prim}}\right)=\max_{0\leq \alpha \leq 1}\left[\frac{1}{2}\log\left(1+\frac{\alpha \Omega }{\Lambda }\right)+\min\left\{C_{1},\frac{1}{2}\log\left(1+\frac{(1-\alpha )\Omega }{\Lambda }\right)\right\}\right]\;. \end{array}$$

This result is due to the following. Observe that one could treat this primitive AVRC as two independent channels, one from $X^{\prime }$ to *Y* and the other from $X^{\prime \prime }$ to $Y_{1}$, dividing the input power to αΩ and (1−α)Ω, respectively. Based on this observation, the random code direct part follows from [92]. Next, the deterministic code direct part follows from part 1 of Theorem 5, and the converse part follows straightforwardly from the cutset upper bound in Theorem 4.

## 7. Discussion

We have presented the model of the arbitrarily varying relay channel (AVRC), as a state dependent relay channel, where jamming attacks result in either a random or a deterministic state sequence, $S^{n}∼q\left(s^{n}\right)$, where the joint distribution $q(s^{n})$ is unknown and it is not necessarily of a product form. We have established the cutset upper bound and the partial decode-forward lower bound on the random code capacity of the AVRC. We have determined the random code capacity in special cases of the degraded AVRC, the reversely degraded AVRC, and the AVRC with orthogonal sender components. To do so, we used the direct transmission lower bound and the full decode-forward lower bound, along with quasi-convexity properties which are required in order to use the minimax theorem.

We have provided generalized symmetrizability conditions under which the deterministic code capacity coincides with the random code capacity. Specifically, we have shown that if the sender-relay and sender-receiver marginals are non-symmetrizable for a given relay transmission, then the capacity is positive. We further noted that this is a sufficient condition for positive capacity, which raises the question whether it is also a necessary condition. In other words, if those marginals are symmetrizable for every given relay transmission, does that necessarily imply that the capacity is zero? The answer is no, and we have refuted this assertion using a simple example, where the relay acts as a source of state information to the receiver. Then, we provided a stronger symmetrizability condition, which is necessary for the capacity to be positive. We have shown by example that the deterministic code capacity can be strictly lower than the random code capacity of the AVRC.

The Gaussian AVRC with sender frequency division (SFD) under input and state constraints is also addressed in this paper. The random code capacity is determined using the above results, whereas the deterministic code capacity is lower and upper bounded using an independent approach. Specifically, we extended the technique by Csiszár and Narayan in their 1991 paper on the Gaussian AVC [87]. We have shown that the deterministic code capacity can be strictly lower than the random code capacity, for low values on the input constraint.

Furthermore, we have considered the primitive AVRC, where there is a noiseless link between the relay and the receiver of limited capacity [2]. We tested Kim’s assertion that “the primitive relay channel captures most essential features and challenges of relaying, and thus serves as a good testbed for new relay coding techniques” [2]. We have shown that this assertion is not true in the arbitrarily varying scenario. Specifically, for the primitive Gaussian AVRC with SFD, the deterministic code capacity and the random code capacity are always the same, regardless of the value of the input constraint (see (56)), in contrast to our findings for the non primitive case, as demonstrated in Figure 4.

## Figures and Tables

**Figure A1:**
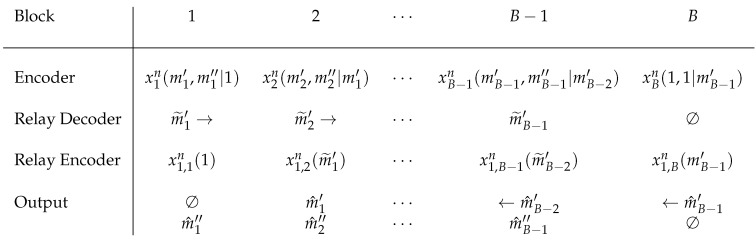
The partial decode-forward coding scheme. The block index b∈[1:B] is indicated at the top. In the following rows, we have the corresponding elements: (1) sequences transmitted by the encoder; (2) estimated messages at the relay; (3) sequences transmitted by the relay; (4) estimated messages at the destination decoder. The arrows in the second row indicate that the relay encodes forwards with respect to the block index, while the arrows in the fourth row indicate that the receiver decodes backwards.

## Abbreviations

The following abbreviations are used in this manuscript:

AVC	Arbitrarily varying channel
AVRC	Arbitrarily varying relay channel
DMC	Discrete memoryless channel
pmf	probability mass function
RT	Robustification technique
SFD	Sender frequency division
Eq.	Equation
RHS	Right hand side
LHS	Left hand side

## Appendix A. Proof of Lemma 1

### Appendix A.1. Partial Decode-Forward Lower Bound

We construct a block Markov code using the partial decode-forward scheme. That is, the encoder sends a sequence of messages over multiple blocks. The message in each block consists of two components, a decode-forward component, and a direct transmission component, where only the former is decoded by the relay. Once the decoder has received all blocks, the decode-forward components are decoded backwards, i.e., starting with the message in the last block going backwards. Using the estimation of the decode-forward components, the direct transmission components are decoded forwards, i.e., starting with the message in the first block going forwards. The ambiguity of the state distribution needs to be treated throughout all of those estimations. Hence, we use joint typicality with respect to a state type, which is “close” to some q∈Q. Let δ>0 be arbitrarily small. Define a set of state types ${\hat{Q}}_{n}$ by

(A1) $$\begin{array}{c} {\hat{Q}}_{n}=\left\{{\hat{P}}_{s^{n}}\;:\;s^{n}\in A^{\delta_{1}}\left(q\right)\;\;\mathrm{for}\;\mathrm{some}\;q\in Q\;\right\}\;, \end{array}$$

where

(A2) $$\begin{array}{c} \delta_{1}≜\frac{\delta }{2\cdot |S|}. \end{array}$$

Namely, ${\hat{Q}}_{n}$ is the set of types that are $\delta_{1}$-close to some state distribution q(s) in Q. A code C for the compound relay channel is constructed as follows.

The encoders use *B* blocks, each consists of *n* channel uses to convey (B−1) independent messages to the receiver. Furthermore, each message $M_{b}$, for b∈[1:B−1], is divided into two independent messages. That is, $M_{b}=\left(M_{b}^{\prime },M_{b}^{\prime \prime }\right)$, where $M_{b}^{\prime }$ and $M_{b}^{\prime \prime }$ are uniformly distributed, i.e.,

(A3) $$\begin{array}{c} M_{b}^{\prime }∼\mathrm{Unif}\left[1:2^{nR^{\prime }}\right]\;,\;M_{b}^{\prime \prime }∼\mathrm{Unif}\left[1:2^{nR^{\prime \prime }}\right]\;,\;\mathrm{with}\;R^{\prime }+R^{\prime \prime }=R\;, \end{array}$$

for b∈[1:B−1]. For convenience of notation, set $M_{0}^{\prime }=M_{B}^{\prime }\equiv 1$ and $M_{0}^{\prime \prime }=M_{B}^{\prime \prime }\equiv 1$. The average rate $\frac{B-1}{B}\cdot R$ is arbitrarily close to *R*.

*Codebook Generation*: Fix the distribution $P_{U,X,X_{1}}\left(u,x,x_{1}\right)$, and let

(A4) $$\begin{array}{c} P_{X,Y,Y_{1}|U,X_{1}}^{q}\left(x,y,y_{1}|u,x_{1}\right)=P_{X|U,X_{1}}\left(x|u,x_{1}\right)\sum_{s\in S}q\left(s\right)W_{Y,Y_{1}|X,X_{1},S}\left(y,y_{1}|x,x_{1},s\right). \end{array}$$

We construct *B* independent codebooks. For b∈[2:B−1], generate $2^{nR^{\prime }}$ independent sequences $x_{1,b}^{n}\left(m_{b-1}^{\prime }\right)$, $m_{b-1}^{\prime }\in \left[1:2^{nR^{\prime }}\right]$, at random, each according to $\prod_{i=1}^{n}P_{X_{1}}\left(x_{1,i}\right)$. Then, generate $2^{nR^{\prime }}$ sequences,

(A5) $$\begin{array}{cc} u_{b}^{n}\left(m_{b}^{\prime }|m_{b-1}^{\prime }\right)∼ & \prod_{i=1}^{n}P_{U|X_{1}}\left(u_{i}|x_{1,b,i}\left(m_{b-1}^{\prime }\right)\right)\;,\;m_{b}^{\prime }\in \left[1:2^{nR^{\prime }}\right]\;, \end{array}$$

conditionally independent given $x_{1,b}^{n}\left(m_{b-1}^{\prime }\right)$. Then, for every $m_{b}^{\prime }\in \left[1:2^{nR^{\prime }}\right]$, generate $2^{nR^{\prime \prime }}$ sequences,

(A6) $$\begin{array}{cc} x_{b}^{n}\left(m_{b}^{\prime },m_{b}^{\prime \prime }|m_{b-1}^{\prime }\right)∼ & \prod_{i=1}^{n}P_{X|U,X_{1}}\left(x_{i}|u_{b,i}\left(m_{b}^{\prime }|m_{b-1}^{\prime }\right),x_{1,b,i}\left(m_{b-1}^{\prime }\right)\right)\;,\;m_{b}^{\prime \prime }\in \left[1:2^{nR^{\prime \prime }}\right]\;, \end{array}$$

conditionally independent given $\left(u_{b}^{n}\left(m_{b}^{\prime }|m_{b-1}^{\prime }\right),x_{1,b}^{n}\left(m_{b-1}^{\prime }\right)\right)$. We have thus generated B−2 independent codebooks,

(A7) $$\begin{array}{c} F_{b}=\left\{\left(x_{1,b}^{n}\left(m_{b-1}^{\prime }\right),u_{b}^{n}\left(m_{b}^{\prime }|m_{b-1}^{\prime }\right),x_{b}^{n}\left(m_{b}^{\prime },m_{b}^{\prime \prime }|m_{b-1}^{\prime }\right)\right)\;:\;m_{b-1}^{\prime },m_{b}^{\prime }\in \left[1:2^{nR^{\prime }}\right]\;,\;m_{b}^{\prime \prime }\in \left[1:2^{nR^{\prime \prime }}\right]\right\}\;, \end{array}$$

for b∈[2:B−1]. The codebooks $F_{1}$ and $F_{B}$ are generated in the same manner, with fixed $m_{0}^{\prime }=m_{B}^{\prime }\equiv 1$ and $m_{0}^{\prime \prime }=m_{B}^{\prime \prime }\equiv 1$. Encoding and decoding is illustrated in Figure A1.

*Encoding*: To send the message sequence $\left(m_{1}^{\prime },m_{1}^{\prime \prime },\ldots ,m_{B-1}^{\prime },m_{B-1}^{\prime \prime }\right)$, transmit $x_{b}^{n}\left(m_{b}^{\prime },m_{b}^{\prime \prime }|m_{b-1}^{\prime }\right)$ at block *b*, for b∈[1:B].

*Relay Encoding*: In block 1, the relay transmits $x_{1,1}^{n}\left(1\right)$. Set ${\tilde{m}}_{0}^{\prime }\equiv 1$. At the end of block b∈[1:B−1], the relay receives $y_{1,b}^{n}$, and finds some ${\tilde{m}}_{b}^{\prime }\in \left[1:2^{nR^{\prime }}\right]$ such that

(A8) $$\begin{array}{c} \left(u_{b}^{n}\left({\tilde{m}}_{b}^{\prime }|{\tilde{m}}_{b-1}^{\prime }\right),x_{1,b}^{n}\left({\tilde{m}}_{b-1}^{\prime }\right),y_{1,b}^{n}\right)\in A^{\delta }\left(P_{U,X_{1}}P_{Y_{1}|U,X_{1}}^{q}\right)\;,\;\mathrm{for}\;\mathrm{some}\;q\in {\hat{Q}}_{n}. \end{array}$$

If there is none or there is more than one such, set ${\tilde{m}}_{b}^{\prime }=1$. In block b+1, the relay transmits $x_{1,b+1}^{n}\left({\tilde{m}}_{b}^{\prime }\right)$.

*Backward Decoding*: Once all blocks ${\left(y_{b}^{n}\right)}_{b=1}^{B}$ are received, decoding is performed backwards. Set ${\hat{m}}_{B}^{\prime }={\hat{m}}_{B}^{\prime \prime }\equiv 1$. For b=B−1,B−2,…,1, find a unique ${\hat{m}}_{b}^{\prime }\in \left[1:2^{nR^{\prime }}\right]$ such that

(A9) $$\begin{array}{c} \left(u_{b+1}^{n}\left({\hat{m}}_{b+1}^{\prime }|{\hat{m}}_{b}^{\prime }\right),x_{1,b+1}^{n}\left({\hat{m}}_{b}^{\prime }\right),y_{b+1}^{n}\right)\in A^{\delta }\left(P_{U,X_{1}}P_{Y|U,X_{1}}^{q}\right)\;,\;\mathrm{for}\;\mathrm{some}\;q\in {\hat{Q}}_{n}. \end{array}$$

If there is none, or more than one such ${\hat{m}}_{b}^{\prime }\in \left[1:2^{nR^{\prime }}\right]$, declare an error.

Then, the decoder uses ${\hat{m}}_{1}^{\prime },\ldots ,{\hat{m}}_{B-1}^{\prime }$ as follows. For b=B−1,B−2,…,1, find a unique ${\hat{m}}_{b}^{\prime \prime }\in \left[1:2^{nR^{\prime \prime }}\right]$ such that

(A10) $$\begin{array}{c} \left(u_{b}^{n}\left({\hat{m}}_{b}^{\prime }|{\hat{m}}_{b-1}^{\prime }\right),x_{b}^{n}\left({\hat{m}}_{b}^{\prime },{\hat{m}}_{b}^{\prime \prime }|{\hat{m}}_{b-1}^{\prime }\right),x_{1,b}\left({\hat{m}}_{b-1}^{\prime }\right),y_{b}^{n}\right)\in A^{\delta }\left(P_{U,X,X_{1}}P_{Y|X,X_{1}}^{q}\right)\;,\;\mathrm{for}\;\mathrm{some}\;q\in {\hat{Q}}_{n}. \end{array}$$

If there is none, or more than one such ${\hat{m}}_{b}^{\prime \prime }\in \left[1:2^{nR^{\prime \prime }}\right]$, declare an error. We note that using the set of types ${\hat{Q}}_{n}$ instead of the original set of state distributions Q alleviates the analysis, since Q is not necessarily finite nor countable.

*Analysis of Probability of Error*: Assume without loss of generality that the user sent $\left(M_{b}^{\prime },M_{b}^{\prime \prime }\right)=\left(1,1\right)$, and let $q^{*}\left(s\right)\in Q$ denote the *actual* state distribution chosen by the jammer. The error event is bounded by the union of the events

(A11) $$\begin{array}{cc} E_{1}\left(b\right)= & \left\{{\tilde{M}}_{b}^{\prime }\neq 1\right\}\;,\;E_{2}\left(b\right)=\left\{{\hat{M}}_{b}^{\prime }\neq 1\right\}\;,\;E_{3}\left(b\right)=\left\{{\hat{M}}_{b}^{\prime \prime }\neq 1\right\}\;,\;\mathrm{for}\;b\in [1:B-1]. \end{array}$$

Then, the probability of error is bounded by

(A12) $$\begin{array}{cc} P_{e}^{\left(n\right)}\left(q,C\right)\leq & \sum_{b=1}^{B-1}\mathrm{Pr}\left(E_{1}\left(b\right)\right)+\sum_{b=1}^{B-1}\mathrm{Pr}\left(E_{2}\left(b\right)|E_{1}^{c}\left(b\right)\right)+\sum_{b=1}^{B-1}\mathrm{Pr}\left(E_{3}\left(b\right)|E_{1}^{c}\left(b\right)\cap E_{2}^{c}\left(b\right)\cap E_{2}^{c}\left(b-1\right)\right)\;, \end{array}$$

with $E_{2}\left(0\right)=\emptyset$, where the conditioning on $\left(M_{b}^{\prime },M_{b}^{\prime \prime }\right)=\left(1,1\right)$ is omitted for convenience of notation.

We begin with the probability of erroneous relaying, $\mathrm{Pr}\left(E_{1}\left(b\right)\right)$. Define

(A13) $$\begin{array}{cc} E_{1,1}\left(b\right)= & \left\{\left(U_{b}^{n}\left(1|{\tilde{M}}_{b-1}^{\prime }\right),X_{1,b}^{n}\left({\tilde{M}}_{b-1}^{\prime }\right),Y_{1,b}^{n}\right)\notin A^{\delta }\left(P_{U,X_{1}}P_{Y_{1}|U,X_{1}}^{q^{\prime }}\right)\;\;\mathrm{for}\;\mathrm{all}\;q^{\prime }\in {\hat{Q}}_{n}\right\}\\ E_{1,2}\left(b\right)= & \{\left(U_{b}^{n}\left(m_{b}^{\prime }|{\tilde{M}}_{b-1}^{\prime }\right),X_{1,b}^{n}\left({\tilde{M}}_{b-1}^{\prime }\right),Y_{1,b}^{n}\right)\in A^{\delta }\left(P_{U,X_{1}}P_{Y_{1}|U,X_{1}}^{q^{\prime }}\right)\;,\;\mathrm{for}\;\mathrm{some}\;m_{b}^{\prime }\neq 1,\;q^{\prime }\in {\hat{Q}}_{n}\}. \end{array}$$

For b∈[1:B−1], the relay error event is bounded as

(A14) $$\begin{array}{cc} E_{1}\left(b\right)\subseteq & E_{1}\left(b-1\right)\cup E_{1,1}\left(b\right)\cup E_{1,2}\left(b\right)\\ = & E_{1}\left(b-1\right)\cup \left(E_{1}{\left(b-1\right)}^{c}\cap E_{1,1}\left(b\right)\right)\cup \left(E_{1}{\left(b-1\right)}^{c}\cap E_{1,2}\left(b\right)\right)\;, \end{array}$$

with $E_{1}\left(0\right)=\emptyset$. Thus, by the union of events bound,

(A15) $$\begin{array}{c} \mathrm{Pr}\left(E_{1}\left(b\right)\right)\leq \mathrm{Pr}\left(E_{1}\left(b-1\right)\right)+\mathrm{Pr}\left(E_{1,1}\left(b\right)|E_{1}{\left(b-1\right)}^{c}\right)+\mathrm{Pr}\left(E_{1,2}\left(b\right)|E_{1}{\left(b-1\right)}^{c}\right). \end{array}$$

Consider the second term on the RHS of (A15). We now claim that given that $E_{1}{\left(b-1\right)}^{c}$ occurred, i.e., ${\tilde{M}}_{b-1}^{\prime }=1$, the event $E_{1,1}\left(b\right)$ implies that $\left(U_{b}^{n}\left(1|1\right),X_{1,b}^{n}\left(1\right),Y_{1,b}^{n}\right)\notin A^{\delta \;/\;2}\left(P_{U,X_{1}}P_{Y_{1}|U,X_{1}}^{q^{\prime \prime }}\right)$ for all $q^{\prime \prime }\in Q$. This claim is due to the following. Assume to the contrary that $E_{1,1}\left(b\right)$ holds, but $\left(U_{b}^{n}\left(1|1\right),X_{1,b}^{n}\left(1\right),Y_{1,b}^{n}\right)\in A^{\delta \;/\;2}\left(P_{U,X_{1}}P_{Y_{1}|U,X_{1}}^{q^{\prime \prime }}\right)$ for some $q^{\prime \prime }\in Q$. Then, for a sufficiently large *n*, there exists a type $q^{\prime }\left(s\right)$ such that

(A16) $$\begin{array}{c} |q^{\prime }\left(s\right)-q^{\prime \prime }\left(s\right)|\leq \delta_{1}\;, \end{array}$$

for all s∈S, and by the definition in (A1), $q^{\prime }\in {\hat{Q}}_{n}$. Then, (A16) implies that

(A17) $$\begin{array}{c} |P_{Y_{1}|U,X_{1}}^{q^{\prime }}\left(y_{1}|u,x_{1}\right)-P_{Y_{1}|U,X_{1}}^{q^{\prime \prime }}\left(y_{1}|u,x_{1}\right)|\leq |S|\cdot \delta_{1}=\frac{\delta }{2}\;, \end{array}$$

for all u∈U, $x_{1}\in X_{1}$ and $y_{1}\in Y_{1}$ (see (A4) and (A2)). Hence, $\left(U_{b}^{n}\left(1|1\right),X_{1,b}^{n}\left(1\right),Y_{1,b}^{n}\right)\in A^{\delta }\left(P_{U,X_{1}}P_{Y_{1}|U,X_{1}}^{q^{\prime }}\right)$, which contradicts the first assumption. It follows that

(A18) $$\begin{array}{cc} & \mathrm{Pr}\left(E_{1,1}\left(b\right)|E_{1}{\left(b-1\right)}^{c}\right)\\ \leq & \mathrm{Pr}\left(\left(U_{b}^{n}\left(1|1\right),X_{1,b}^{n}\left(1\right),Y_{1,b}^{n}\right)\notin A^{\delta \;/\;2}\left(P_{U,X_{1}}P_{Y_{1}|U,X_{1}}^{q^{\prime \prime }}\right)\;\;\mathrm{for}\;\mathrm{all}\;q^{\prime \prime }\in Q|E_{1}{\left(b-1\right)}^{c}\right)\\ \leq & \mathrm{Pr}\left(\left(U_{b}^{n}\left(1|1\right),X_{1,b}^{n}\left(1\right),Y_{1,b}^{n}\right)\notin A^{\delta \;/\;2}\left(P_{U,X_{1}}P_{Y_{1}|U,X_{1}}^{q^{*}}\right)|E_{1}{\left(b-1\right)}^{c}\right). \end{array}$$

Since the codebooks $F_{1},\ldots ,F_{B}$ are independent, the sequence $\left(U_{b}^{n}\left(1|1\right),X_{1,b}^{n}\left(1\right)\right)$ from the codebook $F_{b}$ is independent of the relay estimate ${\tilde{M}}_{b-1}$, which is a function of $Y_{1,b-1}^{n}$ and the codebook $F_{b-1}$. Thus, the RHS of (A18) tends to zero exponentially as n→∞ by the law of large numbers and Chernoff’s bound.

We move to the third term in the RHS of (A15). By the union of events bound, the fact that the number of type classes in $S^{n}$ is bounded by ${\left(n+1\right)}^{\left|S\right|}$, and the independence of the codebooks, we have that

(A19) $$\begin{array}{cc} & \mathrm{Pr}\left(E_{1,2}\left(b\right)|E_{1}{\left(b-1\right)}^{c}\right)\\ \leq & {\left(n+1\right)}^{\left|S\right|}\cdot \underset{q^{\prime }\in {\hat{Q}}_{n}}{\sup}\mathrm{Pr}\left(\left(U_{b}^{n}\left(m_{b}^{\prime }|1\right),X_{1,b}^{n}\left(1\right),Y_{1,b}^{n}\right)\in A^{\delta }\left(P_{U,X_{1}}P_{Y_{1}|U,X_{1}}^{q^{\prime }}\right)\;\;\mathrm{for}\;\mathrm{some}\;m_{b}^{\prime }\neq 1\right)\\ \leq & {\left(n+1\right)}^{\left|S\right|}\cdot 2^{nR^{\prime }}\cdot \underset{q^{\prime }\in {\hat{Q}}_{n}}{\sup}\left[\sum_{u^{n},x_{1}^{n}}P_{U^{n},X_{1}^{n}}\left(u^{n},x_{1}^{n}\right)\cdot \sum_{y_{1}^{n}\;:\;\left(u^{n},x_{1}^{n},y_{1}^{n}\right)\in A^{\delta }\left(P_{U,X_{1}}P_{Y_{1}|U,X_{1}}^{q^{\prime }}\right)}P_{Y_{1}^{n}|X_{1}^{n}}^{q^{*}}\left(y_{1}^{n}|x_{1}^{n}\right)\right]\;, \end{array}$$

where the last line follows since $U_{b}^{n}\left(m_{b}^{\prime }|1\right)$ is conditionally independent of $Y_{1,b}^{n}$ given $X_{1,b}^{n}\left(1\right)$, for every $m_{b}^{\prime }\neq 1$. Let $y_{1}^{n}$ satisfy $\left(u^{n},x_{1}^{n},y_{1}^{n}\right)\in A^{\delta }\left(P_{U,X_{1}}P_{Y_{1}|U,X_{1}}^{q^{\prime }}\right)$. Then, $\;\left(x_{1}^{n},y_{1}^{n}\right)\in A^{\delta_{2}}\left(P_{X_{1},Y_{1}}^{q^{\prime }}\right)$ with $\delta_{2}≜\left|U\right|\cdot \delta$. By Lemmas 2.6 and 2.7 in [93],

$$\begin{array}{c} P_{X_{1}^{n},Y_{1}^{n}}^{q^{*}}\left(x_{1}^{n},y_{1}^{n}\right)=2^{-n\left(H\left({\hat{P}}_{x_{1}^{n},y_{1}^{n}}\right)+D({\hat{P}}_{x_{1}^{n},y_{1}^{n}}\left|\right|P_{X_{1},Y_{1}}^{q^{*}})\right)}\leq 2^{-nH({\hat{P}}_{x_{1}^{n},y_{1}^{n}})}\leq 2^{-n\left(H_{q^{\prime }}\left(X_{1},Y_{1}\right)-\varepsilon_{1}\left(\delta \right)\right)}\;, \end{array}$$

hence,

(A20) $$\begin{array}{c} P_{Y_{1}^{n}|X_{1}^{n}}^{q^{*}}\left(y_{1}^{n}|x_{1}^{n}\right)\leq 2^{-n\left(H_{q^{\prime }}\left(Y_{1}|X_{1}\right)-\varepsilon_{2}\left(\delta \right)\right)}\;, \end{array}$$

where $\varepsilon_{1}\left(\delta \right),\varepsilon_{2}\left(\delta \right)\to 0$ as δ→0. Therefore, by Equation (A19)−(A20), along with [93] (Lemma 2.13),

(A21) $$\begin{array}{c} \mathrm{Pr}\left(E_{1,2}\left(b\right)|E_{1}{\left(b-1\right)}^{c}\right)\leq \;{\left(n+1\right)}^{\left|S\right|}\cdot \underset{q^{\prime }\in Q}{\sup}2^{-n[I_{q^{\prime }}\left(U;Y_{1}|X_{1}\right)-R^{\prime }-\varepsilon_{3}\left(\delta \right)]}\;, \end{array}$$

with $\varepsilon_{3}\left(\delta \right)\to 0$ as δ→0. Using induction, we have by (A15) that $\mathrm{Pr}\left(E_{1}\left(b\right)\right)$ tends to zero exponentially as n→∞, for b∈[1:B−1], provided that $R^{\prime }<\inf_{q^{\prime }\in Q}I_{q^{\prime }}\left(U;Y_{1}|X_{1}\right)-\varepsilon_{3}\left(\delta \right)$.

As for the erroneous decoding of $M_{b}^{\prime }$ at the receiver, observe that given $E_{1}{\left(b\right)}^{c}$, the relay sends $X_{1,b}^{n}\left(1\right)$ in block b+1, hence

(A22) $$\begin{array}{c} \left(U_{b+1}^{n}\left(1|1\right),X_{b+1}^{n}\left(1,1|1\right),X_{1,b+1}^{n}\left(1\right)\right)∼P_{U,X,X_{1}}\left(u,x,x_{1}\right). \end{array}$$

At the destination receiver, decoding is performed backwards, hence the error events have a different form compared to those of the relay (cf. (A13) and the events below). Define the events,

(A23) $$\begin{array}{cc} E_{2,1}\left(b\right)= & \left\{\left(U_{b+1}^{n}\left({\hat{M}}_{b+1}^{\prime }|1\right),X_{1,b+1}^{n}\left(1\right),Y_{b+1}^{n}\right)\notin A^{\delta }\left(P_{U,X_{1}}P_{Y|U,X_{1}}^{q^{\prime }}\right)\;\;\mathrm{for}\;\mathrm{all}\;q^{\prime }\in {\hat{Q}}_{n}\right\}\\ E_{2,2}\left(b\right)= & \left\{\left(U_{b+1}^{n}\left({\hat{M}}_{b+1}^{\prime }|m_{b}^{\prime }\right),X_{1,b+1}^{n}\left(m_{b}^{\prime }\right),Y_{b+1}^{n}\right)\in A^{\delta }\left(P_{U,X_{1}}P_{Y_{1}|U,X_{1}}^{q^{\prime }}\right)\;,\;\mathrm{for}\;\mathrm{some}\;m_{b}^{\prime }\neq 1,\;q^{\prime }\in {\hat{Q}}_{n}\right\} \end{array}$$

For b∈[1:B−1], the error event $E_{2}\left(b\right)$ is bounded by

(A24) $$\begin{array}{cc} E_{2}\left(b\right)\subseteq & E_{2}\left(b+1\right)\cup E_{2,1}\left(b\right)\cup E_{2,2}\left(b\right)\\ = & E_{2}\left(b+1\right)\cup \left(E_{2}{\left(b+1\right)}^{c}\cap E_{2,1}\left(b\right)\right)\cup \left(E_{2}{\left(b+1\right)}^{c}\cap E_{2,2}\left(b\right)\right)\;, \end{array}$$

with $E_{2}\left(B\right)=\emptyset$. Thus,

(A25) $$\begin{array}{cc} \mathrm{Pr}\left(E_{2}\left(b\right)|E_{1}{\left(b\right)}^{c}\right)\leq & \mathrm{Pr}\left(E_{2}\left(b+1\right)|E_{1}{\left(b\right)}^{c}\right)+\mathrm{Pr}\left(E_{2,1}\left(b\right)|E_{1}{\left(b\right)}^{c},E_{2}{\left(b+1\right)}^{c}\right)\\ & +\mathrm{Pr}\left(E_{2,2}\left(b\right)|E_{1}{\left(b\right)}^{c},E_{2}{\left(b+1\right)}^{c}\right). \end{array}$$

By similar arguments to those used above, we have that

(A26) $$\begin{array}{c} \mathrm{Pr}\left(E_{2,1}\left(b\right)|E_{1}{\left(b\right)}^{c},E_{2}{\left(b+1\right)}^{c}\right)\leq \mathrm{Pr}\left(\left(U_{b+1}^{n}\left(1|1\right),X_{1,b+1}^{n}\left(1\right),Y_{b+1}^{n}\right)\notin A^{\delta \;/\;2}\left(P_{U,X_{1}}P_{Y|U,X_{1}}^{q^{*}}\right)|E_{1}{\left(b\right)}^{c}\right)\;, \end{array}$$

which tends to zero exponentially as n→∞, due to (A22), and by the law of large numbers and Chernoff’s bound. Then, by similar arguments to those used for the bound on $\mathrm{Pr}\left(E_{1,2}\left(b\right)|E_{1}{\left(b-1\right)}^{c}\right)$, the third term on the RHS of (A25) tends to zero as n→∞, provided that $R^{\prime }<\inf_{q^{\prime }\in Q}I_{q^{\prime }}\left(U,X_{1};Y\right)-\varepsilon_{4}\left(\delta \right)$, where $\varepsilon_{4}\left(\delta \right)\to 0$ as δ→0. Using induction, we have by (A25) that the second term on the RHS of (A12) tends to zero exponentially as n→∞, for b∈[1:B−1].

Moving to the error event for $M_{b}^{\prime \prime }$, define

(A27) $$\begin{array}{cc} E_{3,1}\left(b\right)= & \left\{\left(U_{b}^{n}\left({\hat{M}}_{b}^{\prime }|{\hat{M}}_{b-1}^{\prime }\right),X_{b}^{n}\left({\hat{M}}_{b}^{\prime },1|{\hat{M}}_{b-1}^{\prime }\right),X_{1,b}\left({\hat{M}}_{b-1}^{\prime }\right),Y_{b}^{n}\right)\notin A^{\delta }\left(P_{U,X,X_{1}}P_{Y|X,X_{1}}^{q^{\prime }}\right)\;,\;\mathrm{for}\;\mathrm{all}\;q^{\prime }\in {\hat{Q}}_{n}\right\}\\ E_{3,2}\left(b\right)= & \{\left(U_{b}^{n}\left({\hat{M}}_{b}^{\prime }|{\hat{M}}_{b-1}^{\prime }\right),X_{b}^{n}\left({\hat{M}}_{b}^{\prime },m_{b}^{\prime \prime }|{\hat{M}}_{b-1}^{\prime }\right),X_{1,b}\left({\hat{M}}_{b-1}^{\prime }\right),Y_{b}^{n}\right)\in A^{\delta }\left(P_{U,X,X_{1}}P_{Y|X,X_{1}}^{q^{\prime }}\right)\;,\;\\ & \mathrm{for}\;\mathrm{some}\;m_{b}^{\prime \prime }\neq 1,\;q^{\prime }\in {\hat{Q}}_{n}\}. \end{array}$$

Given $E_{2}{\left(b\right)}^{c}\cap E_{2}{\left(b-1\right)}^{c}$, we have that ${\hat{M}}_{b}^{\prime }=1$ and ${\hat{M}}_{b-1}^{\prime }=1$. Then, by similar arguments to those used above,

(A28) $$\begin{array}{cc} & \mathrm{Pr}\left(E_{3}\left(b\right)|E_{1}{\left(b\right)}^{c}\cap E_{2}{\left(b\right)}^{c}\cap E_{2}{\left(b-1\right)}^{c}\right)\\ \leq & \mathrm{Pr}\left(E_{3,1}\left(b\right)|E_{1}{\left(b\right)}^{c}\cap E_{2}{\left(b\right)}^{c}\cap E_{2}{\left(b-1\right)}^{c}\right)+\mathrm{Pr}\left(E_{3,2}\left(b\right)|E_{1}{\left(b\right)}^{c}\cap E_{2}{\left(b\right)}^{c}\cap E_{2}{\left(b-1\right)}^{c}\right)\\ \leq & e^{-a_{0}n}+{\left(n+1\right)}^{\left|S\right|}\cdot \underset{q^{\prime }\in Q}{\sup}\sum_{m_{b}^{\prime \prime }\neq 1}\mathrm{Pr}\left(\left(U_{b}^{n}\left(1|1\right),X_{b}^{n}\left(1,m_{b}^{\prime \prime }|1\right),X_{1,b}\left(1\right),Y_{b}^{n}\right)\in A^{\delta }\left(P_{U,X,X_{1}}P_{Y|X,X_{1}}^{q^{\prime }}\right)|E_{1}{\left(b\right)}^{c}\right)\\ \leq & e^{-a_{0}n}+{\left(n+1\right)}^{\left|S\right|}\cdot \underset{q^{\prime }\in Q}{\sup}2^{-n[I_{q^{\prime }}\left(X;Y|U,X_{1}\right)-R^{\prime \prime }-\varepsilon_{5}\left(\delta \right)]} \end{array}$$

where $a_{0}>0$ and $\varepsilon_{5}\left(\delta \right)\to 0$ as δ→0. The second inequality holds by (A22) along with the law of large numbers and Chernoff’s bound, and the last inequality holds as $X_{b}^{n}\left(1,m_{b}^{\prime \prime }|1\right)$ is conditionally independent of $Y_{b}^{n}$ given $\left(U_{b}^{n}\left(1|1\right),X_{1,b}^{n}\left(1\right)\right)$ for every $m_{b}^{\prime \prime }\neq 1$. Thus, the third term on the RHS of (A12) tends to zero exponentially as n→∞, provided that $R^{\prime \prime }<\inf_{q^{\prime }\in Q}I_{q^{\prime }}\left(X;Y|U,X_{1}\right)-\varepsilon_{5}\left(\delta \right)$. Eliminating $R^{\prime }$ and $R^{\prime \prime }$, we conclude that the probability of error, averaged over the class of the codebooks, exponentially decays to zero as n→∞, provided that $R<R_{PDF}\left(L^{Q}\right)$. Therefore, there must exist a $\left(2^{nR},n,\varepsilon \right)$ deterministic code, for a sufficiently large *n*. □

### Appendix A.2. Cutset Upper Bound

This is a straightforward consequence of the cutset bound in [4]. Assume to the contrary that there exists an achievable rate $R>R_{CS}\left(L^{Q}\right)$. Then, for some $q^{*}\left(s\right)$ in the closure of Q,

(A29) $$\begin{array}{c} R>\max_{p(x,x_{1})}\min\left\{I_{q^{*}}\left(X,X_{1};Y\right)\;,\;I_{q^{*}}\left(X;Y,Y_{1}|X_{1}\right)\right\}. \end{array}$$

By the achievability assumption, we have that for every ε>0 and sufficiently large *n*, there exists a $\left(2^{nR},n\right)$ random code $C^{\Gamma }$ such that $P_{e}^{\left(n\right)}\left(q,C\right)\leq \varepsilon$ for every i.i.d. state distribution q∈Q, and in particular for $q^{*}$. This holds even if $q^{*}$ is in the closure of Q but not in Q itself, since $P_{e}^{\left(n\right)}\left(q,C\right)$ is continuous in *q*. Consider using this code over a standard relay channel $W_{Y,Y_{1}|X,X_{1}}$ without a state, where $W_{Y,Y_{1}|X,X_{1}}\left(y,y_{1}|x,x_{1}\right)=\sum_{s\in S}q^{*}\left(s\right)W_{Y,Y_{1}|X,X_{1},S}\left(y,y_{1}|x,x_{1},s\right)$. It follows that the rate *R* as in (A29) can be achieved over the relay channel $W_{Y,Y_{1}|X,X_{1}}$, in contradiction to [4]. We deduce that the assumption is false, and $R>R_{CS}\left(L^{Q}\right)$ cannot be achieved. □

## Appendix B. Proof of Corollary 1

This is a straightforward consequence of Lemma 1, which states that the capacity of the compound relay channel is bounded by $R_{PDF}\left(L^{Q}\right)\leq C\left(L^{Q}\right)\leq R_{CS}\left(L^{Q}\right)$. Thus, if $W_{Y,Y_{1}|X,X_{1},S}$ is reversely degraded such that $W_{Y,Y_{1}|X,X_{1},S}=W_{Y|X,X_{1}}W_{Y_{1}|Y,X_{1},S}$, then $I_{q}\left(X;Y,Y_{1}|X_{1}\right)=I_{q}\left(X;Y|X_{1}\right)$, and the bounds coincide by the minimax theorem [90], cf. (8) and (12). Similarly, if $W_{Y,Y_{1}|X,X_{1},S}$ is strongly degraded, i.e., $W_{Y,Y_{1}|X,X_{1},S}=W_{Y_{1}|X,X_{1}}W_{Y|Y_{1},X_{1},S}$, then $I_{q}\left(X;Y,Y_{1}|X_{1}\right)=I\left(X;Y_{1}|X_{1}\right)$, and by (8) and (13),

(A30) $$\begin{array}{cc} R_{CS}\left(L^{Q}\right)= & \min_{q(s)\in Q}\max_{p(x,x_{1})}\min\left\{I_{q}\left(X,X_{1};Y\right)\;,\;I\left(X;Y_{1}|X_{1}\right)\right\}\;\;, \end{array}$$

(A31) $$\begin{array}{cc} R_{PDF}\left(L^{Q}\right)= & \max_{p(x,x_{1})}\min_{q(s)\in Q}\min\left\{I_{q}\left(X,X_{1};Y\right)\;,\;I\left(X;Y_{1}|X_{1}\right)\right\}\;. \end{array}$$

Observe that $\min\left\{I_{q}\left(X,X_{1};Y\right)\;,\;I\left(X;Y_{1}|X_{1}\right)\right\}$ is concave in $p(x,x_{1})$ and quasi-convex in q(s) (see e.g., [94] (Section 3.4])), hence the bounds (A30) and (A31) coincide by the minimax theorem [90]. □

## Appendix C. Proof of Corollary 2

Consider the block-compound relay channel $L^{Q\times B}$, where the state distribution $q_{b}\in Q$ varies from block to block. Since the encoder, relay and receiver are aware of this jamming scheme, they can use a block coding scheme that is synchronized with the jammer block strategy. Thus, the capacity is the same as that of the ordinary compound channel, i.e., $C\left(L^{Q\times B}\right)=C\left(L^{Q}\right)$ and $C^{⋆}\left(L^{Q\times B}\right)=C^{⋆}\left(L^{Q}\right)$. Hence, (17) and (18) follow from Lemma 1. As for the second part of Corollary 2, observe that the block Markov coding scheme used in the proof of the partial decode-forward lower bound can be applied as is to the block-compound relay channel, since the relay and the destination receiver do not estimate the state distribution while decoding the messages (see Appendix A). Furthermore, the analysis also holds, where the actual state distribution $q^{*}$, in (A18)–(A20) and (A26), is now replaced by the state distribution $q_{b}^{*}$ which corresponds to block b∈[1:B]. □

## Appendix D. Proof of Theorem 1

First, we explain the general idea. We modify Ahlswede’s Robustification Technique (RT) [59] to the relay channel. Namely, we use codes for the compound relay channel to construct a random code for the AVRC using randomized permutations. However, in our case, the strictly causal nature of the relay imposes a difficulty, and the application of the RT is not straightforward.

In [59], there is noncausal state information and a random code is defined via permutations of the codeword symbols and the received sequence. Here, however, the relay cannot apply permutations to its transmission $x_{1}^{n}$, because it depends on the received sequence $y_{1}^{n}$ in a strictly causal manner. We resolve this difficulty using block Markov codes for the block-compound relay channel to construct a random code for the AVRC, applying *B* in-block permutations to the relay transmission, which depends only on the sequence received in the *previous block*. The details are given below.

### Appendix D.1. Partial Decode-Forward Lower Bound

We show that every rate $R<R_{PDF}^{⋆}\left(L\right)$ (see (19)) can be achieved by random codes over the AVRC L, i.e., $C\left(L\right)\geq R_{PDF}^{⋆}\left(L\right)$. We start with Ahlswede’s RT [59], stated below. Let $h:S^{n}\to \left[0,1\right]$ be a given function. If, for some fixed $\alpha_{n}\in \left(0,1\right)$, and for all $q\left(s^{n}\right)=\prod_{i=1}^{n}q\left(s_{i}\right)$, with q∈P(S),

(A32) $$\begin{array}{c} \sum_{s^{n}\in S^{n}}q\left(s^{n}\right)h\left(s^{n}\right)\leq \alpha_{n}\;, \end{array}$$

then,

(A33) $$\begin{array}{c} \frac{1}{n!}\sum_{\pi \in \Pi_{n}}h\left(\pi s^{n}\right)\leq \beta_{n}\;,\;\mathrm{for}\;\mathrm{all}\;s^{n}\in S^{n}\;, \end{array}$$

where $\Pi_{n}$ is the set of all *n*-tuple permutations $\pi :S^{n}\to S^{n}$, and $\beta_{n}={\left(n+1\right)}^{\left|S\right|}\cdot \alpha_{n}$.

According to Corollary 2, for every $R<R_{PDF}^{⋆}\left(L\right)$, there exists a $(2^{nR(B-1)},$ nB, $e^{-2\theta n})$ block Markov code for the block-compound relay channel $L^{P(S)\times B}$ for some θ>0 and sufficiently large *n*, where B>0 is arbitrarily large. Recall that the code constructed in the proof in Appendix A has the following form. The encoders use B>0 blocks to convey B−1 messages $m_{b}$, b∈[1:B−1]. Each message consists of two parts, i.e., $m_{b}=\left(m_{b}^{\prime },m_{b}^{\prime \prime }\right)$, where $m_{b}^{\prime }\in \left[1:2^{nR^{\prime }}\right]$ and $m_{b}^{\prime \prime }\in \left[1:2^{nR^{\prime \prime }}\right]$. In block b∈[1:B], the encoder sends $x_{b}^{n}=f_{b}\left(m_{b}^{\prime },m_{b}^{\prime \prime }|m_{b-1}^{\prime }\right)$, with fixed $m_{0}$ and $m_{B}$, and the relay transmits $x_{1,b}^{n}=f_{1,b}\left(y_{1,b-1}^{n}\right)$, using the sequence received in the previous block. After receiving the entire output sequence ${\left(y_{b}^{n}\right)}_{b=1}^{B}$, the decoder finds an estimate for the messages. Set ${\hat{m}}_{B}^{\prime }=1$. The first part of each message is decoded backwards as ${\hat{m}}_{b}^{\prime }=g_{b}^{\prime }\left(y_{b+1}^{n},{\hat{m}}_{b+1}^{\prime }\right)$, for b=B−1,B−2,…,1. Then, the second part of each message is decoded as ${\hat{m}}_{b}^{\prime \prime }=g_{b}^{\prime \prime }\left(y_{b}^{n},{\hat{m}}_{1}^{\prime },\ldots ,{\hat{m}}_{B-1}^{\prime }\right)$, for b∈[1:B−1]. The overall blocklength is then n·B and the average rate is $\frac{B-1}{B}\left(R^{\prime }+R^{\prime \prime }\right)$.

Given such a block Markov code $C_{BM}$ for the block-compound relay channel $L^{P(S)\times B}$, we have that

(A34) $$\begin{array}{c} {\mathrm{Pr}}_{\;C_{BM}}\;\left(E_{b}^{\prime }\;|\;{\left(E_{b+1}^{\prime }\right)}^{c}\right)\leq e^{-2\theta n}\;,\;{\mathrm{Pr}}_{\;C_{BM}}\;\left(E_{b}^{\prime \prime }\;|\;E_{1}^{\prime c},\ldots ,E_{b-1}^{\prime c}\right)\leq e^{-2\theta n} \end{array}$$

for b=B−1,…,1, where $E_{0}^{\prime }=E_{B}^{\prime }=\emptyset$, and $E_{b}^{\prime }=\left\{{\hat{M}}_{b}^{\prime }\neq M_{b}^{\prime }\right\}$, $E_{b}^{\prime \prime }=\left\{{\hat{M}}_{b}^{\prime \prime }\neq M_{b}^{\prime \prime }\right\}$, b∈[1:B−1]. That is, for every sequence of state distributions $q_{1},\ldots ,q_{b+1}$, where $q_{t}\left(s_{t}^{n}\right)=\prod_{i=1}^{n}q_{t}\left(s_{t,i}\right)$ for t∈[1:b+1],

(A35) $$\begin{array}{c} \sum_{s_{1}^{n}\in S^{n}}q_{1}\left(s_{1}^{n}\right)\sum_{s_{2}^{n}\in S^{n}}q_{2}\left(s_{2}^{n}\right)\;\cdots \sum_{s_{b+1}^{n}\in S^{n}}q_{b+1}\left(s_{b+1}^{n}\right)\cdot h_{b}^{\prime }\left(s_{1}^{n},s_{2}^{n},\ldots ,s_{b+1}^{n}\right)\leq e^{-2\theta n}\;,\; \end{array}$$

and

(A36) $$\begin{array}{c} \sum_{s_{1}^{n}\in S^{n}}q_{1}\left(s_{1}^{n}\right)\sum_{s_{2}^{n}\in S^{n}}q_{2}\left(s_{2}^{n}\right)\;\cdots \sum_{s_{b}^{n}\in S^{n}}q_{b}\left(s_{b}^{n}\right)\cdot h_{b}^{\prime \prime }\left(s_{1}^{n},s_{2}^{n},\ldots ,s_{b}^{n}\right)\leq e^{-2\theta n}\;, \end{array}$$

where

(A37) $$\begin{array}{c} h_{b}^{\prime }\left(s_{1}^{n},s_{2}^{n},\ldots ,s_{b+1}^{n}\right)=\frac{1}{2^{n\left(b+1\right)\left(R^{\prime }+R^{\prime \prime }\right)}}\sum_{\left(m_{1}^{\prime },m_{1}^{\prime \prime }\right),\ldots ,\left(m_{b+1}^{\prime },m_{b+1}^{\prime \prime }\right)}\\ \sum_{y_{1,b}^{n}\in Y_{1}^{n}}\mathrm{Pr}\left(Y_{1,b}^{n}=y_{1,b}^{n}|\left(M_{1}^{\prime },M_{1}^{\prime \prime }\right)=\left(m_{1}^{\prime },m_{1}^{\prime \prime }\right),\ldots ,\left(M_{b}^{\prime },M_{b}^{\prime \prime }\right)=\left(m_{b}^{\prime },m_{b}^{\prime \prime }\right),S_{1}^{n}=s_{1}^{n},\ldots ,S_{b}^{n}=s_{b}^{n}\right)\\ \;\times \sum_{y_{b+1}^{n}:g_{b}^{\prime }\left(y_{b+1}^{n},m_{b+1}^{\prime }\right)\neq m_{b}^{\prime }}W_{Y^{n}|X^{n},X_{1}^{n},S^{n}}\left(y_{b+1}^{n}|f_{b+1}\left(m_{b+1}^{\prime },m_{b+1}^{\prime \prime }|m_{b}^{\prime }\right),f_{1,b+1}\left(y_{1,b}^{n}\right),s_{b+1}^{n}\right) \end{array}$$

and

(A38) $$\begin{array}{c} h_{b}^{\prime \prime }\left(s_{1}^{n},s_{2}^{n},\ldots ,s_{b}^{n}\right)=\frac{1}{2^{nR^{\prime \prime }}}\sum_{m_{b}^{\prime \prime }=1}^{2^{nR^{\prime \prime }}}\frac{1}{2^{nR^{\prime }\left(B-1\right)}}\sum_{m_{1}^{\prime },\ldots ,m_{B-1}^{\prime }}\\ \;\sum_{y_{1,b-1}^{n}\in Y_{1}^{n}}\mathrm{Pr}(Y_{1,b-1}^{n}=y_{1,b-1}^{n}|\left(M_{1}^{\prime },M_{1}^{\prime \prime }\right)=\left(m_{1}^{\prime },m_{1}^{\prime \prime }\right),\ldots ,\left(M_{b-1}^{\prime },M_{b-1}^{\prime \prime }\right)=\left(m_{b-1}^{\prime },m_{b-1}^{\prime \prime }\right),\\ S_{1}^{n}=s_{1}^{n},\ldots ,S_{b-1}^{n}=s_{b-1}^{n})\\ \;\times \sum_{y_{b}^{n},y_{1,b}^{n}:g_{b}^{\prime \prime }\left(y_{b}^{n},m_{1}^{\prime },\ldots ,m_{B-1}^{\prime }\right)\neq m_{b}^{\prime \prime }}W_{Y^{n}|X^{n},X_{1}^{n},S^{n}}\left(y_{b}^{n}|f_{b}\left(m_{b}^{\prime },m_{b}^{\prime \prime }|m_{b-1}^{\prime }\right),f_{1,b}\left(y_{1,b-1}^{n}\right),s_{b}^{n}\right). \end{array}$$

The conditioning in the equations above can be explained as follows. In (A37), due to the code construction, the sequence $Y_{1,b}^{n}$ received at the relay in block b∈[1:B] depends only on the messages $\left(M_{t}^{\prime },M_{t}^{\prime \prime }\right)$ with t≤b. The decoded message ${\hat{M}}_{b}^{\prime }$, at the destination receiver, depends on messages $M_{t}^{\prime }$ with t>b, since the receiver decodes this part of the message backwards. In (A38), since the second part of the message $M_{b}^{\prime \prime }$ is decoded after backward decoding is complete, the estimation of $M_{b}^{\prime \prime }$ at the decoder depends on the entire sequence ${\hat{M}}_{1}^{\prime },\ldots ,{\hat{M}}_{B-1}^{\prime }$. By (A35)–(A36), for every t∈[1:b], $h_{b}^{\prime }$ and $h_{b}^{\prime \prime }$ as functions of $s_{t+1}^{n}$ and $s_{t}^{n}$, respectively, satisfy (A32) with $\alpha_{n}=e^{-2\theta n}$, given that the state sequences in the other blocks are fixed. Hence, applying Ahlswede’s RT recursively, we obtain

(A39) $$\begin{array}{c} \frac{1}{{\left(n!\right)}^{b+1}}\sum_{\pi_{1},\pi_{2},\ldots ,\pi_{b+1}\in \Pi_{n}}h_{b}^{\prime }\left(\pi_{1}s_{1}^{n},\pi_{2}s_{2}^{n},\ldots ,\pi_{b+1}s_{b+1}^{n}\right)\leq {\left(n+1\right)}^{B|S|}e^{-2\theta n}\leq e^{-\theta n},\\ \frac{1}{{\left(n!\right)}^{b}}\sum_{\pi_{1},\pi_{2},\ldots ,\pi_{b}\in \Pi_{n}}h_{b}^{\prime \prime }\left(\pi_{1}s_{1}^{n},\pi_{2}s_{2}^{n},\ldots ,\pi_{b}s_{b}^{n}\right)\leq {\left(n+1\right)}^{B|S|}e^{-2\theta n}\leq e^{-\theta n}\;, \end{array}$$

for all $\left(s_{1}^{n},s_{2}^{n},\ldots ,s_{b+1}^{n}\right)\in S^{(b+1)n}$ and sufficiently large *n*, such that ${\left(n+1\right)}^{B|S|}\leq e^{\theta n}$.

On the other hand, for every $\pi_{1},\pi_{2},\ldots ,\pi_{b+1}\in \Pi_{n}$, we have that

(A40) $$\begin{array}{c} h_{b}^{\prime }\left(\pi_{1}s_{1}^{n},\pi_{2}s_{2}^{n},\ldots ,\pi_{b+1}s_{b+1}^{n}\right)=E\;h_{b}^{\prime }\left(\pi_{1}s_{1}^{n},\pi_{2}s_{2}^{n},\ldots ,\pi_{b+1}s_{b+1}^{n}|M_{t}^{\prime },M_{t}^{\prime \prime },t=1,\ldots ,b+1\right)\;, \end{array}$$

with

$$\begin{array}{cc} & h_{b}^{\prime }\left(\pi_{1}s_{1}^{n},\pi_{2}s_{2}^{n},\ldots ,\pi_{b+1}s_{b+1}^{n}|m_{t}^{\prime },m_{t}^{\prime \prime },t=1,\ldots ,b+1\right)\\ = & \sum_{y_{1,1},\ldots ,y_{1,b}}\prod_{t=0}^{b-1}W_{Y_{1}^{n}|X^{n},X_{1}^{n},S^{n}}\left(y_{1,t+1}^{n}|f_{t+1}\left(m_{t+1}^{\prime },m_{t+1}^{\prime \prime }|m_{t}^{\prime }\right),f_{1,t+1}\left(y_{1,t}^{n}\right),\pi_{t+1}s_{t+1}^{n}\right)\\ & \times \sum_{y_{b+1}^{n}:g_{b}^{\prime }\left(y_{b+1}^{n},m_{b+1}^{\prime }\right)\neq m_{b}^{\prime }}W_{Y^{n}|X^{n},X_{1}^{n},S^{n}}\left(y_{b+1}^{n}|f_{b+1}\left(m_{b+1}^{\prime },m_{b+1}^{\prime \prime }|m_{b}^{\prime }\right),f_{1,b+1}\left(y_{1,b}^{n}\right),\pi_{b+1}s_{b+1}^{n}\right)\\ \overset{\left(a\right)}{=} & \sum_{y_{1,1},\ldots ,y_{1,b}}\prod_{t=0}^{b-1}W_{Y_{1}^{n}|X^{n},X_{1}^{n},S^{n}}\left(\pi_{t+1}y_{1,t+1}^{n}|f_{t+1}\left(m_{t+1}^{\prime },m_{t+1}^{\prime \prime }|m_{t}^{\prime }\right),f_{1,b+1}\left(\pi_{t}y_{1,t}^{n}\right),\pi_{t+1}s_{t+1}^{n}\right) \end{array}$$

(A41) $$\begin{array}{cc} & \times \sum_{y_{b+1}^{n}:g_{b}^{\prime }\left(\pi_{b+1}y_{b+1}^{n},m_{b+1}^{\prime }\right)\neq m_{b}^{\prime }}W_{Y^{n}|X^{n},X_{1}^{n},S^{n}}\left(\pi_{b+1}y_{b+1}^{n}|f_{b+1}\left(m_{b+1}^{\prime },m_{b+1}^{\prime \prime }|m_{b}^{\prime }\right),f_{1,b+1}\left(\pi_{b}y_{1,b}^{n}\right),\pi_{b+1}s_{b+1}^{n}\right)\\ \overset{\left(b\right)}{=} & \sum_{y_{1,1},\ldots ,y_{1,b}}\prod_{t=0}^{b-1}W_{Y_{1}^{n}|X^{n},X_{1}^{n},S^{n}}\left(y_{1,t+1}^{n}|\pi_{t+1}^{-1}f_{t+1}\left(m_{t+1}^{\prime },m_{t+1}^{\prime \prime }|m_{t}^{\prime }\right),\pi_{t+1}^{-1}f_{1,b+1}\left(\pi_{t}y_{1,t}^{n}\right),s_{t+1}^{n}\right)\\ & \times \sum_{y_{b+1}^{n}:g_{b}^{\prime }\left(\pi_{b+1}y_{b+1}^{n},m_{b+1}^{\prime }\right)\neq m_{b}^{\prime }}W_{Y^{n}|X^{n},X_{1}^{n},S^{n}}\left(y_{b+1}^{n}|\pi_{b+1}^{-1}f_{b+1}\left(m_{b+1}^{\prime },m_{b+1}^{\prime \prime }|m_{b}^{\prime }\right),\pi_{b+1}^{-1}f_{1,b+1}\left(\pi_{b}y_{1,b}^{n}\right),s_{b+1}^{n}\right)\;, \end{array}$$

where (a) is obtained by changing the order of summation over $y_{1,1}^{n},\ldots ,y_{1,b}^{n}$ and $y_{b+1}^{n}$; and (b) holds because the relay channel is memoryless. Similarly,

(A42) $$\begin{array}{c} h_{b}^{\prime \prime }\left(\pi_{1}s_{1}^{n},\pi_{2}s_{2}^{n},\ldots ,\pi_{b}s_{b}^{n}\right)=Eh_{b}^{\prime \prime }\left(\pi_{1}s_{1}^{n},\pi_{2}s_{2}^{n},\ldots ,\pi_{b}s_{b}^{n}|M_{1}^{\prime },\ldots ,M_{B-1}^{\prime },M_{t}^{\prime \prime },t=1,\ldots ,b\right)\;, \end{array}$$

with

(A43) $$\begin{array}{cc} & h_{b}^{\prime \prime }\left(\pi_{1}s_{1}^{n},\pi_{2}s_{2}^{n},\ldots ,\pi_{b}s_{b}^{n}|m_{1}^{\prime },\ldots ,m_{B-1}^{\prime },m_{t}^{\prime \prime },t=1,\ldots ,b\right)\\ = & \sum_{y_{1,1},\ldots ,y_{1,b-1}}\prod_{t=1}^{b-1}W_{Y_{1}^{n}|X^{n},X_{1}^{n},S^{n}}\left(y_{1,t}^{n}|f_{t}\left(m_{t}^{\prime },m_{t}^{\prime \prime }|m_{t-1}^{\prime }\right),f_{1,t}\left(y_{1,t}^{n}\right),\pi_{t}s_{t}^{n}\right)\\ & \times \sum_{y_{b}^{n}:g_{b}^{\prime \prime }\left(y_{b}^{n},m_{1}^{\prime },\ldots ,m_{B-1}^{\prime }\right)\neq m_{b}^{\prime \prime }}W_{Y^{n}|X^{n},X_{1}^{n},S^{n}}\left(y_{b}^{n}|f_{b}\left(m_{b}^{\prime },m_{b}^{\prime \prime }|m_{b-1}^{\prime }\right),f_{1,b}\left(y_{1,b-1}^{n}\right),\pi_{b}s_{b}^{n}\right)\\ \overset{\left(a\right)}{=} & \sum_{y_{1,1},\ldots ,y_{1,b-1}}\prod_{t=1}^{b-1}W_{Y_{1}^{n}|X^{n},X_{1}^{n},S^{n}}\left(\pi_{t}y_{1,t}^{n}|f_{t}\left(m_{t}^{\prime },m_{t}^{\prime \prime }|m_{t-1}^{\prime }\right),f_{1,t}\left(\pi_{t-1}y_{1,t-1}^{n}\right),\pi_{t}s_{t}^{n}\right)\\ & \times \sum_{y_{b}^{n}:g_{b}^{\prime \prime }\left(\pi_{b}y_{b}^{n},m_{1}^{\prime },\ldots ,m_{B-1}^{\prime }\right)\neq m_{b}^{\prime \prime }}W_{Y^{n}|X^{n},X_{1}^{n},S^{n}}\left(\pi_{b}y_{b}^{n}|f_{b}\left(m_{b}^{\prime },m_{b}^{\prime \prime }|m_{b-1}^{\prime }\right),f_{1,b}\left(\pi_{b-1}y_{1,b-1}^{n}\right),\pi_{b}s_{b}^{n}\right)\\ \overset{\left(b\right)}{=} & \sum_{y_{1,1},\ldots ,y_{1,b-1}}\prod_{t=1}^{b-1}W_{Y_{1}^{n}|X^{n},X_{1}^{n},S^{n}}\left(y_{1,t}^{n}|\pi_{t}^{-1}f_{t}\left(m_{t}^{\prime },m_{t}^{\prime \prime }|m_{t-1}^{\prime }\right),\pi_{t}^{-1}f_{1,t}\left(\pi_{t-1}y_{1,t-1}^{n}\right),s_{t}^{n}\right)\\ & \times \sum_{y_{b}^{n}:g_{b}^{\prime \prime }\left(\pi_{b}y_{b}^{n},m_{1}^{\prime },\ldots ,m_{B-1}^{\prime }\right)\neq m_{b}^{\prime \prime }}W_{Y^{n}|X^{n},X_{1}^{n},S^{n}}\left(y_{b}^{n}|\pi_{b}^{-1}f_{b}\left(m_{b}^{\prime },m_{b}^{\prime \prime }|m_{b-1}^{\prime }\right),\pi_{b}^{-1}f_{1,b}\left(\pi_{b-1}y_{1,b-1}^{n}\right),s_{b}^{n}\right). \end{array}$$

Then, consider the $\left(2^{nR(B-1)},nB\right)$ random Markov block code $C_{BM}^{\Pi }$, specified by

(A44a) $$\begin{array}{c} f_{b,\pi }\left(m_{b}^{\prime },m_{b}^{\prime \prime }|m_{b-1}^{\prime }\right)=\pi_{b}^{-1}f_{b}\left(m_{b}^{\prime },m_{b}^{\prime \prime }|m_{b-1}^{\prime }\right)\;,\;f_{1,b,\pi }\left(y_{1,b-1}^{n}\right)=\pi_{b}^{-1}f_{1,b}\left(\pi_{b-1}y_{1,b-1}^{n}\right)\;, \end{array}$$

and

(A44b) $$\begin{array}{c} g_{b,\pi }^{\prime }\left(y_{b+1}^{n},{\hat{m}}_{b+1}^{\prime }\right)=g_{b}^{\prime }\left(\pi_{b+1}y_{b+1}^{n},{\hat{m}}_{b+1}^{\prime }\right)\;,\;g_{b,\pi }^{\prime \prime }\left(y_{b}^{n},{\hat{m}}_{1}^{\prime },\ldots ,{\hat{m}}_{B-1}^{\prime }\right)=g_{b}^{\prime \prime }\left(\pi y_{b}^{n},{\hat{m}}_{1}^{\prime },\ldots ,{\hat{m}}_{B-1}^{\prime }\right)\;, \end{array}$$

for $\pi_{1},\ldots ,\pi_{B}\in \Pi_{n}$, with a uniform distribution $\mu \left(\pi_{1},\ldots ,\pi_{B}\right)=\frac{1}{|\Pi_{n}{|}^{B}}=\frac{1}{{\left(n!\right)}^{B}}$. That is, a set of *B* independent permutations is chosen at random and applied to all blocks simultaneously, while the order of the blocks remains intact. As we restricted ourselves to a block Markov code, the relaying function in a given block depends only on symbols received in the previous block, hence, the relay can implement those in-block permutations, and the coding scheme does not violate the causality requirement.

From (A41) and (A43), we see that using the random code $C_{BM}^{\Pi }$, the error probabilities for the messages $M_{b}^{\prime }$ and $M_{b}^{\prime \prime }$ are given by

(A45) $$\begin{array}{c} {\mathrm{Pr}}_{\;C_{BM}^{\Pi }}\;\left(E_{b}^{\prime }\;|\;{\left(E_{b+1}^{\prime }\right)}^{c},S_{1}^{n}=s_{1}^{n},\ldots ,S_{b+1}^{n}=s_{b+1}^{n}\right)=\sum_{\pi_{1},\ldots ,\pi_{B}\in \Pi_{n}}\mu \left(\pi_{1},\ldots ,\pi_{B}\right)h_{b}^{\prime }\left(\pi_{1}s_{1}^{n},\pi_{2}s_{2}^{n},\ldots ,\pi_{b+1}s_{b+1}^{n}\right)\;,\\ {\mathrm{Pr}}_{\;C_{BM}^{\Pi }}\;\left(E_{b}^{\prime \prime }\;|\;E_{1}^{\prime c},\ldots ,E_{B-1}^{\prime c},S_{1}^{n}=s_{1}^{n},\ldots ,S_{b}^{n}=s_{b}^{n}\right)=\sum_{\pi_{1},\ldots ,\pi_{B}\in \Pi_{n}}\mu \left(\pi_{1},\ldots ,\pi_{B}\right)h_{b}^{\prime \prime }\left(\pi_{1}s_{1}^{n},\pi_{2}s_{2}^{n},\ldots ,\pi_{b}s_{b}^{n}\right)\;, \end{array}$$

for all $s_{1}^{n},\ldots ,s_{b+1}^{n}\in S^{n}$, b∈[1:B−1], and therefore, together with (A39), we have that the probability of error of the random code $C_{BM}^{\Pi }$ is bounded by $P_{e}^{\left(n\right)}\left(q,C_{BM}^{\Pi }\right)\leq e^{-\theta n}$, for every $q\left(s^{nB}\right)\in P\left(S^{nB}\right)$. That is, $C_{BM}^{\Pi }$ is a $\left(2^{nR(B-1)},nB,e^{-\theta n}\right)$ random code for the AVRC L, where the overall blocklength is nB, and the average rate $\frac{B-1}{B}\cdot R$ tends to *R* as B→∞. This completes the proof of the partial decode-forward lower bound.

### Appendix D.2. Cutset Upper Bound

The proof immediately follows from Lemma 1, since the random code capacity of the AVRC is bounded by the random code capacity of the compound relay channel, i.e., $C^{⋆}\left(L\right)\leq C^{⋆}\left(L^{P(S)}\right)$. □

## Appendix E. Proof of Lemma 2

We use the approach of [55], with the required adjustments. We use the random code constructed in the proof of Theorem 1. Let $R<C^{⋆}\left(L\right)$, and consider the case where the marginal sender-relay and sender-receiver AVCs have positive capacity, i.e.,

(A46) $$\begin{array}{c} C\left(W_{1}\left(x_{1,1}\right)\right)>0\;,\;\mathrm{and}\;C\left(W\left(x_{1,2}\right)\right)>0\;, \end{array}$$

for some $x_{1,1},x_{1,2}\in X_{1}$ (see (23)). By Theorem 1, for every ε>0 and sufficiently large *n*, there exists a $\left(2^{nR},n,\varepsilon \right)$ random code $C^{\Gamma }=\left(\mu \left(\gamma \right)=\frac{1}{k},\Gamma =\left[1:k\right],{\left\{C_{\gamma }\right\}}_{\gamma \in \Gamma }\right)$, where $C_{\gamma }=\left(f_{\gamma }^{n},f_{1,\gamma },g_{\gamma }\right)$, for γ∈Γ. Following Ahlswede’s Elimination Technique [55], it can be assumed that the size of the code collection is bounded by $k=\left|\Gamma \right|\leq n^{2}$. By (A46), we have that for every $\varepsilon^{\prime }>0$ and sufficiently large $\nu^{\prime }$, the code index γ∈[1:k] can be sent through the relay channel $W_{Y_{1}|X,X_{1},S}$ using a $\left(2^{\nu^{\prime }{\tilde{R}}^{\prime }},\nu^{\prime },\varepsilon^{\prime }\right)$ deterministic code $C_{i}^{\prime }=\left({\tilde{f}}^{\nu^{\prime }},{\tilde{g}}^{\prime }\right)$, where ${\tilde{R}}^{\prime }>0$, while the relay repeatedly transmits the symbol $x_{1,1}$. Since *k* is at most polynomial, the encoder can reliably convey γ to the relay with a negligible blocklength, i.e., $\nu^{\prime }=o\left(n\right)$. Similarly, there exists $\left(2^{\nu^{\prime \prime }{\tilde{R}}^{\prime \prime }},\nu^{\prime \prime },\varepsilon^{\prime \prime }\right)$ code $C_{i}^{\prime \prime }=\left({\tilde{f}}^{\nu^{\prime \prime }},{\tilde{g}}^{\prime \prime }\right)$ for the transmission of γ∈[1:k] through the channel $W_{Y|X,X_{1},S}$ to the receiver, where $\nu^{\prime \prime }=o\left(n\right)$ and ${\tilde{R}}^{\prime \prime }>0$, while the relay repeatedly transmits the symbol $x_{1,2}$.

Now, consider a code formed by the concatenation of $C_{i}^{\prime }$ and $C_{i}^{\prime \prime }$ as consecutive prefixes to a corresponding code in the code collection ${\left\{C_{\gamma }\right\}}_{\gamma \in \Gamma }$. That is, the encoder first sends the index γ to the relay and the receiver, and then it sends the message $m\in [1:2^{nR}]$ to the receiver. Specifically, the encoder first transmits the $\left(\nu^{\prime }+\nu^{\prime \prime }\right)$-sequence $\left({\tilde{f}}^{\nu^{\prime }}\left(\gamma \right),{\tilde{f}}^{\nu^{\prime \prime }}\left(\gamma \right)\right)$ to convey the index γ, while the relay transmits the $\left(\nu^{\prime }+\nu^{\prime \prime }\right)$-sequence $\left({\tilde{x}}_{1}^{\nu^{\prime }},{\tilde{x}}_{1}^{\nu^{\prime \prime }}\right)$, where ${\tilde{x}}_{1}^{\nu^{\prime }}=\left(x_{1,1},x_{1,1},\ldots ,x_{1,1}\right)$ and ${\tilde{x}}_{1}^{\nu^{\prime \prime }}=\left(x_{1,2},x_{1,2},\ldots ,x_{1,2}\right)$. At the end of this transmission, the relay uses the first $\nu^{\prime }$ symbols it received to estimate the code index as ${\hat{\gamma }}^{\prime }={\tilde{g}}^{\prime }\left({\tilde{y}}_{1}^{\nu^{\prime }}\right)$.

Then, the message *m* is transmitted by the codeword $x^{n}=f_{\gamma }\left(m\right)$, while the relay transmits $x_{1}^{n}=f_{1,{\hat{\gamma }}^{\prime }}^{n}\left(y_{1}^{n}\right)$. Subsequently, decoding is performed in two stages as well; the decoder estimates the index at first, with ${\hat{\gamma }}^{\prime \prime }=$ ${\tilde{g}}^{\prime \prime }\left({\tilde{y}}^{\nu^{\prime \prime }}\right)$, and the message is then estimated by $\hat{m}=$ $g_{{\hat{\gamma }}^{\prime \prime }}\left(y^{n}\right)$. By the union of events bound, the probability of error is then bounded by $\varepsilon_{c}=\varepsilon +\varepsilon^{\prime }+\varepsilon^{\prime \prime }$, for every joint distribution in $P(S^{\nu^{\prime }+\nu^{\prime \prime }+n})$. That is, the concatenated code is a $\left(2^{\left(\nu^{\prime }+\nu^{\prime \prime }+n\right){\tilde{R}}_{n}},\nu^{\prime }+\nu^{\prime \prime }+n,\varepsilon_{c}\right)$ code over the AVRC L, where the blocklength is n+o(n), and the rate ${\tilde{R}}_{n}=\frac{n}{\nu^{\prime }+\nu^{\prime \prime }+n}\cdot R$ approaches *R* as n→∞. □

## Appendix F. Proof of Corollary 4

Consider part 1. By Definition 3, if $W_{Y_{1}|X,X_{1},S}$ and $W_{Y|X,X_{1},S}$ are not symmetrizable-$X|X_{1}$ then there exist $x_{1,1},x_{1,2}\in X_{1}$ such that the DMCs $W_{Y_{1}|X,X_{1},S}\left(\cdot \right|\cdot ,$ $x_{1,1},$ ·) and $W_{Y|X,X_{1},S}\left(\cdot |\cdot ,x_{1,2},\cdot \right)$ are non-symmetrizable in the sense of [57] (Definition 2). This, in turn, implies that $C(W_{1}\left(x_{1,1}\right))>0$ and $C(W\left(x_{1,2}\right))>0$, due to [57] (Theorem 1). Hence, by Lemma 2, $C\left(L\right)=C^{⋆}\left(L\right)$, and by Theorem 1, $R_{PDF}^{⋆}\left(L\right)\leq C\left(L\right)\leq R_{CS}^{⋆}\left(L\right)$.

Part 3 immediately follows from part 1 and Corollary 3. As for part 2, consider a strongly reversely degraded relay channel. We claim that if $W_{Y|X,X_{1},S}$ is symmetrizable-$X|X_{1}$, then $W_{Y_{1}|X,X_{1},S}$ is also symmetrizable-$X|X_{1}$. Indeed, suppose that $W_{Y|X,X_{1},S}$ is symmetrized by some $J(s|x,x_{1})$ (see Definition 24). Then, for every $x,\tilde{x}\in X$, $x_{1}\in X_{1}$, and $y_{1}\in Y_{1}$,

(A47) $$\begin{array}{cc} \sum_{s\in S}J\left(s|\tilde{x},x_{1}\right)W_{Y_{1}|X,X_{1},S}\left(y_{1}|x,x_{1},s\right)= & \sum_{s\in S}J\left(s|\tilde{x},x_{1}\right)\sum_{y\in Y}W_{Y,Y_{1}|X,X_{1},S}\left(y,y_{1}|x,x_{1},s\right)\\ \overset{\left(a\right)}{=} & \sum_{y\in Y}W_{Y_{1}|Y,X_{1}}\left(y_{1}|y,x_{1}\right)\sum_{s\in S}J\left(s|\tilde{x},x_{1}\right)W_{Y|X,X_{1},X}\left(y|x,x_{1},s\right)\\ \overset{\left(b\right)}{=} & \sum_{y\in Y}W_{Y_{1}|Y,X_{1}}\left(y_{1}|y,x_{1}\right)\sum_{s\in S}J\left(s|x,x_{1}\right)W_{Y|X,X_{1},X}\left(y|\tilde{x},x_{1},s\right)\\ \overset{\left(c\right)}{=} & \sum_{s\in S}J\left(s|x,x_{1}\right)\sum_{y\in Y}W_{Y,Y_{1}|X,X_{1},S}\left(y,y_{1}|\tilde{x},x_{1},s\right)\\ = & \sum_{s\in S}J\left(s|x,x_{1}\right)W_{Y_{1}|X,X_{1},S}\left(y_{1}|\tilde{x},x_{1},s\right)\;, \end{array}$$

where (a) and (c) hold since $W_{Y,Y_{1}|X,X_{1},S}$ is strongly reversely degraded, and (b) holds since $W_{Y|X,X_{1},S}$ is symmetrized by $J(s|x,x_{1})$. This means that $W_{Y_{1}|X,X_{1},S}$ is also symmetrizable-$X|X_{1}$. It can be deduced that given the conditions of part 2, both $W_{Y|X,X_{1},S}$ and $W_{Y_{1}|X,X_{1},S}$ are non-symmetrizable-$X|X_{1}$. Hence, the proof follows from part 1 and Corollary 3. □

## Appendix G. Proof of Lemma 3

The proof is based on generalizing the technique by [56]. Let L be a symmetrizable-$X|X_{1}$. Assume to the contrary that a positive rate R>0 can be achieved. That is, for every ε>0 and sufficiently large *n*, there exists a $\left(2^{nR},n,\varepsilon \right)$ code $C=(f,f_{1},g)$. Hence, the size of the message set is at least 2, i.e.,

(A48) $$\begin{array}{c} M≜2^{nR}\geq 2\;. \end{array}$$

We now show that there exists a distribution $q(s^{n})$ such that the probability of error $P_{e}^{\left(n\right)}\left(q,C\right)$ is bounded from below by a positive constant, in contradiction to the assumption above.

By Definition 3, there exists a conditional distribution J(s|x) that satisfies (24). Then, consider the state sequence distribution $q\left(s^{n}\right)=\frac{1}{M}\sum_{m=1}^{M}J^{n}\left(s^{n}|x^{n}\left(m\right)\right)$, where $J^{n}\left(s^{n}|x^{n}\right)=\prod_{i=1}^{n}J\left(s_{i}|x_{i}\right)$ and $x^{n}\left(m\right)=f\left(m\right)$. For this distribution, the probability of error is given by

(A49) $$\begin{array}{cc} P_{e}^{\left(n\right)}\left(q,C\right)= & \sum_{s^{n}\in S^{n}}\left[\frac{1}{M}\sum_{\tilde{m}=1}^{M}J^{n}\left(s^{n}|x^{n}\left(\tilde{m}\right)\right)\right]\cdot \frac{1}{M}\sum_{m=1}^{M}\sum_{\left(y^{n},y_{1}^{n}\right):g\left(y^{n}\right)\neq m}W^{n}\left(y^{n},y_{1}^{n}|x^{n}\left(m\right),f_{1}^{n}\left(y_{1}^{n}\right),s^{n}\right)\\ = & \frac{1}{2M^{2}}\sum_{m=1}^{M}\sum_{\tilde{m}=1}^{M}\sum_{\left(y^{n},y_{1}^{n}\right):g\left(y^{n}\right)\neq m}\sum_{s^{n}\in S^{n}}W^{n}\left(y^{n},y_{1}^{n}|x^{n}\left(m\right),f_{1}^{n}\left(y_{1}^{n}\right),s^{n}\right)J^{n}\left(s^{n}|x^{n}\left(\tilde{m}\right)\right)\\ & +\frac{1}{2M^{2}}\sum_{m=1}^{M}\sum_{\tilde{m}=1}^{M}\sum_{\left(y^{n},y_{1}^{n}\right):g\left(y^{n}\right)\neq \tilde{m}}\sum_{s^{n}\in S^{n}}W^{n}\left(y^{n},y_{1}^{n}|x^{n}\left(\tilde{m}\right),f_{1}^{n}\left(y_{1}^{n}\right),s^{n}\right)J^{n}\left(s^{n}|x^{n}\left(m\right)\right) \end{array}$$

with $W^{n}\equiv W_{Y^{n},Y_{1}^{n}|X^{n},X_{1}^{n},S^{n}}$ for short notation, where in the last sum we interchanged the summation indices *m* and $\tilde{m}$. Then, consider the last sum, and observe that by (24), we have that

(A50) $$\begin{array}{cc} \sum_{s^{n}\in S^{n}}W^{n}\left(y^{n},y_{1}^{n}|x^{n}\left(\tilde{m}\right),f_{1}^{n}\left(y_{1}^{n}\right),s^{n}\right)J^{n}\left(s^{n}|x^{n}\left(m\right)\right)= & \prod_{i=1}^{n}\left[\sum_{s_{i}\in S}W\left(y_{i},y_{1,i}|x_{i}\left(\tilde{m}\right),f_{1,i}\left(y_{1}^{i-1}\right),s_{i}\right)J\left(s_{i}|x_{i}\left(m\right)\right)\right]\\ = & \prod_{i=1}^{n}\left[\sum_{s_{i}\in S}W\left(y_{i},y_{1,i}|x_{i}\left(m\right),f_{1,i}\left(y_{1}^{i-1}\right),s_{i}\right)J\left(s_{i}|x_{i}\left(\tilde{m}\right)\right)\right]\\ = & \sum_{s^{n}\in S^{n}}W^{n}\left(y^{n},y_{1}^{n}|x^{n}\left(m\right),f_{1}^{n}\left(y_{1}^{n}\right),s^{n}\right)J^{n}\left(s^{n}|x^{n}\left(\tilde{m}\right)\right)\;. \end{array}$$

Substituting (A50) in (A49), we have

$$\begin{array}{cc} P_{e}^{\left(n\right)}\left(q,C\right)= & \frac{1}{2M^{2}}\sum_{m=1}^{M}\sum_{\tilde{m}=1}^{M}\sum_{s^{n}\in S^{n}}[\sum_{\left(y^{n},y_{1}^{n}\right):g\left(y^{n}\right)\neq m}W^{n}\left(y^{n},y_{1}^{n}|x^{n}\left(m\right),f_{1}^{n}\left(y_{1}^{n}\right),s^{n}\right)J^{n}\left(s^{n}|x^{n}\left(\tilde{m}\right)\right)\\ & +\sum_{\left(y^{n},y_{1}^{n}\right):g\left(y^{n}\right)\neq \tilde{m}}W^{n}\left(y^{n},y_{1}^{n}|x^{n}\left(m\right),f_{1}^{n}\left(y_{1}^{n}\right),s^{n}\right)J^{n}\left(s^{n}|x^{n}\left(\tilde{m}\right)\right)] \end{array}$$

(A51) $$\begin{array}{cc} \geq & \frac{1}{2M^{2}}\sum_{m=1}^{M}\sum_{\tilde{m}\neq m}\sum_{s^{n}\in S^{n}}\sum_{y^{n},y_{1}^{n}}W^{n}\left(y^{n},y_{1}^{n}|x^{n}\left(m\right),f_{1}^{n}\left(y_{1}^{n}\right),s^{n}\right)J^{n}\left(s^{n}|x^{n}\left(\tilde{m}\right)\right)\\ = & \frac{M(M-1)}{2M^{2}}\geq \frac{1}{4}\;, \end{array}$$

where the last inequality follows from (A48), hence a positive rate cannot be achieved. □

## Appendix H. Proof of Lemma 4

Let $L=\{W_{Y_{1}|X,X_{1}}W_{Y|Y_{1},X_{1},S}\}$ be a symmetrizable-$X_{1}\times Y_{1}$ degraded AVRC. The proof follows similar lines as in Appendix G. First, assume to the contrary that there exists a $\left(2^{nR},n,\varepsilon \right)$ code $C=(f,f_{1},g)$, with $M≜2^{nR}\geq 2$. By Definition 4, there exists $J(s|x_{1},y_{1})$ that satisfies (28). Hence, defining

(A52) $$\begin{array}{c} q\left(s^{n}\right)=\frac{1}{M}\sum_{m=1}^{M}\sum_{y_{1}^{n}\in Y_{1}}W_{Y_{1}^{n}|X^{n},X_{1}^{n}}\left(y_{1}^{n}|f\left(m\right),f_{1}^{n}\left(y_{1}^{n}\right)\right)J^{n}\left(s^{n}|f_{1}^{n}\left(y_{1}^{n}\right),y_{1}^{n}\right)\;, \end{array}$$

where $J^{n}\left(s^{n}|x_{1}^{n},y_{1}^{n}\right)=\prod_{i=1}^{n}J\left(s_{i}|x_{1,i},y_{1,i}\right)$, we have that

(A53) $$\begin{array}{cc} \sum_{s^{n}\in S^{n}}W^{n}\left(y^{n}|{\tilde{y}}_{1}^{n},f_{1}^{n}\left({\tilde{y}}_{1}^{n}\right),s^{n}\right)J^{n}\left(s^{n}|f_{1}^{n}\left(y_{1}^{n}\right),y_{1}^{n}\right)= & \sum_{s^{n}\in S^{n}}W^{n}\left(y^{n}|y_{1}^{n},f_{1}^{n}\left(y_{1}^{n}\right),s^{n}\right)J^{n}\left(s^{n}|f_{1}^{n}\left({\tilde{y}}_{1}^{n}\right),{\tilde{y}}_{1}^{n}\right)\;. \end{array}$$

By similar manipulations as in Appendix G, we obtain

(A54) $$\begin{array}{c} P_{e}^{\left(n\right)}\left(q,C\right)=\frac{1}{2M^{2}}\sum_{m=1}^{M}\sum_{\tilde{m}=1}^{M}\sum_{y_{1}^{n},{\tilde{y}}_{1}^{n}}W_{Y_{1}^{n}|X^{n},X_{1}^{n}}\left({\tilde{y}}_{1}^{n}|f\left(\tilde{m}\right),f_{1}^{n}\left({\tilde{y}}_{1}^{n}\right)\right)W_{Y_{1}^{n}|X^{n},X_{1}^{n}}\left(y_{1}^{n}|f\left(m\right),f_{1}^{n}\left(y_{1}^{n}\right)\right)\\ \times \sum_{s^{n}\in S^{n}}[\sum_{y^{n}:g\left(y^{n}\right)\neq m}W^{n}\left(y^{n}|y_{1}^{n},f_{1}^{n}\left(y_{1}^{n}\right),s^{n}\right)J^{n}\left(s^{n}|f_{1}^{n}\left({\tilde{y}}_{1}^{n}\right),{\tilde{y}}_{1}^{n}\right)\\ +\sum_{y^{n}:g\left(y^{n}\right)\neq \tilde{m}}W^{n}\left(y^{n}|y_{1}^{n},f_{1}^{n}\left(y_{1}^{n}\right),s^{n}\right)J^{n}\left(s^{n}|f_{1}^{n}\left({\tilde{y}}_{1}^{n}\right),{\tilde{y}}_{1}^{n}\right)]\\ \geq \frac{M(M-1)}{2M^{2}}\geq \frac{1}{4}\;, \end{array}$$

hence a positive rate cannot be achieved. □

## Appendix I. Analysis of Example 1

We show that the random code capacity of the AVRC in Example 1 is given by $C^{⋆}\left(L\right)=\min\left\{\frac{1}{2},1-h\left(\theta \right)\right\}$. As the AVRC is degraded, the random code capacity is given by

(A55) $$\begin{array}{c} C^{⋆}\left(L\right)=R_{PDF}^{⋆}\left(L\right)=R_{CS}^{⋆}\left(L\right)=\max_{p(x,x_{1})}\min\left\{\min_{0\leq q\leq 1}I_{q}\left(X,X_{1};Y\right)\;,\;I\left(X;Y_{1}|X_{1}\right)\right\}\;, \end{array}$$

due to part 2 of Corollary 3, where q≡q(1)=1−q(0). Now, consider the direct part. Set $p\left(x,x_{1}\right)=p\left(x\right)p\left(x_{1}\right)$, where X∼Bernoulli(MM1/2) and $X_{1}∼\mathrm{Bernoulli}\left(MM1\;/\;2\right)$. Then,

(A56) $$\begin{array}{c} I\left(X;Y_{1}|X_{1}\right)=1-h\left(\theta \right)\;,\\ H_{q}\left(Y\right)=\frac{1}{2}\left[-q\log\left(\frac{1}{2}q\right)-\left(1-q\right)\log\left(\frac{1}{2}\left(1-q\right)\right)\right]-\frac{1}{2}\log\left(\frac{1}{2}\right)=1+\frac{1}{2}h\left(q\right)\;,\\ H_{q}\left(Y|X,X_{1}\right)=h\left(q\right)\;. \end{array}$$

Hence,

(A57) $$\begin{array}{cc} C^{⋆}\left(L\right)\geq & \min\left\{\min_{0\leq q\leq 1}\left[1-\frac{1}{2}h\left(q\right)\right],1-h\left(\theta \right)\right\}=\min\left\{\frac{1}{2},1-h\left(\theta \right)\right\}\;. \end{array}$$

As for the converse part, we have the following bounds,

(A58) $$\begin{array}{cc} 1-1C^{⋆}\left(L\right)\leq & \max_{p(x,x_{1})}I\left(X;Y_{1}|X_{1}\right)=1-h\left(\theta \right)\;, \end{array}$$

and

(A59) $$\begin{array}{cc} 1-1C^{⋆}\left(L\right)\leq & \max_{p(x,x_{1})}\min_{0\leq q\leq 1}I_{q}\left(X,X_{1};Y\right)\leq \max_{p(x,x_{1})}\left[H_{q}\left(Y\right)-H_{q}\left(Y|X,X_{1}\right)\right]{|}_{q=\frac{1}{2}}\\ = & \max_{0\leq p\leq 1}\left[1+\frac{1}{2}h\left(p\right)\right]-1=\frac{1}{2}\;, \end{array}$$

where $p≜\mathrm{Pr}\left(X_{1}=1\right)$. □

## Appendix J. Proof of Lemma 5

The proof follows the lines of [5]. Consider an AVRC L = $\{W_{Y|X^{\prime },X_{1}}$ $W_{Y_{1}|X^{\prime \prime },X_{1},S}\}$ with orthogonal sender components. We apply Theorem 1, which states that $R_{PDF}^{⋆}\left(L\right)\leq C^{⋆}\left(L\right)\leq R_{CS}^{⋆}\left(L\right)$.

### Appendix J.1. Achievability Proof

To show achievability, we set $U=X^{\prime \prime }$ and $p\left(x^{\prime },x^{\prime \prime },x_{1}\right)=p\left(x_{1}\right)p\left(x^{\prime }|x_{1}\right)p\left(x^{\prime \prime }|x_{1}\right)$ in the partial decode-forward lower bound $R_{PDF}^{⋆}\left(L\right)≜R_{PDF}\left(L^{Q}\right){|}_{Q=P(S)}$. Hence, by (9),

(A60) $$\begin{array}{cc} R_{PDF}^{⋆}\left(L_{2}\right)\geq & \max_{p\left(x_{1}\right)p\left(x^{\prime }|x_{1}\right)p\left(x^{\prime \prime }|x_{1}\right)}\min\left\{I\left(X^{\prime },X^{\prime \prime },X_{1};Y\right)\;,\;\min_{q(s)}I_{q}\left(X^{\prime \prime };Y_{1}|X_{1}\right)+I\left(X^{\prime };Y|X_{1},X^{\prime \prime }\right)\right\}. \end{array}$$

Now, by (29), we have that $\left(X^{\prime \prime },Y_{1}\right)-\left(X^{\prime },X_{1}\right)-Y$ form a Markov chain. As $\left(X_{1},X^{\prime },X^{\prime \prime }\right)∼p\left(x_{1}\right)p\left(x^{\prime }|x_{1}\right)p\left(x^{\prime \prime }|x_{1}\right)$, it further follows that $\left(X^{\prime \prime },Y_{1}\right)-X_{1}-Y$ form a Markov chain, hence $I\left(X^{\prime },X^{\prime \prime },X_{1};Y\right)=I\left(X^{\prime },X_{1};Y\right)$ and $I\left(X^{\prime };Y|X_{1},X^{\prime \prime }\right)=I\left(X^{\prime };Y|X_{1}\right)$. Thus, (A60) reduces to the expression in the RHS of (30). If $W_{Y_{1}|X^{\prime \prime },X_{1},S}$ is non-symmetrizable-$X^{\prime \prime }|X_{1}$, then (A60) is achievable by deterministic codes as well, due to Corollary 4.

### Appendix J.2. Converse Proof

By (8) and (19), the cutset upper bound takes the following form,

(A61) $$\begin{array}{cc} R_{CS}^{⋆}\left(L\right)= & \min_{q(s)}\max_{p(x^{\prime },x^{\prime \prime },x_{1})}\min\left\{I\left(X^{\prime },X^{\prime \prime },X_{1};Y\right)\;,\;I_{q}\left(X^{\prime },X^{\prime \prime };Y,Y_{1}|X_{1}\right)\right\}\\ = & \max_{p(x^{\prime },x^{\prime \prime },x_{1})}\min\left\{I\left(X^{\prime },X^{\prime \prime },X_{1};Y\right)\;,\;\min_{q(s)}I_{q}\left(X^{\prime },X^{\prime \prime };Y,Y_{1}|X_{1}\right)\right\}\;, \end{array}$$

where the last line is due to the minimax theorem [90]. For the AVRC with orthogonal sender components, as specified by (29), we have the following Markov relations,>

(A62) $$\begin{array}{c} Y_{1}-\left(X^{\prime \prime },X_{1}\right)-\left(X^{\prime },Y\right)\;, \end{array}$$

(A63) $$\begin{array}{c} \left(X^{\prime \prime },Y_{1}\right)-\left(X^{\prime },X_{1}\right)-Y. \end{array}$$

Hence, by (A63), $I\left(X^{\prime },X^{\prime \prime },X_{1};Y\right)=I\left(X^{\prime },X_{1};Y\right)$. As for the second mutual information in the RHS of (A61), by the mutual information chain rule,

(A64) $$\begin{array}{cc} I_{q}\left(X^{\prime },X^{\prime \prime };Y,Y_{1}|X_{1}\right)= & I_{q}\left(X^{\prime \prime };Y_{1}|X_{1}\right)+I_{q}\left(X^{\prime };Y_{1}|X^{\prime \prime },X_{1}\right)+I_{q}\left(X^{\prime },X^{\prime \prime };Y|X_{1},Y_{1}\right)\\ \overset{\left(a\right)}{=} & I_{q}\left(X^{\prime \prime };Y_{1}|X_{1}\right)+I_{q}\left(X^{\prime },X^{\prime \prime };Y|X_{1},Y_{1}\right)\\ \overset{\left(b\right)}{=} & I_{q}\left(X^{\prime \prime };Y_{1}|X_{1}\right)+H_{q}\left(Y|X_{1},Y_{1}\right)-H\left(Y|X^{\prime },X_{1}\right)\\ \overset{\left(c\right)}{\leq } & I_{q}\left(X^{\prime \prime };Y_{1}|X_{1}\right)+I\left(X^{\prime };Y|X_{1}\right) \end{array}$$

where (a) is due to (A62), (b) is due to (A63), and (c) holds since conditioning reduces entropy. Therefore,

(A65) $$\begin{array}{c} R_{CS}^{⋆}\left(L\right)\leq \max_{p(x^{\prime },x^{\prime \prime },x_{1})}\min\left\{I\left(X^{\prime },X_{1};Y\right)\;,\;\min_{q(s)}I_{q}\left(X^{\prime \prime };Y_{1}|X_{1}\right)+I\left(X^{\prime };Y|X_{1}\right)\right\}. \end{array}$$

Without loss of generality, the maximization in (A65) can be restricted to distributions of the form $p\left(x^{\prime },x^{\prime \prime },x_{1}\right)=p\left(x_{1}\right)\cdot$ $p(x^{\prime }|x_{1})\cdot$ $p(x^{\prime \prime }|x_{1})$. □

## Appendix K. Proof of Lemma 6

Consider the Gaussian compound relay channel with SFD under input constraints Ω and $\Omega_{1}$ and state constraint Λ, i.e., $Q=\{q\left(s\right)\;:\;ES^{2}\leq \Lambda \}$.

### Appendix K.1. Achievability Proof

Consider the direct part. Although we previously assumed that the input, state and output alphabets are finite, our results for the compound relay channel can be extended to the continuous case as well, using standard discretization techniques [3] (Section 3.4.1); [55,95]. In particular, Lemma 1 can be extended to the compound relay channel $L^{Q}$ under input constraints Ω and $\Omega_{1}$ and state constraint Λ, by choosing a distribution $p(x^{\prime },x^{\prime \prime },x_{1})$ such that $E(X^{\prime 2}+X^{\prime \prime 2})\leq \Omega$ and $EX_{1}^{2}\leq \Omega_{1}$. Then, the capacity of $L^{Q}$ is bounded by

(A66) $$\begin{array}{cc} C\left(L^{Q}\right)\geq R_{PDF}\left(L^{Q}\right)\geq \max_{\begin{array}{c} p\left(x^{\prime \prime }\right)p\left(x,x_{1}\right)\;:\;\\ E(X^{\prime 2}+X^{\prime \prime 2})\leq \Omega \;,\;\\ EX_{1}^{2}\leq \Omega_{1} \end{array}}\min\{ & \min_{q\left(s\right)\;:\;ES^{2}\leq \Lambda }I_{q}\left(X_{1};Y\right)+\min_{q\left(s\right)\;:\;ES^{2}\leq \Lambda }I_{q}\left(X^{\prime };Y|X_{1}\right)\;,\;\\ & \;I\left(X^{\prime \prime };Y_{1}\right)+\min_{q\left(s\right)\;:\;ES^{2}\leq \Lambda }I_{q}\left(X^{\prime };Y|X_{1}\right)\}\;, \end{array}$$

which follows from the partial decode-forward lower bound by taking $U=X^{\prime \prime }$. Lemma 1 further states that there exists a block Markov code that achieves this rate such that the probability of error decays exponentially as the blocklength increases.

Let 0≤α,ρ≤1, and let $\left(X^{\prime },X^{\prime \prime },X_{1}\right)$ be jointly Gaussian with

(A67) $$\begin{array}{c} X^{\prime }∼N\left(0,\alpha \Omega \right)\;,\;X^{\prime \prime }∼N\left(0,\left(1-\alpha \right)\Omega \right)\;,\;X_{1}∼N\left(0,\Omega_{1}\right)\;, \end{array}$$

where the correlation coefficient of $X^{\prime }$ and $X_{1}$ is ρ, while $X^{\prime \prime }$ is independent of $\left(X^{\prime },X_{1}\right)$. Hence,

(A68) $$\begin{array}{c} I\left(X^{\prime \prime };Y_{1}\right)=\frac{1}{2}\log\left(1+\frac{(1-\alpha )\Omega }{\sigma^{2}}\right). \end{array}$$

Since Gaussian noise is the worst additive noise under variance constraint [96] (Lemma II.2), and as $V\mathrm{ar}\left(X^{\prime }|X_{1}=x_{1}\right)=\left(1-\rho^{2}\right)\alpha \Omega$ for all $x_{1}\in R$, we have that

(A69) $$\begin{array}{c} \min_{q\left(s\right)\;:\;ES^{2}\leq \Lambda }I_{q}\left(X^{\prime };Y|X_{1}\right)=\frac{1}{2}\log\left(1+\frac{(1-\rho^{2})\alpha \Omega }{\Lambda }\right). \end{array}$$

It is left for us to evaluate the first term in the RHS of (A66). Then, by standard whitening transformation, there exist two independent Gaussian random variables $T_{1}$ and $T_{2}$ such that

(A70) $$\begin{array}{c} X^{\prime }+X_{1}=T_{1}+T_{2}\;, \end{array}$$

(A71) $$\begin{array}{c} T_{1}∼N\left(0,\left(1-\rho^{2}\right)\alpha \Omega \right)\;,\;T_{2}∼N\left(0,\Omega_{1}+\rho^{2}\alpha \Omega +2\rho \sqrt{\alpha \Omega \cdot \Omega_{1}}\right). \end{array}$$

Hence, $Y=T_{1}+T_{2}+S$, and as $V\mathrm{ar}\left(X^{\prime }|X_{1}=x_{1}\right)=V\mathrm{ar}\left(T_{1}\right)$ for all $x_{1}\in R$, we have that

(A72) $$\begin{array}{cc} I_{q}\left(X_{1};Y\right)= & H_{q}\left(Y\right)-H_{q}\left(X^{\prime }+S|X_{1}\right)\\ = & H_{q}\left(Y\right)-H_{q}\left(T_{1}+S\right)=I_{q}\left(T_{2};Y\right)\; \end{array}$$

Let $\bar{S}≜T_{1}+S$. Then, since Gaussian noise is the worst additive noise under variance constraint [96] (Lemma II.2),

(A73) $$\begin{array}{cc} \min_{q\left(s\right)\;:\;ES^{2}\leq \Lambda }I_{q}\left(X_{1};Y\right)= & \min_{q\left(s\right)\;:\;ES^{2}\leq \Lambda }I_{q}\left(T_{2};T_{2}+\bar{S}\right)=\frac{1}{2}\log\left(1+\frac{V\mathrm{ar}(T_{2})}{V\mathrm{ar}(T_{1})+\Lambda }\right)\\ = & \frac{1}{2}\log\left(\frac{\Omega_{1}+\rho^{2}\alpha \Omega +2\rho \sqrt{\alpha \Omega \cdot \Omega_{1}}+\Lambda }{(1-\rho^{2})\alpha \Omega +\Lambda }\right). \end{array}$$

Substituting (A68), (A69) and (A73) in the RHS of (A66), we have that

(A74) $$\begin{array}{cc} C(L^{Q})\geq & \max_{0\leq \alpha ,\rho \leq 1}\min\{\frac{1}{2}\log\left(\frac{\Omega_{1}+\rho^{2}\alpha \Omega +2\rho \sqrt{\alpha \Omega \cdot \Omega_{1}}+\Lambda }{(1-\rho^{2})\alpha \Omega +\Lambda }\right)+\frac{1}{2}\log\left(1+\frac{(1-\rho^{2})\alpha \Omega }{\Lambda }\right)\;,\;\\ & \frac{1}{2}\log\left(1+\frac{(1-\alpha )\Omega }{\sigma^{2}}\right)+\frac{1}{2}\log\left(1+\frac{(1-\rho^{2})\alpha \Omega }{\Lambda }\right)\}. \end{array}$$

Observe that the first sum in the RHS of (A74) can be expressed as

(A75) $$\begin{array}{cc} & \frac{1}{2}\log\left(\frac{\Omega_{1}+\rho^{2}\alpha \Omega +2\rho \sqrt{\alpha \Omega \cdot \Omega_{1}}+\Lambda }{(1-\rho^{2})\alpha \Omega +\Lambda }\right)+\frac{1}{2}\log\left(\frac{(1-\rho^{2})\alpha \Omega +\Lambda }{\Lambda }\right)\\ = & \frac{1}{2}\log\left(\frac{\Omega_{1}+\rho^{2}\alpha \Omega +2\rho \sqrt{\alpha \Omega \cdot \Omega_{1}}+\Lambda }{\Lambda }\right)=\frac{1}{2}\log\left(1+\frac{\Omega_{1}+\rho^{2}\alpha \Omega +2\rho \sqrt{\alpha \Omega \cdot \Omega_{1}}}{\Lambda }\right). \end{array}$$

Hence, the direct part follows from (A74). □

### Appendix K.2. Converse Proof

By Lemma 1, $C^{⋆}\left(L^{Q}\right)\leq R_{CS}\left(L^{Q}\right)$. Now, observe that

(A76) $$\begin{array}{cc} R_{CS}\left(L^{Q}\right)= & \min_{q\left(s\right)\;:\;ES^{2}\leq \Lambda }\max_{\begin{array}{c} p\left(x^{\prime \prime }\right)p\left(x,x_{1}\right)\;:\;\\ E(X^{\prime 2}+X^{\prime \prime 2})\leq \Omega \;,\;\\ EX_{1}^{2}\leq \Omega \end{array}}\min\left\{I_{q}\left(X^{\prime },X_{1};Y\right)\;,\;I\left(X^{\prime \prime };Y_{1}\right)+I_{q}\left(X^{\prime };Y|X_{1}\right)\right\}\\ \leq & \max_{\begin{array}{c} p\left(x^{\prime \prime }\right)p\left(x,x_{1}\right)\;:\;\\ E(X^{\prime 2}+X^{\prime \prime 2})\leq \Omega \;,\;\\ EX_{1}^{2}\leq \Omega \end{array}}\min\left\{I_{q}\left(X^{\prime },X_{1};Y\right)\;,\;I\left(X^{\prime \prime };Y_{1}\right)+I_{q}\left(X^{\prime };Y|X_{1}\right)\right\}{|}_{S∼N(0,\Lambda )}\\ = & \max_{0\leq \alpha ,\rho \leq 1}\min\{\frac{1}{2}\log\left(1+\frac{\Omega_{1}+\rho^{2}\alpha \Omega +2\rho \sqrt{\alpha \Omega \cdot \Omega_{1}}}{\Lambda }\right)\;,\;\\ & \frac{1}{2}\log\left(1+\frac{(1-\alpha )\Omega }{\sigma^{2}}\right)+\frac{1}{2}\log\left(1+\frac{(1-\rho^{2})\alpha \Omega }{\Lambda }\right)\}\;, \end{array}$$

where the last equality is due to [5]. □

## Appendix L. Proof of Theorem 2

### Appendix L.1. Achievability Proof

To show that $C^{⋆}\left(L\right)\geq C\left(L^{Q}\right)$, we follow the steps in the proof of Theorem 1, where we replace Ahlswede’s original RT with the modified version in [85, 86] (Lemma 9), plugging $l^{n}\left(s^{n}\right)=\frac{1}{n}\sum_{i=1}^{n}s_{i}^{2}$. Then, by Lemma 6, it follows that

(A77) $$\begin{array}{cc} C^{⋆}\left(L\right)\geq & \max_{0\leq \alpha ,\rho \leq 1}\min\{\frac{1}{2}\log\left(1+\frac{(1+\alpha +2\rho \sqrt{\alpha })\Omega }{\Lambda }\right)\;,\\ & \frac{1}{2}\log\left(1+\frac{(1-\alpha )\Omega }{\sigma^{2}}\right)+\frac{1}{2}\log\left(1+\frac{(1-\rho^{2})\alpha \Omega }{\Lambda }\right)\}. \end{array}$$

The details are omitted. □

### Appendix L.2. Converse Proof

Assume to the contrary that there exists an achievable rate *R* such that

(A78) $$\begin{array}{cc} R> & \max_{0\leq \alpha ,\rho \leq 1}\min\{\frac{1}{2}\log\left(1+\frac{(1+\alpha +2\rho \sqrt{\alpha })\Omega }{\Lambda -\delta }\right)\;,\;\\ & \frac{1}{2}\log\left(1+\frac{(1-\alpha )\Omega }{\sigma^{2}}\right)+\frac{1}{2}\log\left(1+\frac{(1-\rho^{2})\alpha \Omega }{\Lambda -\delta }\right)\} \end{array}$$

using random codes over the Gaussian AVRC L, under input constraints Ω and $\Omega_{1}$ and state constraint Λ, where δ>0 is arbitrarily small. That is, for every ε>0 and sufficiently large *n*, there exists a $\left(2^{nR},n\right)$ random code $C^{\Gamma }=\left(\mu ,\Gamma ,{\left\{C_{\gamma }\right\}}_{\gamma \in \Gamma }\right)$ for the Gaussian AVRC L, under input constraints Ω and $\Omega_{1}$ and state constraint Λ, such that

(A79) $$\begin{array}{c} P_{e|s}\left(C^{\Gamma }\right)\leq \varepsilon \;, \end{array}$$

for all $m\in [1:2^{nR}]$ and $s\in R^{n}$ with ${∥s∥}^{2}\leq n\Lambda$.

Consider using the random code $C^{\Gamma }$ over the Gaussian compound relay channel $L^{Q}$ under state constraint (Λ−δ), i.e., with

(A80) $$\begin{array}{c} Q=\{q\left(s\right)\;:\;ES^{2}\leq \Lambda -\delta \}\;, \end{array}$$

under input constraints Ω and $\Omega_{1}$. Let $\bar{q}\left(s\right)\in Q$ be a given state distribution. Then, define a sequence of i.i.d. random variables ${\bar{S}}_{1},\ldots ,{\bar{S}}_{n}∼\bar{q}\left(s\right)$. Letting $\bar{q}\left(s^{n}\right)≜\prod_{i=1}^{n}\bar{q}\left(s_{i}\right)$, the probability of error is bounded by

(A81) $$\begin{array}{cc} P_{e}^{\left(n\right)}\left(\bar{q},C^{\Gamma }\right)\leq & \sum_{s^{n}\;:\;l^{n}\left(s^{n}\right)\leq \Lambda }{\bar{q}}^{n}\left(s^{n}\right)P_{e|s^{n}}^{\left(n\right)}\left(C^{\Gamma }\right)+\mathrm{Pr}\left(\frac{1}{n}\sum_{i=1}^{n}{\bar{S}}_{i}^{2}>\Lambda \right).\; \end{array}$$

Then, the first sum is bounded by (A79), and the second term vanishes as well by the law of large numbers, since $\bar{q}\left(s\right)$ is in (A80). Hence, the rate *R* in (A78) is achievable for the Gaussian compound relay channel $L^{Q}$, in contradiction to Lemma 6. We deduce that the assumption is false, and (A78) cannot be achieved. □

## Appendix M. Proof of Theorem 3

Consider the Gaussian AVRC L with SFD under input constraints Ω and $\Omega_{1}$ and state constraint Λ. In the proof, we modify the techniques by Csiszár and Narayan [87]. In the direct part, we use their correlation binning technique within the decode-forward coding scheme, and in the converse part, we consider a jamming scheme which simulates the transmission sum by the encoder and the relay.

### Appendix M.1. Lower Bound

We construct a block Markov code using backward minimum-distance decoding in two steps. The encoders use *B* blocks, each consists of *n* channel uses, to convey (B−1) independent messages to the receiver, where each message $M_{b}$, for b∈[1:B−1], is divided into two independent messages. That is, $M_{b}=\left(M_{b}^{\prime },M_{b}^{\prime \prime }\right)$, where $M_{b}^{\prime }$ and $M_{b}^{\prime \prime }$ are uniformly distributed, i.e.,

(A82) $$\begin{array}{c} M_{b}^{\prime }∼\mathrm{Unif}\left[1:2^{nR^{\prime }}\right]\;,\;M_{b}^{\prime \prime }∼\mathrm{Unif}\left[1:2^{nR^{\prime \prime }}\right]\;,\;\mathrm{with}\;R^{\prime }+R^{\prime \prime }=R\;, \end{array}$$

for b∈[1:B−1]. For convenience of notation, set $M_{0}^{\prime }=M_{B}^{\prime }\equiv 1$ and $M_{0}^{\prime \prime }=M_{B}^{\prime \prime }\equiv 1$. The average rate $\frac{B-1}{B}\cdot R$ is arbitrarily close to *R*.

*Codebook Construction*: Fix 0≤α,ρ≤1 with

(A83) $$\begin{array}{cc} & (1-\rho^{2})\alpha \Omega >\Lambda \;, \end{array}$$

(A84) $$\begin{array}{cc} & \frac{\Omega_{1}}{\Omega }{\left(\sqrt{\Omega_{1}}+\rho \sqrt{\alpha \Omega }\right)}^{2}>\Lambda +\left(1-\rho^{2}\right)\alpha \Omega . \end{array}$$

We construct *B* codebooks $F_{b}$ of the following form,

(A85) $$\begin{array}{c} F_{b}=\left\{\left(x_{1}\left(m_{b-1}^{\prime }\right),x^{\prime }\left(m_{b}^{\prime },m_{b}^{\prime \prime }|m_{b-1}^{\prime }\right),x^{\prime \prime }\left(m_{b}^{\prime }\right)\right)\;:\;m_{b-1}^{\prime },m_{b}^{\prime }\in \left[1:2^{nR^{\prime }}\right]\;,\;m_{b}^{\prime \prime }\in \left[1:2^{nR^{\prime \prime }}\right]\right\}\;, \end{array}$$

for b∈[2:B−1]. The codebooks $F_{1}$ and $F_{B}$ have the same form, with fixed $m_{0}^{\prime }=m_{B}^{\prime }\equiv 1$ and $m_{0}^{\prime \prime }=m_{B}^{\prime \prime }\equiv 1$.

The sequences $x^{\prime \prime }\left(m_{b}^{\prime }\right)$, $m_{b}^{\prime }\in \left[1:2^{nR^{\prime }}\right]$ are chosen as follows. Observe that the channel from the sender to the relay, $Y_{1}=X^{\prime \prime }+Z$, does not depend on the state. Thus, by Shannon’s well-known result on the point-to-point Gaussian channel [97], the message $m_{b}^{\prime }$ can be conveyed to the relay reliably, under input constraint (1−α)Ω, provided that $R^{\prime }<\frac{1}{2}\log\left(1+\frac{(1-\alpha )\Omega }{\sigma^{2}}\right)-\delta_{1}$, where $\delta_{1}$ is arbitrarily small (see also [98] (Chapter 9)). That is, for every ε>0 and sufficiently large *n*, there exists a $\left(2^{nR^{\prime }},n,\varepsilon \right)$ code $C^{\prime \prime }=\left(x^{\prime \prime }\left(m_{b}^{\prime }\right),g_{1}\left(y_{1,b}\right)\right)$, such that ${∥x^{\prime \prime }\left(m_{b}^{\prime }\right)∥}^{2}\leq n\left(1-\alpha \right)\Omega$ for all $m_{b}^{\prime }\in \left[1:2^{nR^{\prime }}\right]$.

Next, we choose the sequences $x_{1}\left(m_{b-1}^{\prime }\right)$ and $x^{\prime }\left(m_{b}^{\prime },m_{b}^{\prime \prime }\right|$ $m_{b-1}^{\prime })$, for $m_{b-1}^{\prime },$ $m_{b}^{\prime }\in \left[1:2^{nR^{\prime }}\right]$, $m_{b}^{\prime \prime }\in \left[1:2^{nR^{\prime \prime }}\right]$. Applying Lemma 7 by [87] repeatedly yields the following.

**Lemma** **A1.**
*For every ε>0, $8\sqrt{\varepsilon }<\eta <1$, K>2ε, $2\varepsilon \leq R^{\prime }\leq K$, $2\varepsilon \leq R^{\prime \prime }\leq K$, and $n\geq n_{0}\left(\varepsilon ,\eta ,K\right)$,*

1. *there exist $2^{nR^{\prime }}$ unit vectors,*
  (A86) $$\begin{array}{c} a\left(m_{b-1}^{\prime }\right)\in R^{n}\;,\;m_{b-1}^{\prime }\in \left[1:2^{nR^{\prime }}\right]\;, \end{array}$$
  *such that for every unit vector $c\in R^{n}$ and 0≤θ,ζ≤1,*
  (A87) $$\begin{array}{c} \left|\left\{{\tilde{m}}_{b-1}^{\prime }\in \left[1:2^{nR^{\prime }}\right]\;:\;\;\langle a\left({\tilde{m}}_{b-1}^{\prime }\right),c\rangle \geq \theta \right\}\right|\leq 2^{n\left({\left[R^{\prime }+\frac{1}{2}\log\left(1-\theta^{2}\right)\right]}_{+}+\varepsilon \right)}\;, \end{array}$$
  *and if θ≥η and $\theta^{2}+\zeta^{2}>1+\eta -2^{-2R^{\prime }}$, then*
  (A88) $$\begin{array}{c} \frac{1}{2^{nR^{\prime }}}|\{m_{b-1}^{\prime }\in \left[1:2^{nR^{\prime }}\right]\;:\;\;|\langle a\left({\tilde{m}}_{b-1}^{\prime }\right),a\left(m_{b-1}^{\prime }\right)\rangle \left|\geq \theta \;,\;\right|\langle a\left({\tilde{m}}_{b-1}^{\prime }\right),c\rangle |\geq \zeta \;,\;\\ \;\mathrm{for}\;\mathrm{some}\;{\tilde{m}}_{b-1}^{\prime }\neq m_{b-1}^{\prime }\}|\leq 2^{-n\varepsilon }\;. \end{array}$$
2. *Furthermore, for every $m_{b}^{\prime }\in \left[1:2^{nR^{\prime }}\right]$, there exist $2^{nR^{\prime \prime }}$ unit vectors,*
  (A89) $$\begin{array}{c} v\left(m_{b}^{\prime },m_{b}^{\prime \prime }\right)\in R^{n}\;,\;m_{b}^{\prime \prime }\in \left[1:2^{nR^{\prime \prime }}\right]\;, \end{array}$$
  *such that for every unit vector $c\in R^{n}$ and 0≤θ,ζ≤1,*
  (A90) $$\begin{array}{c} \left|\left\{{\tilde{m}}_{b}^{\prime \prime }\in \left[1:2^{nR^{\prime \prime }}\right]\;:\;\;\langle v\left(m_{b}^{\prime },{\tilde{m}}_{b}^{\prime \prime }\right),c\rangle \geq \theta \right\}\right|\leq 2^{n\left({\left[R^{\prime \prime }+\frac{1}{2}\log\left(1-\theta^{2}\right)\right]}_{+}+\varepsilon \right)}\;, \end{array}$$
  *and if θ≥η and $\theta^{2}+\zeta^{2}>1+\eta -2^{-2R^{\prime \prime }}$, then*
  (A91) $$\begin{array}{c} \frac{1}{2^{nR^{\prime \prime }}}|\{m_{b}^{\prime \prime }\in \left[1:2^{nR^{\prime \prime }}\right]\;:\;\;|\langle v\left(m_{b}^{\prime },{\tilde{m}}_{b}^{\prime \prime }\right),v\left(m_{b}^{\prime },m_{b}^{\prime \prime }\right)\rangle \left|\geq \theta \;,\;\right|\langle v\left(m_{b}^{\prime },{\tilde{m}}_{b}^{\prime \prime }\right),c\rangle |\geq \zeta \;,\;\\ \;\mathrm{for}\;\mathrm{some}\;{\tilde{m}}_{b}^{\prime \prime }\neq m_{b}^{\prime \prime }\}|\leq 2^{-n\varepsilon }\;. \end{array}$$

Then, define

(A92) $$\begin{array}{c} x_{1}\left(m_{b-1}^{\prime }\right)=\sqrt{n\gamma (\Omega -\delta )}\cdot a\left(m_{b-1}^{\prime }\right)\;,\\ x^{\prime }\left(m_{b}^{\prime },m_{b}^{\prime \prime }|m_{b-1}^{\prime }\right)=\rho \sqrt{\alpha \gamma^{-1}}\cdot x_{1}\left(m_{b-1}^{\prime }\right)+\beta \cdot v\left(m_{b}^{\prime },m_{b}^{\prime \prime }\right)\;, \end{array}$$

where

(A93) $$\begin{array}{cc} \beta ≜ & \sqrt{n\left(1-\rho^{2}\right)\alpha \left(\Omega -\delta \right)}\;,\;\gamma ≜\Omega_{1}\;/\;\Omega . \end{array}$$

Note that ${∥x_{1}\left(m_{b-1}^{\prime }\right)∥}^{2}=n\gamma \left(\Omega -\delta \right)<n\Omega_{1}$, for all $m_{b-1}^{\prime }\in \left[1:2^{nR^{\prime }}\right]$. On the other hand, ${∥x^{\prime }\left(m_{b}^{\prime },m_{b}^{\prime \prime }|m_{b-1}^{\prime }\right)∥}^{2}$ could be greater than nαΩ due to the possible correlation between $x_{1}\left(m_{b-1}^{\prime }\right)$ and $v(m_{b}^{\prime },m_{b}^{\prime \prime })$.

*Encoding*: Let $\left(m_{1}^{\prime },m_{1}^{\prime \prime },\ldots ,m_{B-1}^{\prime },m_{B-1}^{\prime \prime }\right)$ be a sequence of messages to be sent. In block b∈[1:B], if ${∥x^{\prime }\left(m_{b}^{\prime },m_{b}^{\prime \prime }|m_{b-1}^{\prime }\right)∥}^{2}$ ≤nαΩ, transmit $\left(x^{\prime }\left(m_{b}^{\prime },m_{b}^{\prime \prime }|m_{b-1}^{\prime }\right),x^{\prime \prime }\left(m_{b}^{\prime }\right)\right)$. Otherwise, transmit $\left(0,x^{\prime \prime }\left(m_{b}^{\prime }\right)\right)$.

*Relay Encoding*: In block 1, the relay transmits $x_{1}\left(1\right)$. At the end of block b∈[1:B−1], the relay receives $y_{1,b}$, and finds an estimate ${\bar{m}}_{b}^{\prime }=g_{1}\left(y_{1,b}\right)$. In block b+1, the relay transmits $x_{1}\left({\bar{m}}_{b}^{\prime }\right)$.

*Backward Decoding*: Once all blocks ${\left(y_{b}\right)}_{b=1}^{B}$ are received, decoding is performed backwards. Set ${\hat{m}}_{0}^{\prime }={\hat{m}}_{0}^{\prime \prime }\equiv 1$. For b=B−1,B−2,…,1, find a unique ${\hat{m}}_{b}^{\prime }\in \left[1:2^{nR^{\prime }}\right]$ such that

(A94) $$\begin{array}{c} ∥y_{b+1}-\left(1+\rho \sqrt{\alpha \gamma^{-1}}\right)x_{1}\left({\hat{m}}_{b}^{\prime }\right)∥\leq ∥y_{b+1}-\left(1+\rho \sqrt{\alpha \gamma^{-1}}\right)x_{1}\left(m_{b}^{\prime }\right)∥\;,\;\mathrm{for}\;\mathrm{all}\;m_{b}^{\prime }\in \left[1:2^{nR}\right]. \end{array}$$

If there is more than one such ${\hat{m}}_{b}^{\prime }\in \left[1:2^{nR^{\prime }}\right]$, declare an error.

Then, the decoder uses ${\hat{m}}_{1}^{\prime },\ldots ,{\hat{m}}_{B-1}^{\prime }$ as follows. For b=B−1,B−2,…,1, find a unique ${\hat{m}}_{b}^{\prime \prime }\in \left[1:2^{nR^{\prime \prime }}\right]$ such that

(A95) $$\begin{array}{c} ∥y_{b}-x_{1}\left({\hat{m}}_{b-1}^{\prime }\right)-x^{\prime }\left({\hat{m}}_{b}^{\prime },{\hat{m}}_{b}^{\prime \prime }|{\hat{m}}_{b-1}^{\prime }\right)∥\leq ∥y_{b}-x_{1}\left({\hat{m}}_{b-1}^{\prime }\right)-x^{\prime }\left({\hat{m}}_{b}^{\prime },m_{b}^{\prime \prime }|{\hat{m}}_{b-1}^{\prime }\right)∥\;,\;\mathrm{for}\;\mathrm{all}\;m_{b}^{\prime \prime }\in \left[1:2^{nR^{\prime \prime }}\right]. \end{array}$$

If there is more than one such ${\hat{m}}_{b}^{\prime \prime }\in \left[1:2^{nR^{\prime \prime }}\right]$, declare an error.

*Analysis of Probability of Error*: Fix $s\in S^{n}$, and let

(A96) $$\begin{array}{c} c_{0}≜\frac{s}{∥s∥}. \end{array}$$

The error event is bounded by the union of the following events. For b∈[1:B−1], define

(A97) $$\begin{array}{c} E_{1}\left(b\right)=\left\{{\bar{M}}_{b}^{\prime }\neq M_{b}^{\prime }\right\}\;,\;E_{2}\left(b\right)=\left\{{\hat{M}}_{b}^{\prime }\neq M_{b}^{\prime }\right\}\;,\;E_{3}\left(b\right)=\left\{{\hat{M}}_{b}^{\prime \prime }\neq M_{b}^{\prime \prime }\right\}. \end{array}$$

Then, the conditional probability of error given the state sequence s is bounded by

(A98) $$\begin{array}{cc} P_{e|s}\left(C\right)\leq & \sum_{b=1}^{B-1}\mathrm{Pr}\left(E_{1}\left(b\right)\right)+\sum_{b=1}^{B-1}\mathrm{Pr}\left(E_{2}\left(b\right)\cap E_{1}^{c}\left(b\right)\right)+\sum_{b=1}^{B-1}\mathrm{Pr}\left(E_{3}\left(b\right)\cap E_{1}^{c}\left(b-1\right)\cap E_{2}^{c}\left(b\right)\cap E_{2}^{c}\left(b-1\right)\right)\;, \end{array}$$

with $E_{1}\left(0\right)=E_{2}\left(0\right)=\emptyset$, where the conditioning on S=s is omitted for convenience of notation. Recall that we have defined $C^{\prime \prime }$ as a $\left(2^{nR^{\prime }},n,\varepsilon \right)$ code for the point-to-point Gaussian channel $Y_{1}=X^{\prime \prime }+Z$. Hence, the first sum in the RHS of (A98) is bounded by B·ε, which is arbitrarily small.

To be more concise, we only give the details for erroneous decoding of $M_{b}^{\prime }$ at the receiver. Consider the following events,

(A99) $$\begin{array}{c} E_{2}\left(b\right)=\left\{∥Y_{b+1}-\left(1+\rho \sqrt{\alpha \gamma^{-1}}\right)x_{1}\left({\tilde{m}}_{b}^{\prime }\right)∥\leq ∥Y_{b+1}-\left(1+\rho \sqrt{\alpha \gamma^{-1}}\right)x_{1}\left(M_{b}^{\prime }\right)∥\;,\;\mathrm{for}\;\mathrm{some}\;{\tilde{m}}_{b}^{\prime }\neq M_{b}^{\prime }\right\}\;,\\ E_{2,1}\left(b\right)=\left\{\right|\langle a\left(M_{b}^{\prime }\right),c_{0}\rangle \left|\geq \eta \right\}\;,\\ E_{2,2}\left(b\right)=\left\{\right|\langle a\left(M_{b}^{\prime }\right),v\left(M_{b+1}\right)\rangle \left|\geq \eta \right\}\\ E_{2,3}\left(b\right)=\left\{\right|\langle v\left(M_{b+1}\right),c_{0}\rangle \left|\geq \eta \right\}\\ {\tilde{E}}_{2}\left(b\right)=E_{2}\left(b\right)\cap E_{1}^{c}\left(b\right)\cap E_{2,1}^{c}\left(b\right)\cap E_{2,2}^{c}\left(b\right)\cap E_{2,3}^{c}\left(b\right)\;, \end{array}$$

where $M_{b+1}=\left(M_{b+1}^{\prime },M_{b+1}^{\prime \prime }\right)$. Then,

(A100) $$\begin{array}{cc} E_{2}\left(b\right)\cap E_{1}^{c}\left(b\right)\subseteq & E_{2,1}\left(b\right)\cup E_{2,2}\left(b\right)\cup E_{2,3}\left(b\right)\cup \left(E_{2}\left(b\right)\cap E_{1}^{c}\left(b\right)\right)\\ = & E_{2,1}\left(b\right)\cup E_{2,2}\left(b\right)\cup E_{2,3}\left(b\right)\cup {\tilde{E}}_{2}\left(b\right). \end{array}$$

Hence, by the union of events bound, we have that

(A101) $$\begin{array}{cc} \mathrm{Pr}\left(E_{2}\left(b\right)\cap E_{1}^{c}\left(b\right)\right)\leq & \mathrm{Pr}\left(E_{2,1}\left(b\right)\right)+\mathrm{Pr}\left(E_{2,2}\left(b\right)\right)+\mathrm{Pr}\left(E_{2,3}\left(b\right)\right)+\mathrm{Pr}\left({\tilde{E}}_{2}\left(b\right)\right). \end{array}$$

By Lemma A1, given $R^{\prime }>-\frac{1}{2}\log\left(1-\eta^{2}\right)$, the first term is bounded by

(A102) $$\begin{array}{cc} \mathrm{Pr}\left(E_{2,1}\left(b\right)\right)= & \mathrm{Pr}\left(\langle a\left(M_{b}^{\prime }\right),c_{0}\rangle \geq \eta \right)+\mathrm{Pr}\left(\langle a\left(M_{b}^{\prime }\right),-c_{0}\rangle \geq \eta \right)\\ \leq & 2\cdot \frac{1}{2^{nR^{\prime }}}\cdot 2^{n\left(R^{\prime }+\frac{1}{2}\log\left(1-\eta^{2}\right)+\varepsilon \right)}\leq 2\cdot 2^{n\left(-\frac{1}{2}\eta^{2}+\varepsilon \right)}\;, \end{array}$$

since log(1+t)≤t for t∈R. As $\eta^{2}\geq 8\varepsilon$, the last expression tends to zero as n→∞. Similarly, $\mathrm{Pr}\left(E_{2,2}\left(b\right)\right)$ and $\mathrm{Pr}\left(E_{2,3}\left(b\right)\right)$ tend to zero as well. Moving to the fourth term in the RHS of (A101), observe that for a sufficiently small ε and η, the event $E_{2,2}^{c}\left(b\right)$ implies that ${∥x^{\prime }\left(M_{b+1}|M_{b}^{\prime }\right)∥}^{2}\leq n\alpha \Omega$, while the event $E_{1}^{c}\left(b\right)$ means that ${\bar{M}}_{b}^{\prime }=M_{b}^{\prime }$. Hence, the encoder transmits $\left(x^{\prime }\left(M_{b+1}|M_{b}^{\prime }\right),x^{\prime \prime }\left(M_{b+1}^{\prime }\right)\right)$, the relay transmits $x_{1}\left(M_{b}^{\prime }\right)$, and we have that

(A103) $$\begin{array}{cc} & {∥Y_{b+1}-\left(1+\rho \sqrt{\alpha \gamma^{-1}}\right)x_{1}\left({\tilde{m}}_{b}^{\prime }\right)∥}^{2}-{∥Y_{b+1}-\left(1+\rho \sqrt{\alpha \gamma^{-1}}\right)x_{1}\left(M_{b}^{\prime }\right)∥}^{2}\\ = & {∥\left(1+\rho \sqrt{\alpha \gamma^{-1}}\right)x_{1}\left(M_{b}^{\prime }\right)+\beta v\left(M_{b+1}\right)+s-\left(1+\rho \sqrt{\alpha \gamma^{-1}}\right)x_{1}\left({\tilde{m}}_{b}^{\prime }\right)∥}^{2}-{∥\beta v(M_{b+1})+s∥}^{2}\\ = & 2{\left(1+\rho \sqrt{\alpha \gamma^{-1}}\right)}^{2}\left(\frac{1}{2}{∥x_{1}\left(M_{b}^{\prime }\right)∥}^{2}+\frac{1}{2}{∥x_{1}\left({\tilde{m}}_{b}^{\prime }\right)∥}^{2}-\langle x_{1}\left({\tilde{m}}_{b}^{\prime }\right),x_{1}\left(M_{b}^{\prime }\right)\rangle \right)\\ & +2\left(1+\rho \sqrt{\alpha \gamma^{-1}}\right)\left(\langle x_{1}\left(M_{b}^{\prime }\right),\beta v\left(M_{b+1}\right)+s\rangle -\langle x_{1}\left({\tilde{m}}_{b}^{\prime }\right),\beta v\left(M_{b+1}\right)+s\rangle \right) \end{array}$$

Then, since ${∥x_{1}\left(m_{b}^{\prime }\right)∥}^{2}=n\gamma \left(\Omega -\delta \right)$ for all $m_{b}^{\prime }\in \left[1:2^{nR^{\prime }}\right]$, we have that

(A104) $$\begin{array}{cc} E_{2}\left(b\right)\cap E_{1}^{c}\left(b\right)\cap E_{2,2}^{c}\left(b\right)\subseteq & \{\left(1+\rho \sqrt{\alpha \gamma^{-1}}\right)\langle x_{1}\left({\tilde{m}}_{b}^{\prime }\right),x_{1}\left(M_{b}^{\prime }\right)\rangle +\langle x_{1}\left({\tilde{m}}_{b}^{\prime }\right),\beta v\left(M_{b+1}\right)+s\rangle \geq \\ & \;n\left(1+\rho \sqrt{\alpha \gamma^{-1}}\right)\gamma \left(\Omega -\delta \right)+\langle x_{1}\left(M_{b}^{\prime }\right),\beta v\left(M_{b+1}\right)+s\rangle \;,\;\mathrm{for}\;\mathrm{some}\;{\tilde{m}}_{b}^{\prime }\neq M_{b}^{\prime }\}\;. \end{array}$$

Observe that for sufficiently small ε and η, the event $E_{2,1}^{c}\left(b\right)\cap E_{2,2}^{c}\left(b\right)\cap E_{2,3}^{c}\left(b\right)$ implies that

(A105) $$\begin{array}{c} \langle x_{1}\left(M_{b}^{\prime }\right),\beta v\left(M_{b+1}\right)+s\rangle \geq -\delta \;, \end{array}$$

and

(A106) $$\begin{array}{c} {∥\beta v(M_{b+1})+s∥}^{2}\leq n\left[\left(1-\rho^{2}\right)\alpha \Omega +\Lambda \right]. \end{array}$$

Hence, by (A104) and (A105),

(A107) $$\begin{array}{cc} & {\tilde{E}}_{2}\left(b\right)=E_{2}\left(b\right)\cap E_{1}^{c}\left(b\right)\cap E_{2,1}^{c}\left(b\right)\cap E_{2,2}^{c}\left(b\right)\cap E_{2,3}^{c}\left(b\right)\\ \subseteq & \{\left(1+\rho \sqrt{\alpha \gamma^{-1}}\right)\langle x_{1}\left({\tilde{m}}_{b}^{\prime }\right),x_{1}\left(M_{b}^{\prime }\right)\rangle +\langle x_{1}\left({\tilde{m}}_{b}^{\prime }\right),\beta v\left(M_{b+1}\right)+s\rangle \geq n\left(1+\rho \sqrt{\alpha \gamma^{-1}}\right)\gamma \left(\Omega -2\delta \right)\;,\;\\ & \;\mathrm{for}\;\mathrm{some}\;{\tilde{m}}_{b}^{\prime }\neq M_{b}^{\prime }\}. \end{array}$$

Dividing both sides of the inequality by $n(1+\rho \sqrt{\alpha \gamma^{-1}})$, we obtain

(A108) $$\begin{array}{c} {\tilde{E}}_{2}\left(b\right)\subseteq \left\{\frac{1}{n}\langle x_{1}\left({\tilde{m}}_{b}^{\prime }\right),x_{1}\left(M_{b}^{\prime }\right)\rangle +\frac{\langle x_{1}\left({\tilde{m}}_{b}^{\prime }\right),\beta v\left(M_{b+1}\right)+s\rangle }{n(1+\rho \sqrt{\alpha \gamma^{-1}})}\geq \gamma \left(\Omega -2\delta \right)\;,\;\mathrm{for}\;\mathrm{some}\;{\tilde{m}}_{b}^{\prime }\neq M_{b}^{\prime }\right\}. \end{array}$$

Next, we partition the set of values of $\frac{1}{n}\langle x_{1}\left({\tilde{m}}_{b}^{\prime }\right),x_{1}\left(M_{b}^{\prime }\right)\rangle$ to *K* bins. Let $\tau_{1}<\tau_{2}<\cdots <\tau_{K}$ be such partition, where

(A109) $$\begin{array}{c} \tau_{1}=\gamma \left(\Omega -2\delta \right)-\frac{\sqrt{\left(\Omega -\delta \right)[\left(1-\rho^{2}\right)\alpha \Omega +\Lambda ]}}{1+\rho \sqrt{\alpha \gamma^{-1}}}\;,\;\tau_{K}=\gamma \left(\Omega -3\delta \right)\;,\\ \tau_{k+1}-\tau_{k}\leq \gamma \cdot \delta \;,\;\mathrm{for}\;k=[1:K-1]\;, \end{array}$$

where *K* is a finite constant which is independent of *n*, as in Lemma A1. By (A106) and (A108), given the event ${\tilde{E}}_{2}\left(b\right)$, we have that

(A110) $$\begin{array}{c} \frac{1}{n}\langle x_{1}\left({\tilde{m}}_{b}^{\prime }\right),x_{1}\left(M_{b}^{\prime }\right)\rangle \geq \tau_{1}>0\;, \end{array}$$

where the last inequality is due to (A84), for sufficiently small δ>0. To see this, observe that the inequality in (A84) is strict, and it implies that

(A111) $$\begin{array}{c} \sqrt{\gamma }\cdot \left(\sqrt{\gamma \Omega }+\rho \sqrt{\alpha \Omega }\right)>\sqrt{(1-\rho^{2})\alpha \Omega +\Lambda }. \end{array}$$

Hence, for sufficiently small δ>0, $\tau_{1}>0$ as

(A112) $$\begin{array}{c} \tau_{1}=\frac{\sqrt{\Omega -2\delta }}{1+\rho \sqrt{\alpha \gamma^{-1}}}\cdot \left(\sqrt{\gamma }\left(\sqrt{\gamma (\Omega -2\delta )}+\rho \sqrt{\alpha (\Omega -2\delta )}\right)-\sqrt{\frac{\Omega -\delta }{\Omega -2\delta }\left[\left(1-\rho^{2}\right)\alpha \Omega +\Lambda \right]}\right). \end{array}$$

Furthermore, if $\tau_{k}\leq \frac{1}{n}\langle x_{1}\left({\tilde{m}}_{b}^{\prime }\right),x_{1}\left(M_{b}^{\prime }\right)\rangle <\tau_{k+1}$, then

(A113) $$\begin{array}{c} \frac{\langle x_{1}\left({\tilde{m}}_{b}^{\prime }\right),\beta v\left(M_{b+1}\right)+s\rangle }{n(1+\rho \sqrt{\alpha \gamma^{-1}})}\geq \gamma \left(\Omega -2\delta \right)-\tau_{k+1}\geq \gamma \left(\Omega -3\delta \right)-\tau_{k}. \end{array}$$

Thus,

(A114) $$\begin{array}{c} \mathrm{Pr}\left({\tilde{E}}_{2}\left(b\right)\right)\leq \sum_{k=1}^{K-1}\mathrm{Pr}(\frac{1}{n}\left|\langle x_{1}\left({\tilde{m}}_{b}^{\prime }\right),x_{1}\left(M_{b}^{\prime }\right)\rangle \right|\geq \tau_{k}\;,\;\frac{\left|\langle x_{1}\left({\tilde{m}}_{b}^{\prime }\right),\beta v\left(M_{b+1}\right)+s\rangle \right|}{n(1+\rho \sqrt{\alpha \gamma^{-1}})}\geq \gamma \left(\Omega -3\delta \right)-\tau_{k}\;,\;\\ \mathrm{for}\;\mathrm{some}\;{\tilde{m}}_{b}^{\prime }\neq M_{b}^{\prime })+\mathrm{Pr}\left(\frac{1}{n}\left|\langle x_{1}\left({\tilde{m}}_{b}^{\prime }\right),x_{1}\left(M_{b}^{\prime }\right)\rangle \right|\geq \tau_{K}\;,\;\mathrm{for}\;\mathrm{some}\;{\tilde{m}}_{b}^{\prime }\neq M_{b}^{\prime }\right). \end{array}$$

By (A106), this can be further bounded by

(A115) $$\begin{array}{cc} \mathrm{Pr}\left({\tilde{E}}_{2}\left(b\right)\right)\leq & \sum_{k=1}^{K}\mathrm{Pr}\left(|\langle a\left({\tilde{m}}_{b}^{\prime }\right),a\left(M_{b}^{\prime }\right)\rangle |\geq \theta_{k}\;,\;\left|\langle a\left({\tilde{m}}_{b}^{\prime }\right),c^{\prime }\left(M_{b+1}\right)\rangle \right|\geq \mu_{k}\;,\;\mathrm{for}\;\mathrm{some}\;{\tilde{m}}_{b}^{\prime }\neq M_{b}^{\prime }\right)\;, \end{array}$$

where

(A116) $$\begin{array}{c} c^{\prime }\left(m_{b+1}\right)≜\frac{\beta v(m_{b+1})+s}{∥\beta v(m_{b+1})+s∥}\;, \end{array}$$

and

(A117) $$\begin{array}{c} \theta_{k}≜\frac{\tau_{k}}{\gamma (\Omega -\delta )}\;,\;\zeta_{k}≜\frac{\left(1+\rho \sqrt{\alpha \gamma^{-1}}\right)\left(\gamma \left(\Omega -3\delta \right)-\tau_{k}\right)}{\sqrt{\gamma \left(\Omega -\delta \right)(\left(1-\rho^{2}\right)\alpha \Omega +\Lambda )}}\;,\;\mathrm{for}\;k\in [1:K-1]\;;\;\theta_{K}≜\frac{\tau_{K}}{\Omega -\delta }\;,\;\zeta_{K}=0. \end{array}$$

By Lemma A1, the RHS of (A115) tends to zero as n→∞ provided that

(A118) $$\begin{array}{c} \theta_{k}\geq \eta \;\mathrm{and}\;\;\theta_{k}^{2}+\zeta_{k}^{2}>1+\eta -e^{-2R^{\prime }}\;,\;\mathrm{for}\;k=[1:K]. \end{array}$$

For sufficiently small ε and η, we have that $\eta \leq \theta_{1}=\frac{\tau_{1}}{\gamma (\Omega -\delta )}$, hence the first condition is met. Then, observe that the second condition is equivalent to $G\left(\tau_{k}\right)>1+\eta -e^{-2R^{\prime }}$, for k∈[1:K−1], where

(A119) $$\begin{array}{c} G\left(\tau \right)={\left(A\tau \right)}^{2}+D^{2}{\left(L-\tau \right)}^{2}\;,\; \end{array}$$

with

(A120) $$\begin{array}{c} A=\frac{1}{\gamma (\Omega -\delta )}\;,\;D=\frac{1+\rho \sqrt{\alpha \gamma^{-1}}}{\sqrt{\gamma \left(\Omega -\delta \right)(\left(1-\rho^{2}\right)\alpha \Omega +\Lambda )}}\;,\;L=\gamma \left(\Omega -3\delta \right). \end{array}$$

By differentiation, we have that the minimum value of this function is given by $\min_{\tau_{1}\leq \tau \leq \tau_{K}}G\left(\tau \right)=\frac{A^{2}D^{2}L^{2}}{A^{2}+D^{2}}=\frac{D^{2}}{A^{2}+D^{2}}-\delta_{1}$, where $\delta_{1}\to 0$ as δ→0. Thus, the RHS of (A115) tends to zero as n→∞, provided that

(A121) $$\begin{array}{cc} R^{\prime }< & -\frac{1}{2}\log\left(1+\eta -\frac{D^{2}}{A^{2}+D^{2}}+\delta_{1}\right)\\ = & -\frac{1}{2}\log\left(\eta +\delta_{1}+\frac{(1-\rho^{2})\alpha \Omega +\Lambda }{\left(\gamma +\alpha +2\rho \sqrt{\alpha \gamma }\right)\Omega +\Lambda -\delta \gamma {\left(1+\rho \sqrt{\alpha \gamma^{-1}}\right)}^{2}}\right). \end{array}$$

This is satisfied for $R^{\prime }=R_{\alpha }^{\prime }\left(L\right)-\delta^{\prime }$, with

(A122) $$\begin{array}{c} R_{\alpha }^{\prime }\left(L\right)=\frac{1}{2}\log\left(\frac{(\gamma +\alpha +2\rho \sqrt{\alpha \gamma })\Omega +\Lambda }{(1-\rho^{2})\alpha \Omega +\Lambda }\right)=-\frac{1}{2}\log\left(\frac{(1-\rho^{2})\alpha \Omega +\Lambda }{(\gamma +\alpha +2\rho \sqrt{\alpha \gamma })\Omega +\Lambda }\right). \end{array}$$

and arbitrary $\delta^{\prime }>0$, if η and δ are sufficiently small.

As for the error event for $M_{b}^{\prime \prime }$, a similar derivation shows that the probability term in the last sum in (A98) exponentially tends to zero as n→∞, provided that

(A123) $$\begin{array}{cc} R^{\prime \prime }< & -\frac{1}{2}\log\left(1+\eta -\frac{\beta^{2}{\left(1-2\delta \right)}^{2}}{\beta^{2}+n\Lambda }\right)<-\frac{1}{2}\log\left(\eta +\frac{\Lambda }{\left(1-\rho^{2}\right)\alpha \left(\Omega -\delta \right)+\Lambda }\right). \end{array}$$

This is satisfied for $R^{\prime \prime }=R_{\alpha }^{\prime \prime }\left(L\right)-\delta^{\prime \prime }$, with

(A124) $$\begin{array}{c} R_{\alpha }^{\prime \prime }\left(L\right)=\frac{1}{2}\log\left(\frac{(1-\rho^{2})\alpha \Omega +\Lambda }{\Lambda }\right)=-\frac{1}{2}\log\left(\frac{\Lambda }{(1-\rho^{2})\alpha \Omega +\Lambda }\right) \end{array}$$

for an arbitrary $\delta^{\prime \prime }>0$, if η and δ are sufficiently small.

We have thus shown achievability of every rate

(A125) $$\begin{array}{c} R<\min\left\{R_{\alpha }^{\prime }\left(L\right)+R_{\alpha }^{\prime \prime }\left(L\right),\frac{1}{2}\log\left(1+\frac{(1-\alpha )\Omega }{\sigma^{2}}\right)+R_{\alpha }^{\prime \prime }\left(L\right)\right\}\;, \end{array}$$

where

(A126) $$\begin{array}{cc} R_{\alpha }^{\prime }\left(L\right)+R_{\alpha }^{\prime \prime }\left(L\right)= & \frac{1}{2}\log\left(\frac{(\gamma +\alpha +2\rho \sqrt{\alpha \gamma })\Omega +\Lambda }{(1-\rho^{2})\alpha \Omega +\Lambda }\right)+\frac{1}{2}\log\left(\frac{(1-\rho^{2})\alpha \Omega +\Lambda }{\Lambda }\right)\\ = & \frac{1}{2}\log\left(1+\frac{\Omega_{1}+\alpha \Omega +2\rho \sqrt{\alpha \Omega \cdot \Omega_{1}}}{\Lambda }\right) \end{array}$$

(see (A93)). This completes the proof of the lower bound.

### Appendix M.2. Upper Bound

Let R>0 be an achievable rate. Then, there exists a sequence of $\left(2^{nR},n,\varepsilon_{n}^{*}\right)$ codes $C_{n}=\left(f,f_{1},g\right)$ for the Gaussian AVRC L with SFD such that $\varepsilon_{n}^{*}\to 0$ as n→∞, where the encoder consists of a pair $f=(f^{\prime },f^{\prime \prime })$, with $f^{\prime }:\left[1:2^{nR}\right]\to R^{n}$ and $f^{\prime \prime }:\left[1:2^{nR}\right]\to R^{n}$. Assume without loss of generality that the codewords have zero mean, i.e.,

(A127) $$\begin{array}{c} \frac{1}{2^{nR}}\sum_{m=1}^{2^{nR}}\frac{1}{n}\sum_{i=1}^{n}f_{i}\left(m\right)=0\;,\;\\ \int_{-\infty }^{\infty }\;dy_{1}\cdot \frac{1}{2^{nR}}\sum_{m\in [1:2^{nR}]}P_{Y_{1}|M}\left(y_{1}|m\right)\cdot \frac{1}{n}\sum_{i=1}^{n}f_{1,i}\left(y_{1,1},y_{1,2},\ldots ,y_{1,i-1}\right)=0\;, \end{array}$$

where $P_{y_{1}|M}\left(y_{1}|m\right)=\frac{1}{{\left(2\pi \sigma^{2}\right)}^{MMn\;/\;2}}e^{-{∥y_{1}-f^{\prime \prime }\left(m\right)∥}^{2}/2\sigma^{2}}$. If this is not the case, redefine the code such that the mean is subtracted from each codeword. Then, define

(A128) $$\begin{array}{c} \alpha ≜\frac{1}{n\Omega }\cdot \frac{1}{2^{nR}}\sum_{m\in [1:2^{nR}]}{∥f^{\prime }\left(m\right)∥}^{2}\;,\\ \alpha_{1}≜\frac{1}{n\Omega_{1}}\cdot \frac{1}{2^{nR}}\sum_{m\in [1:2^{nR}]}\int_{-\infty }^{\infty }\;dy_{1}\cdot P_{Y_{1}|M}\left(y_{1}|m\right)\cdot {∥f_{1}\left(y_{1}\right)∥}^{2}.\\ \rho ≜\frac{1}{n\sqrt{\alpha \Omega \cdot \alpha_{1}\Omega_{1}}}\int_{-\infty }^{\infty }\;dy_{1}\cdot \frac{1}{2^{nR}}\sum_{m\in [1:2^{nR}]}P_{Y_{1}|M}\left(y_{1}|m\right)\cdot \langle f^{\prime }\left(m\right),f_{1}\left(y_{1}\right)\rangle \;, \end{array}$$

Since the code satisfies the input constraints Ω and $\Omega_{1}$, we have that α, $\alpha_{1}$ and ρ are in the interval [0,1].

First, we show that if

(A129) $$\begin{array}{c} \Lambda >\Omega_{1}+\alpha \Omega +2\rho \sqrt{\alpha \Omega \cdot \Omega_{1}}+\delta \;, \end{array}$$

then the capacity is zero, where δ>0 is arbitrarily small. Consider the following jamming strategy. The jammer draws a message $\tilde{M}\in \left[1:2^{nR}\right]$ uniformly at random, and then, generates a sequence ${\tilde{Y}}_{1}\in R^{n}$ distributed according to $P_{Y_{1}|M}\left({\tilde{y}}_{1}|\tilde{m}\right)$. Let $\tilde{S}=f^{\prime }\left(\tilde{M}\right)+f_{1}\left({\tilde{Y}}_{1}\right)$. If $\frac{1}{n}{∥\tilde{S}∥}^{2}\leq \Lambda$, the jammer chooses $\tilde{S}$ to be the state sequence. Otherwise, let the state sequence consist of all zeros. Observe that

(A130) $$\begin{array}{cc} E{∥\tilde{S}∥}^{2}= & E{∥f^{\prime }\left(\tilde{M}\right)+f_{1}\left({\tilde{Y}}_{1}\right)∥}^{2}\\ = & E{∥f^{\prime }\left(\tilde{M}\right)∥}^{2}+E{∥f_{1}\left({\tilde{Y}}_{1}\right)∥}^{2}+2E\langle f\left(\tilde{M}\right),f_{1}\left({\tilde{Y}}_{1}\right)\rangle \\ = & n(\alpha \Omega +\alpha_{1}\Omega_{1}+2\rho \sqrt{\alpha \Omega \cdot \alpha_{1}\Omega_{1}})\\ \leq & n\left(\alpha \Omega +\Omega_{1}+2\rho \sqrt{\alpha \Omega \cdot \Omega_{1}}\right)<n\left(\Lambda -\delta \right). \end{array}$$

where the second equality is due to (A128), and the last inequality is due to (A129). Thus, by Chebyshev’s inequality, there exists κ>0 such that

(A131) $$\begin{array}{c} \mathrm{Pr}\left(\frac{1}{n}{∥\tilde{S}∥}^{2}\leq \Lambda \right)\geq \kappa . \end{array}$$

The state sequence S is then distributed according to

(A132) $$\begin{array}{c} P_{S|\{\frac{1}{n}{∥\tilde{S}∥}^{2}\leq \Lambda \}}\left(s\right)=\frac{1}{2^{nR}}\sum_{\tilde{m}\in \left[1:2^{nR}\right]}\int_{{\tilde{y}}_{1}:f^{\prime }\left(\tilde{m}\right)+f_{1}\left({\tilde{y}}_{1}\right)=s}\;dy_{1}P_{Y_{1}|M}\left(y_{1}|m\right)\;,\\ \mathrm{Pr}\left(S=0|\frac{1}{n}{∥\tilde{S}∥}^{2}>\Lambda \right)=1. \end{array}$$

Assume to the contrary that a positive rate can be achieved when the channel is governed by such state sequence, hence the size of the message set is at least 2, i.e., $M≜2^{nR}\geq 2$. The probability of error is then bounded by

(A133) $$\begin{array}{cc} P_{e}^{\left(n\right)}\left(q,C\right)= & \int_{-\infty }^{\infty }\;ds\cdot q\left(s\right)P_{e|s}^{\left(n\right)}\left(C\right)\geq \mathrm{Pr}\left(\frac{1}{n}{∥\tilde{S}∥}^{2}\leq \Lambda \right)\cdot \int_{s:\frac{1}{n}{∥s∥}^{2}\leq \Lambda }\;ds\cdot P_{S|\{\frac{1}{n}{∥\tilde{S}∥}^{2}\leq \Lambda \}}\left(s\right)\cdot P_{e|s}^{\left(n\right)}\left(C\right)\\ \geq & \kappa \cdot \int_{s:\frac{1}{n}{∥s∥}^{2}\leq \Lambda }\;ds\cdot P_{S|\{\frac{1}{n}{∥\tilde{S}∥}^{2}\leq \Lambda \}}\left(s\right)\cdot P_{e|s}^{\left(n\right)}\left(C\right) \end{array}$$

where the inequality holds by (A131). Next, we have that

(A134) $$\begin{array}{cc} P_{e|s}^{\left(n\right)}\left(C\right)= & \frac{1}{M}\sum_{m=1}^{M}\int_{-\infty }^{\infty }\;dy_{1}\cdot P_{Y_{1}|M}\left(y_{1}|m\right)\cdot 1\left\{y_{1}:g\left(f^{\prime }\left(m\right)+f_{1}\left(y_{1}\right)+s\right)\neq m\right\}\;, \end{array}$$

where we define the indicator function $G\left(y_{1}\right)=1\left\{y_{1}\in A\right\}$ such that $G(y_{1})=1$ if $y_{1}\in A$, and $G(y_{1})=0$ otherwise. Substituting (A132) and (A134) into (A133) yields

(A135) $$\begin{array}{cc} P_{e}^{\left(n\right)}\left(q,C\right)\geq & \kappa \cdot \int_{s:\frac{1}{n}{∥s∥}^{2}\leq \Lambda }\;ds\cdot \frac{1}{M}\sum_{\tilde{m}=1}^{M}\int_{{\tilde{y}}_{1}:f^{\prime }\left(\tilde{m}\right)+f_{1}\left({\tilde{y}}_{1}\right)=s}\;d{\tilde{y}}_{1}\cdot P_{Y_{1}|M}\left({\tilde{y}}_{1}|\tilde{m}\right)\\ & \times \frac{1}{M}\sum_{m=1}^{M}\int_{-\infty }^{\infty }\;dy_{1}\cdot P_{Y_{1}|M}\left(y_{1}|m\right)\cdot 1\left\{y_{1}:g\left(f^{\prime }\left(m\right)+f_{1}\left(y_{1}\right)+s\right)\neq m\right\}. \end{array}$$

Eliminating $s=f^{\prime }\left(\tilde{m}\right)+f_{1}\left({\tilde{y}}_{1}\right)$, and adding the constraint ${∥f^{\prime }\left(m\right)+f_{1}\left(y_{1}\right)∥}^{2}\leq \Lambda$, we obtain the following,

(A136) $$\begin{array}{cc} P_{e}^{\left(n\right)}\left(q,C\right)\geq & \frac{\kappa }{M^{2}}\sum_{m=1}^{M}\sum_{\tilde{m}=1}^{M}\int_{\begin{array}{c} \left(y_{1}\;,\;{\tilde{y}}_{1}\right)\;:\;\frac{1}{n}{∥f^{\prime }\left(m\right)+f_{1}\left(y_{1}\right)∥}^{2}\leq \Lambda \;,\\ \frac{1}{n}{∥f^{\prime }\left(\tilde{m}\right)+f_{1}\left({\tilde{y}}_{1}\right)∥}^{2}\leq \Lambda \end{array}}\;dy_{1}\;d{\tilde{y}}_{1}\cdot P_{Y_{1}|M}\left(y_{1}|m\right)P_{Y_{1}|M}\left({\tilde{y}}_{1}|\tilde{m}\right)\\ & \times 1\left\{y_{1}:g\left(f^{\prime }\left(m\right)+f_{1}\left(y_{1}\right)+f^{\prime }\left(\tilde{m}\right)+f_{1}\left({\tilde{y}}_{1}\right)\right)\neq m\right\}. \end{array}$$

Now, by interchanging the summation variables $\left(m,y_{1}\right)$ and $\left(\tilde{m},{\tilde{y}}_{1}\right)$, we have that

$$\begin{array}{cc} P_{e}^{\left(n\right)}\left(q,C\right)\geq & \frac{\kappa }{2M^{2}}\sum_{m=1}^{M}\sum_{\tilde{m}=1}^{M}\int_{\begin{array}{c} \left(y_{1}\;,\;{\tilde{y}}_{1}\right)\;:\;\frac{1}{n}{∥f^{\prime }\left(m\right)+f_{1}\left(y_{1}\right)∥}^{2}\leq \Lambda \;,\\ \frac{1}{n}{∥f^{\prime }\left(\tilde{m}\right)+f_{1}\left({\tilde{y}}_{1}\right)∥}^{2}\leq \Lambda \end{array}}\;dy_{1}\;d{\tilde{y}}_{1}\cdot P_{Y_{1}|M}\left(y_{1}|m\right)P_{Y_{1}|M}\left({\tilde{y}}_{1}|\tilde{m}\right) \end{array}$$

(A137) $$\begin{array}{cc} & \times 1\left\{y_{1}:g\left(f^{\prime }\left(m\right)+f_{1}\left(y_{1}\right)+f^{\prime }\left(\tilde{m}\right)+f_{1}\left({\tilde{y}}_{1}\right)\right)\neq m\right\}\\ + & \frac{\kappa }{2M^{2}}\sum_{m=1}^{M}\sum_{\tilde{m}=1}^{M}\int_{\begin{array}{c} \left(y_{1}\;,\;{\tilde{y}}_{1}\right)\;:\;\frac{1}{n}{∥f^{\prime }\left(m\right)+f_{1}\left(y_{1}\right)∥}^{2}\leq \Lambda \;,\\ \frac{1}{n}{∥f^{\prime }\left(\tilde{m}\right)+f_{1}\left({\tilde{y}}_{1}\right)∥}^{2}\leq \Lambda \end{array}}\;dy_{1}\;d{\tilde{y}}_{1}\cdot P_{Y_{1}|M}\left(y_{1}|m\right)P_{Y_{1}|M}\left({\tilde{y}}_{1}|\tilde{m}\right)\\ & \times 1\left\{y_{1}:g\left(f^{\prime }\left(m\right)+f_{1}\left(y_{1}\right)+f^{\prime }\left(\tilde{m}\right)+f_{1}\left({\tilde{y}}_{1}\right)\right)\neq \tilde{m}\right\}. \end{array}$$

Thus,

(A138) $$\begin{array}{cc} P_{e}^{\left(n\right)}\left(q,C\right)\geq & \frac{\kappa }{2M^{2}}\sum_{m=1}^{M}\sum_{\tilde{m}\neq m}\int_{\begin{array}{c} \left(y_{1}\;,\;{\tilde{y}}_{1}\right)\;:\;\frac{1}{n}{∥f^{\prime }\left(m\right)+f_{1}\left(y_{1}\right)∥}^{2}\leq \Lambda \;,\\ \frac{1}{n}{∥f^{\prime }\left(\tilde{m}\right)+f_{1}\left({\tilde{y}}_{1}\right)∥}^{2}\leq \Lambda \end{array}}\;dy_{1}\;d{\tilde{y}}_{1}\cdot P_{Y_{1}|M}\left(y_{1}|m\right)P_{Y_{1}|M}\left({\tilde{y}}_{1}|\tilde{m}\right)\\ & \times [1\left\{y_{1}:g\left(f^{\prime }\left(m\right)+f_{1}\left(y_{1}\right)+f^{\prime }\left(\tilde{m}\right)+f_{1}\left({\tilde{y}}_{1}\right)\right)\neq m\right\}\\ & \;+1\left\{y_{1}:g\left(f^{\prime }\left(m\right)+f_{1}\left(y_{1}\right)+f^{\prime }\left(\tilde{m}\right)+f_{1}\left({\tilde{y}}_{1}\right)\right)\neq \tilde{m}\right\}]. \end{array}$$

As the sum in the square brackets is at least 1 for all $\tilde{m}\neq m$, it follows that

(A139) $$\begin{array}{cc} P_{e}^{\left(n\right)}\left(q,C\right)\geq & \frac{\kappa }{2M^{2}}\sum_{m=1}^{M}\sum_{\tilde{m}\neq m}\int_{\begin{array}{c} \left(y_{1}\;,\;{\tilde{y}}_{1}\right)\;:\;\frac{1}{n}{∥f^{\prime }\left(m\right)+f_{1}\left(y_{1}\right)∥}^{2}\leq \Lambda \;,\\ \frac{1}{n}{∥f^{\prime }\left(\tilde{m}\right)+f_{1}\left({\tilde{y}}_{1}\right)∥}^{2}\leq \Lambda \end{array}}\;dy_{1}\;d{\tilde{y}}_{1}\cdot P_{Y_{1}|M}\left(y_{1}^{n}|m\right)P_{Y_{1}|M}\left({\tilde{y}}_{1}|\tilde{m}\right)\\ \geq & \frac{\kappa }{4}\cdot \mathrm{Pr}\left(\begin{array}{c} \frac{1}{n}{∥f^{\prime }\left(M\right)+f_{1}\left(Y_{1}\right)∥}^{2}\leq \Lambda \;,\\ \frac{1}{n}{∥f^{\prime }\left(\tilde{M}\right)+f_{1}\left({\tilde{Y}}_{1}\right)∥}^{2}\leq \Lambda \;,\;\tilde{M}\neq M \end{array}\right). \end{array}$$

Then, recall that by (A130), the expectation of $\frac{1}{n}{∥f^{\prime }\left(M\right)+f_{1}\left(Y_{1}^{n}\right)∥}^{2}$ is strictly lower than Λ, and for a sufficiently large *n*, the conditional expectation of $\frac{1}{n}\left|\right|f^{\prime }\left(\tilde{M}\right)+f_{1}\left({\tilde{Y}}_{1}\right){\left|\right|}^{2}$ given $\tilde{M}\neq M$ is also strictly lower than Λ. Thus, by Chebyshev’s inequality, the probability of error is bounded from below by a positive constant. Following this contradiction, we deduce that if the code is reliable, then $\Lambda \leq (1+\alpha +2\rho \sqrt{\alpha })\Omega$.

It is left for us to show that for α and ρ as defined in (A128), we have that $R<F_{G}\left(\alpha ,\rho \right)$ (see (36)). For a $\left(2^{nR},n,\varepsilon_{n}^{*}\right)$ code,

(A140) $$\begin{array}{c} P_{e|s}^{\left(n\right)}\left(C\right)\leq \varepsilon_{n}^{*}\;, \end{array}$$

for all $s\in R^{n}$ with ${∥s∥}^{2}\leq n\Lambda$. Then, consider using the code C over the Gaussian relay channel $W_{Y,Y_{1}|X,X_{1}}^{\bar{q}}$, specified by

(A141) $$\begin{array}{c} Y_{1}=X^{\prime \prime }+Z\;,\\ Y=X^{\prime }+X_{1}+\bar{S}\;, \end{array}$$

where the sequence $\bar{S}$ is i.i.d. $∼\bar{q}=N\left(0,\Lambda -\delta \right)$. First, we show that the code C is reliable for this channel, and then we show that $R<F_{G}\left(\alpha ,\rho \right)$. Using the code C over the channel $W_{Y,Y_{1}|X,X_{1}}^{\bar{q}}$, the probability of error is bounded by

(A142) $$\begin{array}{c} P_{e}^{\left(n\right)}\left(\bar{q},C\right)=\mathrm{Pr}\left(\frac{1}{n}∥\bar{S}∥>\Lambda \right)+\int_{s:\frac{1}{n}∥\bar{S}∥\leq \Lambda }\;ds\cdot P_{e|s}^{\left(n\right)}\left(C\right)\leq \varepsilon_{n}^{*}+\varepsilon_{n}^{**}\;, \end{array}$$

where we have bounded the first term by $\varepsilon_{n}^{**}$ using the law of large numbers and the second term using (A140), where $\varepsilon_{n}^{**}\to 0$ as n→∞. Since $W_{Y,Y_{1}|X,X_{1}}^{\bar{q}}$ is a channel without a state, we can now show that $R<F_{G}\left(\alpha ,\rho \right)$ by following the lines of [4] and [5]. By Fano’s inequality and [4] (Lemma 4), we have that

(A143) $$\begin{array}{cc} R\leq & \;\frac{1}{n}\sum_{i=1}^{n}I_{\bar{q}}\left(X_{i}^{\prime },X_{i}^{\prime \prime },X_{1,i};Y_{i}\right)+\varepsilon_{n}\;,\\ R\leq & \;\frac{1}{n}\sum_{i=1}^{n}I_{\bar{q}}\left(X_{i}^{\prime },X_{i}^{\prime \prime };Y_{i},Y_{1,i}|X_{1,i}\right)+\varepsilon_{n}\;, \end{array}$$

where $\bar{q}=N\left(0,\Lambda -\delta \right)$, $X^{\prime }=f^{\prime }\left(M\right)$, $X^{\prime \prime }=f^{\prime \prime }\left(M\right)$, $X_{1}=f_{1}\left(y_{1}\right)$, and $\varepsilon_{n}\to 0$ as n→∞. For the Gaussian relay channel with SFD, we have the following Markov relations,

(A144) $$\begin{array}{c} Y_{1,i}-X_{i}^{\prime \prime }-\left(X_{i}^{\prime },X_{1,i},Y_{1,i}\right)\;, \end{array}$$

(A145) $$\begin{array}{c} \left(X_{i}^{\prime \prime },Y_{1,i}\right)-\left(X_{i}^{\prime },X_{1,i}\right)-Y_{i}. \end{array}$$

Hence, by (A145), $I_{\bar{q}}\left(X_{i}^{\prime },X_{i}^{\prime \prime },X_{1,i};Y_{i}\right)=I_{\bar{q}}\left(X_{i}^{\prime },X_{1,i};Y_{i}\right)$. Moving to the second bound in the RHS of (A143), we follow the lines of [5]. Then, by the mutual information chain rule, we have

(A146) $$\begin{array}{cc} I_{\bar{q}}\left(X_{i}^{\prime },X_{i}^{\prime \prime };Y_{i},Y_{1,i}|X_{1,i}\right)= & I\left(X_{i}^{\prime \prime };Y_{1,i}|X_{1,i}\right)+I\left(X_{i}^{\prime };Y_{1,i}|X_{i}^{\prime \prime },X_{1,i}\right)+I_{\bar{q}}\left(X_{i}^{\prime },X_{i}^{\prime \prime };Y_{i}|X_{1,i},Y_{1,i}\right)\\ \overset{\left(a\right)}{=} & I\left(X_{i}^{\prime \prime };Y_{1,i}|X_{1,i}\right)+I_{\bar{q}}\left(X_{i}^{\prime },X_{i}^{\prime \prime };Y_{i}|X_{1,i},Y_{1,i}\right)\\ \overset{\left(b\right)}{=} & \left[H\left(Y_{1,i}|X_{1,i}\right)-H\left(Y_{1,i}|X_{i}^{\prime \prime }\right)\right]+\left[H_{\bar{q}}\left(Y_{i}|X_{1,i},Y_{1,i}\right)-H_{\bar{q}}\left(Y_{i}|X_{i}^{\prime },X_{1,i}\right)\right]\\ \overset{\left(c\right)}{\leq } & I_{q_{1}}\left(X_{i}^{\prime \prime };Y_{1,i}\right)+I\left(X_{i}^{\prime };Y_{i}|X_{1,i}\right) \end{array}$$

where (a) is due to (A144), (b) is due to (A145), and (c) holds since conditioning reduces entropy. Introducing a time-sharing random variable K∼Unif[1:n], which is independent of $X^{\prime }$, $X^{\prime \prime }$, $X_{1}$, Y, $Y_{1}$, we have that

(A147) $$\begin{array}{cc} R-\varepsilon_{n}\leq & \;I_{\bar{q}}\left(X_{K}^{\prime },X_{1,K};Y_{K}|K\right)\\ R-\varepsilon_{n}\leq & I\left(X_{K}^{\prime \prime };Y_{1,K}|K\right)+I_{\bar{q}}\left(X_{K}^{\prime };Y_{K}|X_{1,K},K\right). \end{array}$$

Now, by the maximum differential entropy lemma (see e.g., [98] (Theorem 8.6.5)),

(A148) $$\begin{array}{cc} I_{\bar{q}}\left(X_{K}^{\prime },X_{1,K};Y_{K}|K\right)\leq & \frac{1}{2}\log\left(\frac{E\left[{\left(X_{K}^{\prime }+X_{1,K}\right)}^{2}\right]+\left(\Lambda -\delta \right)}{\Lambda -\delta }\right)=\frac{1}{2}\log\left(1+\frac{\alpha \Omega +\alpha_{1}\Omega_{1}+2\rho \sqrt{\alpha \Omega \cdot \alpha_{1}\Omega_{1}}}{\Lambda -\delta }\right) \end{array}$$

and

(A149) $$\begin{array}{cc} I\left(X_{K}^{\prime \prime };Y_{1,K}|K\right)+I_{\bar{q}}\left(X_{K}^{\prime };Y_{K}|X_{1,K},K\right)\leq & \frac{1}{2}\log\frac{EX_{K}^{\prime \prime 2}+\sigma^{2}}{\sigma^{2}}+\frac{1}{2}\log\frac{\left[1-\frac{{\left(E\left(X_{K}^{\prime }\cdot X_{1,K}\right)\right)}^{2}}{EX_{K}^{\prime 2}\cdot EX_{1,K}^{2}}\right]EX_{K}^{\prime 2}+\left(\Lambda -\delta \right)}{\Lambda -\delta }\\ = & \frac{1}{2}\log\left(1+\frac{(1-\alpha )\Omega }{\sigma^{2}}\right)+\frac{1}{2}\log\left(1+\frac{(1-\rho^{2})\alpha \Omega }{\Lambda -\delta }\right)\;, \end{array}$$

where α, $\alpha_{1}$ and ρ are given by (A128). Since δ>0 is arbitrary, and $\alpha_{1}\leq 1$, the proof follows from (A147)–(A149). □
